# OrchardQuant‐3D: combining drone and LiDAR to perform scalable 3D phenotyping for characterising key canopy and floral traits in fruit orchards

**DOI:** 10.1111/pbi.70229

**Published:** 2025-07-23

**Authors:** Yunpeng Xia, Hanghang Li, Fanhang Zhang, Gang Sun, Kaijie Qi, Robert Jackson, Felipe Pinheiro, Xiaoman Liu, Yue Mu, Shaoling Zhang, Greg Deakin, E. Charles Whitfield, Shutian Tao, Ji Zhou

**Affiliations:** ^1^ College of Engineering, Academy for Advanced Interdisciplinary Studies, Plant Phenomics Research Centre Nanjing Agricultural University Nanjing China; ^2^ College of Horticulture, State Key Laboratory of Crop Genetics and Germplasm Enhancement Nanjing Agricultural University Nanjing China; ^3^ Data Sciences, East Malling Research (EMR), Crop Science Centre (CSC) National Institute of Agricultural Botany (NIAB) Cambridge UK; ^4^ College of Sciences Nanjing Agricultural University Nanjing China

**Keywords:** multi‐source 3D phenotyping, canopy morphological features, floral and fruit traits, automated trait analysis, fruit orchards

## Abstract

Orchard fruits such as pear and apple are important for ensuring global food security and agricultural economy as they not only provide essential nutrients, but also support biodiversity and ecosystem services. Breeders, growers and plant researchers constantly study desirable tree morphological features and floral characteristics to ensure fruit production and quality. Still, traditional orchard phenotyping is often laborious, limited in scale and prone‐to‐error, resulting in many attempts to develop reliable and scalable toolkits to address this challenge. Here, we present OrchardQuant‐3D, an analytic pipeline for automating tree‐level analysis of key canopy and floral traits for different types of fruit orchards. We first built a data fusion algorithm to register 3D point clouds collected by both drones (for colour signals) and Light Detection And Ranging (LiDAR, for precise spatial properties), reconstructing high‐quality 3D orchard models at different growth stages. Then, we utilised precise global navigation satellite system signals to position trees in orchards with millimetre‐level accuracy, enabling tree‐level analysis of key canopy (e.g. crown volume and the number or branches) and floral traits (e.g. blossom clusters and volumes) using 3D computer vision, complex graph theory and feature engineering techniques. Equipped with the OrchardQuant‐3D pipeline, we successfully measured varietal differences of four pear cultivars from a small pear orchard in Nanjing China, followed by a scale‐up study that surveyed 3D tree morphologies, key floral and fruit traits from 1104 apple trees in an orchard in East Malling, United Kingdom. To the best of our knowledge, such a multi‐source, comprehensive and expandable methodology has not yet been introduced to this important research domain. Hence, we believe that our work demonstrates a step change in our ability to conduct scalable 3D orchard phenotyping, which is highly valuable to advance orchard breeding, precise tree management and orchard research greatly to sustain fruit tree production in a rapidly changing climate.

## Introduction

Orchard fruits play a crucial role in our food security and agricultural economy around the world (Montanaro *et al*., 2017). Hard fruits such as pear (*Pyrus communis* L.) and apple (*Malus domestica* Borkh.) not only provide essential nutrients (Vincente *et al*., 2014), but also support biodiversity and ecosystem services (Demestihas *et al*., 2017; Horak *et al*., 2013). As two of the most produced and consumed fruits, pear and apple are widely grown in temperate regions, with hundreds of varieties cultivated globally (Li *et al*., 2022; Reganold *et al*., 2001) featuring an extensive number of morphological and physiological traits (Mupambi *et al*., 2018; Wu *et al*., 2018). Wide‐ranging applications of edible fruits and tree wood led to diverse fundamental and applied studies for the hard fruits, including: (1) genetic improvement of desired yield and quality traits such as fruit size, shape and skin colour to facilitate tree breeding and growing (Harris *et al*., 2002; Wu *et al*., 2013); (2) the enhancement of nutritional value of fruits through biochemical composition and nutrient contents (Kumar *et al*., 2018; Tan *et al*., 2019); (3) molecular mechanistic research such as lignin biosynthesis (Hu *et al*., 2021; Wang *et al*., 2022) and fruit development (Ng *et al*., 2013; Xu *et al*., 2021); (4) hormonal regulation on endogenous hormones and sugar content (Tian *et al*., 2021; Ulger *et al*., 2004); and (5) precise orchard management to monitor insect pollinators (Fountain *et al*., 2019; Garratt *et al*., 2014), control pest and disease (Damos *et al*., 2015; Shaw *et al*., 2021) and select preferred characteristics (e.g. appearance and flavour) to maximise marketable yield (Florkowski and Łysiak, 2015; Musacchi and Serra, 2018).

Phenotyping plays an essential role in many of these studies mentioned above. It enables plant researchers, breeders and growers to measure and identify agronomically important traits (Huang *et al*., 2020). For example, the assessment of plant responses to environmental stresses such as drought, salinity and temperature fluctuations (Chen *et al*., 2021), resistance to disease and pest (Simionca Mărcășan *et al*., 2023) and varietal differences in growth habits (Cornille *et al*., 2012; Gallinger *et al*., 2021). Additionally, key climatic factors such as cumulative growing temperature and solar radiation were combined with phenological traits (e.g. growth stages) to study both orchard‐ and tree‐level photosynthetic capacities and energy distribution across multiple seasons (Paproki *et al*., 2012; Zhang *et al*., 2021). Due to the importance of florescence in the formation of fruit yield, much attention was paid towards studying dynamic relationships between tree canopy and floral traits in recent years (Hünicken *et al*., 2020; Kviklys *et al*., 2022). For instance, without mitigation, excessive flowering can lead to overloaded trees that will result in undersized fruits and alternate bearing (Samach and Smith, 2013), whereas insufficient flowers likely lead to poor yield and tree health issues (Breen *et al*., 2016; Zhang *et al*., 2023). Hence, the importance of measuring flower density and fruit load is widely recognised by industry and academia, resulting in research on both canopy structure (e.g. canopy height and crown size) and key floral features (e.g. the number of blossom clusters) in order to optimise pruning and flower thinning for a sustainable fruit orchard (Bound and Corelli‐Grappadelli, 2021; Jonkers, 1979; Tustin *et al*., 2022). Nevertheless, due to lack of suitable orchard‐ and tree‐level phenotyping and analytic toolkits, it is still challenging to quantify the above desirable traits in a scalable manner (Bian *et al*., 2022; Huang *et al*., 2020), indicating the necessity of introducing capable and scalable methodologies to bridge the gap.

Due to the complex three‐dimensional (3D) structure of orchard trees, manual assessment of orchard‐ and tree‐level morphological features is often time‐consuming, small scale and prone‐to‐error (Colaço *et al*., 2018; Shang *et al*., 2023). This led to many attempts to develop two‐dimensional (2D) and three‐dimensional (3D) mapping techniques to address this challenge. For 2D‐based approaches, handheld red‐green‐blue (RGB) digital cameras were used to collect tree‐level fruit tree blossoms (e.g. apple), so that flower density and yield production could be estimated (Aggelopoulou *et al*., 2011; Tian *et al*., 2020; Zhang *et al*., 2023); drone‐mounted RGB sensors (Chakraborty *et al*., 2023; Tubau Comas *et al*., 2019) and multi‐ and hyper‐spectral image sensors were also employed to quantify branching structure, photosynthetic effects and flowers (Horton *et al*., 2017; Xiao *et al*., 2014; Zhang *et al*., 2020). For 3D‐based methods, a range of solutions were developed, including *FruitNeRF* (based on semantic neural radiance fields, NeRFs) for counting fruits in semantic images (Meyer *et al*., 2024), DaSNet‐V2 (combining instance segmentation of fruits and semantic segmentation of branch) for detecting fruits in RGB‐D (with depth information) imagery (Kang and Chen, 2020). Many of these methods are based on photogrammetry algorithms such as structure from motion (SfM) and multi‐view stereo vision (MVS), which convert RGB image series into 3D coloured point clouds (Hobart *et al*., 2020; Sinha *et al*., 2022), so that tree‐level spatial and colour information can be utilised to quantify tree canopies, flower clusters and fruits (Gené‐Mola *et al*., 2020; Jiang *et al*., 2020; Van Brabant *et al*., 2020).

Additionally, different Light Detection and Ranging (LiDAR) systems were employed in 3D tree mapping, advancing the development of various quantitative structure modelling (QSM) methods, including: (1) TreeQSM, one of the earliest and widely used QSM‐based tools, which measures topological branching structures from different types of trees (Raumonen *et al*., 2013); (2) AdTree, a 3D reconstruction method for robust tree modelling, so that a range of tree attributes can be estimated (Du *et al*., 2019); (3) AppleQSM, a QSM‐based pipeline for tree‐level instance segmentation, focusing on tree‐ and branch‐level structural characterisation by employing a skeleton‐based approach (Qiu *et al*., 2024). Besides the QSM‐powered methods, other algorithms such as the hidden Markov model (Underwood *et al*., 2015) were also applied to examine tree morphological features. The above 2D‐ and 3D‐based solutions have significantly advanced 3D tree reconstruction, and structural, floral, and fruit analysis for different types of trees.

Still, many of these solutions require relatively complex adjustments of parameters (e.g. QSM for unevenly tree growth) and are often limited to a single data source, which creates a trade‐off between speed and accuracy (e.g. LiDAR may be highly accurate but is slower to survey compared to aerial imaging). These limitations indicate that many published solutions may not be easily applied to assess large scale and different types of orchards. Moreover, although vision‐based machine learning (ML) and deep learning (DL) techniques such as semantic or instance segmentation have been applied to cluster 3D point clouds for characterising tree structures and flowers (Le Louëdec and Cielniak, 2021; Tong *et al*., 2022; Xi *et al*., 2018), some of which also applied to study occluded branches, leaves and fruits (Chen *et al*., 2021), the bottleneck of ML/DL is still the dependence of high‐quality annotated datasets, expensive computing resources for model training and the generalisation of trained models for different types of trees, preventing many DL/ML‐powered toolkits from being effective adopted by the community (Le Louëdec and Cielniak, 2021; Yang and Xu, 2021; Zhang *et al*., 2023).

Due to the necessity of improving orchard productivity and fruit production in a rapidly changing climate, it is of great importance to develop an effective and scalable methodology that can quantify target traits using spatial and colour information collected from different types of orchards. By linking high‐throughput 3D mapping to other methodological advances (e.g. computer vison and ML/DL techniques), such an approach will improve efficiency, accuracy and automation in tree breeding, mitigate tree stresses under complex field conditions, and facilitate semi‐ or fully automated orchard management. To achieve the above goal, we present OrchardQuant‐3D, an automated and scalable analysis pipeline developed to combine both drone‐derived and LiDAR‐collected 3D points to perform tree‐level morphological and colour analysis, leading to reliable measures of key canopy, floral and even fruit traits for different sizes of pear and apple orchards. Building on the two mapping approaches, a data fusion algorithm was first developed to register 3D points collected by drones and LiDAR, which combined colour and spatial information with the association of LiDAR‐recorded global navigation satellite system (GNSS) signals to position trees with a millimetre (mm) level accuracy. After that, complex 3D graph theory and ML‐powered feature engineering were employed to quantify tree‐level canopy features (e.g. crown volume and the number or branches) using LiDAR‐collected signals and key floral traits (e.g. blossom clusters) based on drone‐collected 3D colour points. This enabled the quantification of varietal differences of different pear cultivars. To demonstrate the scalability of the pipeline, a scale‐up study of over a thousand apple trees in a high tree density orchard in the United Kingdom was accomplished, measuring tree‐level canopy, floral and fruit features that are key to fruit production. To the best of our knowledge, such a multi‐source and comprehensive methodology has not yet been introduced to this important research domain, which is highly likely leading to a step change in our ability to perform scalable 3D phenotyping for orchard breeding and precise tree management.

## Materials and Methods

### Plant materials and field experiments

**Figure 1:**
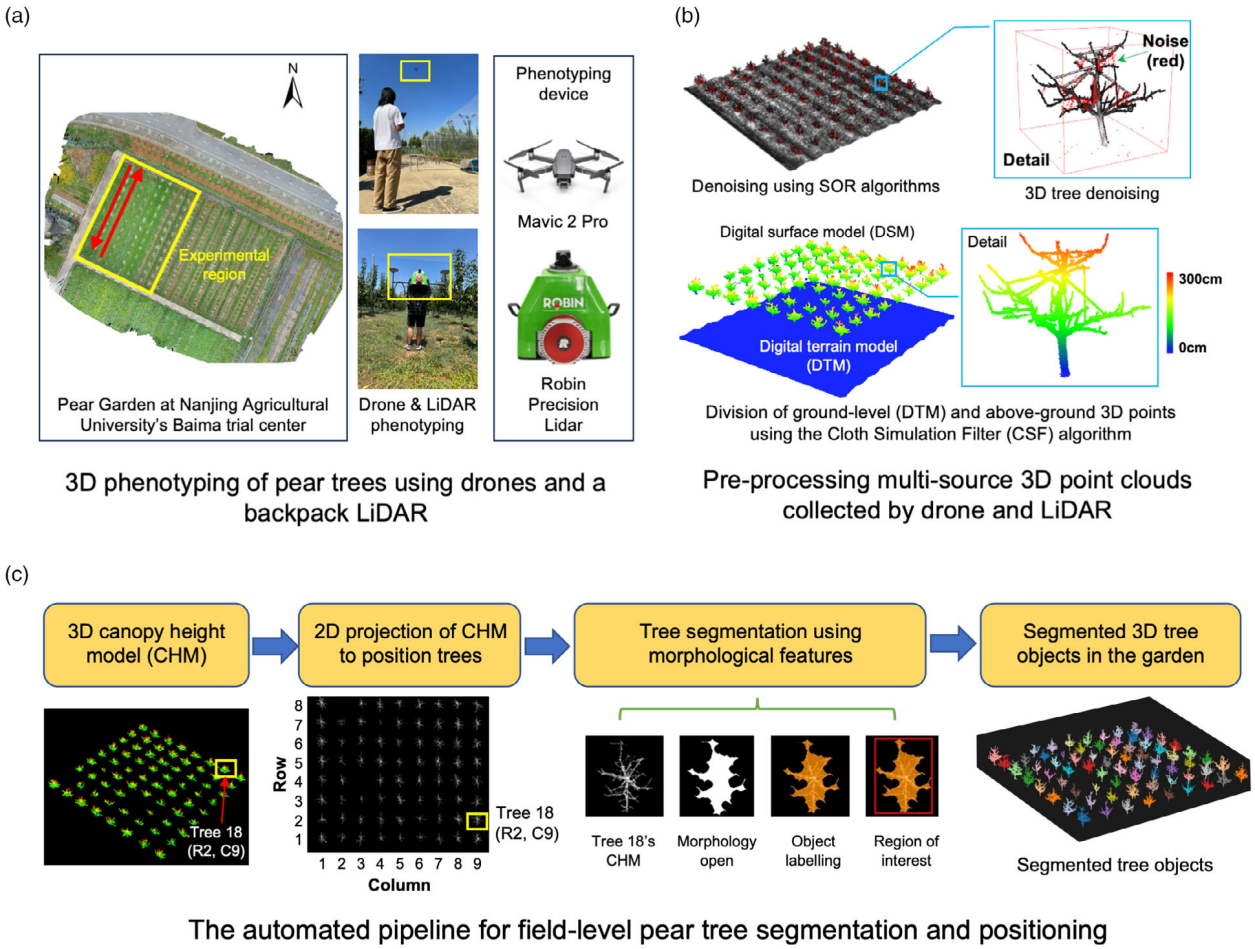
Multi‐source 3D orchard phenotyping using cost‐effective drones and a backpack LiDAR. (a) 3D orchard phenotyping using drone and LiDAR in a pear orchard in Nanjing China. (b) Pre‐processing of drone‐derived and LiDAR‐collected 3D point clouds. (c) An analysis pipeline established for automating tree segmentation in the orchard.

To facilitate the development of the OrchardQuant‐3D pipeline, we first established a field experiment in a demonstration pear orchard at the National Pear Industry Technology R&D Center, in Nanjing Agricultural University's (NAU) Baima trial centre, Jiangsu China (31°37′04.6″ N, 119°10′14.3″ E). The experimental field was around 0.16 ha in size (8 rows × 9 columns; yellow‐coloured rectangle, Figure 1a), consisting of 70 pear trees transplanted in January 2017 (a 3.5 m row spacing and 4.0 m tree spacing). Four East Asia pear varieties (*Pyrus pyrifolia*), Cuiguan (22 trees), Cuiyu (17), Xialu (15) and Housui (16) were studied, which were up to 2.6 m tall and cultivated for local climatic conditions. The four varieties are known for dissimilar canopy morphologies and slightly different developmental paces (Nishio *et al*., 2011; Xue *et al*., 2018).

In the United Kingdom, a large‐scale case study was conducted in an apple orchard with around 1280 trees (16 rows × 80 trees with tree gaps). We removed two edge rows due to data quality (e.g. gaps and dead trees) and retained 1104 trees (14 rows; approximately 0.5 ha in size) for the study. The orchard is located at the National Institute of Agricultural Botany's (NIAB) East Malling site (51°17′16.2″ N, 0°27′12.5″ E; Kent England), which contains two varieties (Galaxy Gala and Hillwell Braeburn on M9 rootstocks). Trees were planted in May 2009, with a 3.5 m row spacing and 1.0 m tree spacing. The canopy is supported by posts and wires with a height up to 3.0 m (Shuttleworth *et al*., 2023). In both countries, trees were managed following standard husbandry practices, suitable agronomic inputs for water and fertilisers, and pest and disease controls applied according to local requirements.

### Multi‐source 3D orchard mapping using drone and LiDAR

To achieve high‐quality 3D reconstruction of orchards, we established a multi‐source 3D mapping approach in both countries. In China, a cost‐effective drone (Mavic 2 Pro; DJI, Shenzhen, China) and a backpack LiDAR (Robin Precision; GeoSLAM, Nottingham, United Kingdom; with a 330° field of view, FOV, for scanning pear trees with a maximum height of 3 m) were combined to precisely collect tree‐level colour and spatial properties at different growth stages. The Chinese mapping was conducted in NAU's pear orchard within a fixed region (highlighted with a yellow rectangle; Figure 1a). The mapping was conducted between bud burst and pre‐inflorescence stages (i.e. from February to March 2023; BBCH‐13 to 39) (Meier *et al*., 2009), as well as the flowering stage (the last week of March 2023, 7–8 days after the initiation of the first blossom; BBCH‐57 to 65). Similar to China, the UK mapping was conducted with a SICK LMS 100 LiDAR (Hexagon, Morton, United States; with a 270° FOV) mounted on a tractor for mapping apple trees with a maximum height of 3.5 m, at the pre‐inflorescence stage (BBCH‐31 to 39) and with real‐time kinematic positioning (RTK) information recorded (Whitfield *et al*., 2022). A Phantom 4 drone (DJI, Shenzhen, China) was used for orchard mapping during the flowering stages, that is, 16/04/2024 (just before peak blossom; BBCH‐63 to 64) and 24/04/2024 (petal fall; BBCH‐65). Because orchard mapping, data pre‐processing and trait analysis for the China–UK data followed the same procedure, we chose to detail algorithmic steps based on the Chinese data to reduce duplication.

Drone‐based phenotyping followed a customised plan with pre‐determined flight parameters such as altitudes, camera angles and double‐grid flight path (Note S1). The mapping speed was set to around 0.3 ha·h^−1^ (i.e. 0.5 h for the 70 trees), with the acquired images processed by the Pix4Dmapper software (Pix4D, Lausanne, Switzerland) to reconstruct global positioning system (GPS) tagged 2D orthomosaics and 3D coloured point clouds. We installed ground control points (GCPs) and height reference points to remove unwanted terrain features (e.g. slopes). Geo‐coordinates of the 2D/3D imagery derived by the drone phenotyping were calibrated after every flight.

For LiDAR‐based mapping, backpack LiDAR operators routinely walked around the perimeter of each column of pear trees (red arrows in Figure 1a) to conduct phenotyping from different perspectives. Operators normally performed the mapping at a walking speed of 4–5 km·h^−1^ (i.e. a mapping speed of 0.8–1 ha·h^−1^). Similar to the drone phenotyping, LiDAR‐based mapping was conducted at the bud burst and flowering stages. Using RTK information collected by a ground‐based RTK station (Hisinda IRTK2, Hisinda Surveying and Mapping Instrument, Guangzhou, China), accurate geo‐positions were logged for objects such as the reference points and tree trunks, with an error range of ±5 mm. The LiDAR used in the United Kindom scanned the orchard at 3.5 km·h^−1^. RTK‐GNSS information was provided using REACH RS2 (Emlid, Budapest, Hungry), which helped us precisely geo‐position fruit trees using the LiDAR‐bundled software (Note S2) and tag 3D point clouds with mm‐level geo‐coordinates.

### Pre‐processing of drone‐ and LiDAR‐collected 3D point clouds

Before performing any meaningful trait analysis, we pre‐processed drone‐derived and LiDAR‐scanned 3D point clouds. Red‐coloured outlier points (i.e. noise signals; Figure 1b, upper) were removed using the Statistical Outlier Removal (SOR) algorithm (Hodge and Austin, 2004). Then, a ground‐level filter approach was applied based on the Cloth Simulation Filter (CSF) algorithm (Zhang *et al*., 2016), dividing denoised 3D points into ground level (i.e. digital terrain model, DTM) and aboveground (i.e. digital surface model, DSM, without terrain features) groups. Finally, within the region of interest (ROI; Figure 1a), DTM was subtracted from the DSM to retain tree‐level spatial properties (Figure 1b, lower) with a canopy height model (CHM). CHM and spatial information were pseudo‐coloured according to a unified height scale bar (0–300 cm) that is used throughout this study. When carrying out software implementation, input parameters were adopted from the AirMeasurer platform (Sun *et al*., 2022).

### An automated approach for tree segmentation

Utilising the CHM produced at the bud burst stage, we established an automated analysis pipeline to segment trees in the pear orchard (Figure 1c), including (1) a 2D projection of the CHM from an overhead perspective, which used greyscale values (0–255) to present spatial signals (i.e. the brighter a pixel, the higher the 3D point); (2) using Tree 18 (row 2 and column 9 in the orchard) as an exemplar, we combined the Otsu Local Auto Threshold (Otsu, 1979) and morphological open (Bhutada *et al*., 2022) algorithms to identify tree‐level ROI signified by a red‐coloured bounding box; (3) GNSS‐tagged tree masks were then applied to the CHM, enabling the segmentation of 3D pear trees, with overlapping branches or canopy edges removed.

Building on the pear tree segmentation, we applied the above pipeline to enable rapid and large‐scale segmentation of thousands of trees in the apple orchard, including (1) obtaining the coordinates of the centroids for the starting and ending trees of each row in the orchard; (2) calculating GNSS information of every tree trunk based on its geo‐positions in a row assisted with linear interpolation if gaps or dead trees were encountered; (3) building a 3D bounding box for every tree to define tree boundaries; and (4) performing scalable tree segmentation using the defined polygon boundaries algorithm (Lindsay, 2016). Source codes and software implementation guidelines of the above apple and pear pipelines are given (see Open Access).

### Position trees using the multi‐source 3D point clouds

**Figure 2:**
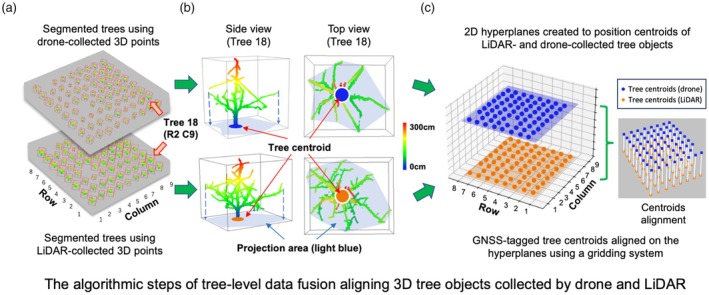
The algorithmic steps of tree‐level positioning to align tree centroids collected by drone and LiDAR with hyperplanes and a gridding system. (a) Segmented trees with 3D bounding boxes based on drone‐derived and LiDAR‐collected 3D point clouds. (b) Positioning pear trees using trunk 3D points and the overhead projection. (c) The creation of a gridding system to place drone‐derived and LiDAR‐based tree centroids so that tree centroids detected from both approaches were integrated with GNSS information.

After segmenting trees, we combined the drone‐derived and LiDAR‐collected 3D point clouds to position tree trunks with a mm‐level accuracy (Figure 2a). The geo‐coordinate system used by the drone was the China Geodetic Coordinate System (Yang, 2009), whereas the LiDAR was based on the Geocentric Datum of Australia (Steed and Luton, 2000). Hence, we developed a hyperplane‐based approach to combine the two 3D points tagged with different geo‐coordinates. Still using Tree 18 to visualise, the algorithmic steps for positioning trees include (1) the definition of a 2D affine hyperplane in the 3D Cartesian coordinate system (i.e. a 3D bounding box), followed by the overhead projection of 10% of the bottommost 3D points (i.e. tree trunk) to the hyperplane to produce the weighted tree centroid (blue for drone and orange for LiDAR; Figure 2b, left); (2) the generation of a 2D convex hull based on the overhead projection of 3D points of the tree (light blue; Figure 2b, right), representing the canopy projection area; (3) the weighted tree centroids identified in the drone‐ and LiDAR‐based hyperplanes were aligned and placed at the centre of grids in a new and unified gridding system (Figure 2c) as trees were not perfectly aligned in the orchard; and (4) finally, gaps and dead trees in the grids were identified (Note S3), followed by mm‐accurate geo‐coordinates collected by the LiDAR associating with tree centroids to enable tree‐level positioning in the orchard (Note S4).

### Data fusion to combine multi‐source 3D point clouds

**Figure 3:**
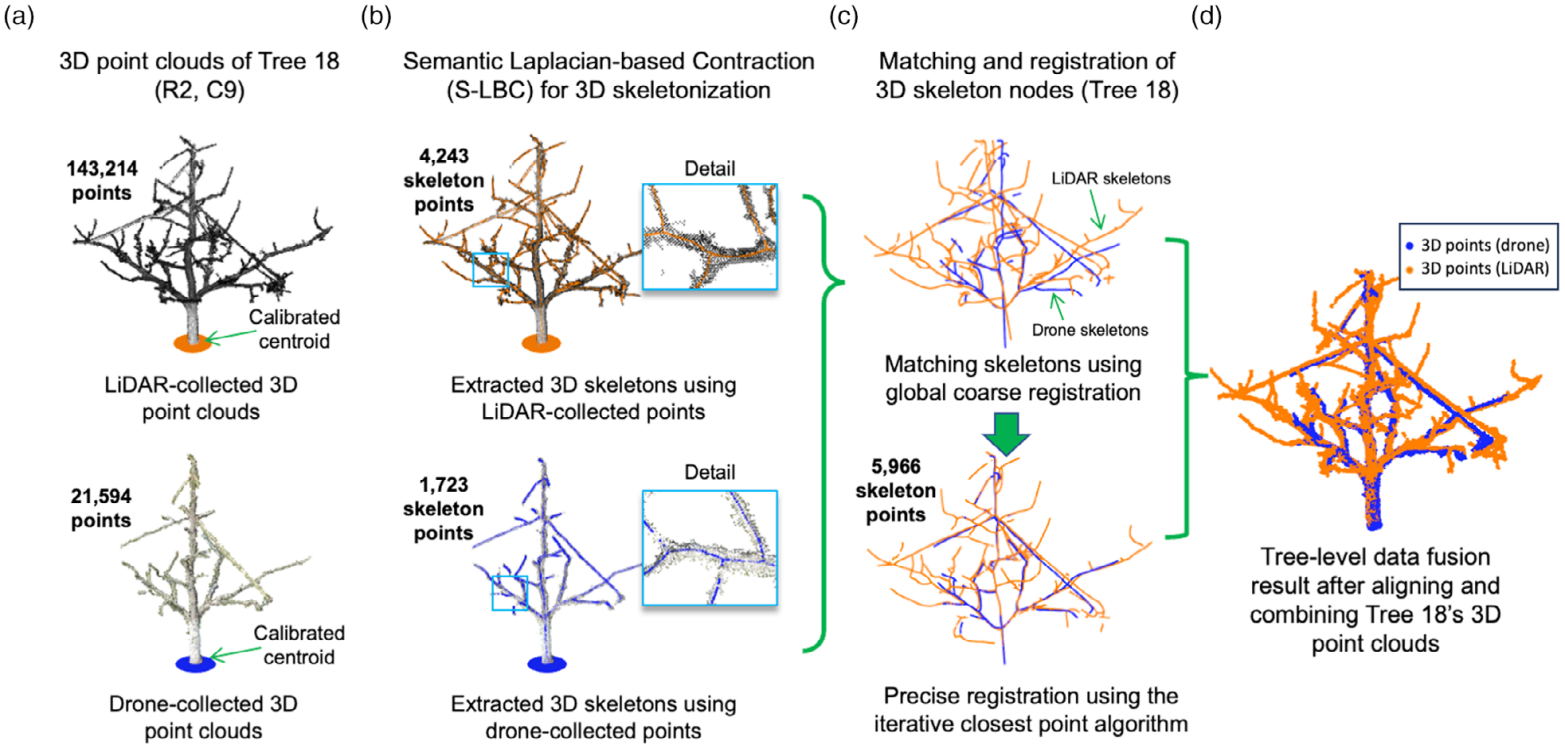
The data fusion approach to combine drone‐derived and LiDAR‐collected 3D point clouds based on tree‐level 3D skeletons at the bud burst stage. (a) The display of tree‐level 3D point clouds for Tree 18 collected by the LiDAR and drone approaches. (b) Semantic Laplacian‐base Contraction (S‐LBC) algorithm applied to extract 3D skeletons from tree‐level 3D points. (c, d) Match and register drone‐derived and LiDAR‐based tree skeletons using both global coarse and iterative precise registrations, so that a refined tree‐level 3D point clouds could be produced for phenotypic analysis.

Besides fusing multi‐source data to precisely position trees, tree‐level morphological, spatial and colour signals acquired by the two approaches were also combined for tree‐level morphological and trait analysis. Due to different mapping mechanisms (i.e. SfM and laser scanning), 3D points collected by the two approaches have spatial and structural differences. For example, two 3D point clouds acquired from Tree 18 varied in terms of the total number of 3D points, point cloud density and structural features (Figure 3a), which caused data fusion issues when comparing 3D points acquired at different growth stages and from different mapping methods. Hence, we applied the semantic Laplacian‐based contraction (S‐LBC) algorithm (Meyer *et al*., 2023) to generate of 4243 LiDAR‐collected (coloured orange) and 1723 drone‐derived (coloured blue) 3D skeleton points (Figure 3b).

Then, we performed a two‐step tree registration using the two sets of 3D skeleton points: (1) coarse global registration, which was powered by the Fast Global Registration algorithm (Zhou *et al*., 2016) to carry out global registration based on fast point feature histogram (Rusu *et al*., 2009), followed by an iterative fast feature transformation (up to 50 iterations) to obtain a refined tree skeleton when the smallest root mean square error (RMSE) was reached (Figure 3c, upper); (2) fine local registration, which employed the iterative closest points algorithm (Besl and McKay, 1992) to perform precise skeleton registration, resulting in an optimised tree skeleton (Figure 3c, lower). The above steps not only helped us automatically register multi‐source 3D skeletons for different tree varieties (Note S5) so that 3D points collected by the two mapping methods could be combined (Figure 3d), but also facilitated us to precisely associate 3D points acquired at different growth stages (e.g. LiDAR‐derived tree skeletons during bud burst for morphological analysis and drone‐derived 3D colour point clouds during blossom for blossom‐related analysis), for multi‐source trait analysis. Notably, the above method has reduced computational complexity dramatically as much less feature points (i.e. 3D skeleton points) were required to compare when performing tree registration and data fusion.

### Pruning the 3D tree skeletons

**Figure 4:**
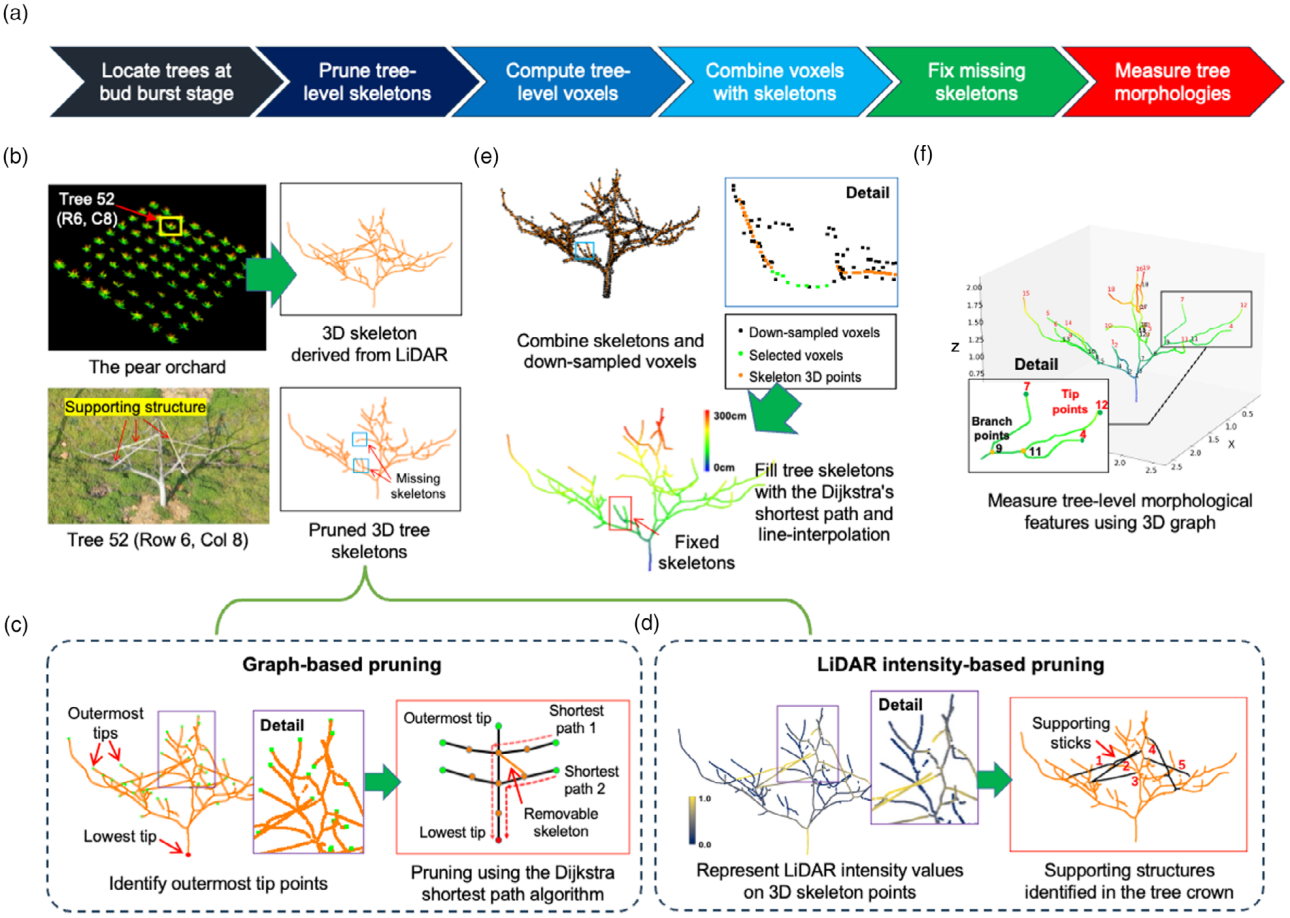
The improvement of tree‐level 3D skeletons after removing supporting structures for canopy‐level morphological analysis at the bud burst stage. (a) A tailor pruning pipeline developed to largely remove supporting structures based on 3D graph theory and LiDAR intensity values for canopy‐level analysis. (b) The display of Tree 52 together with its 3D skeletons. (c) A 3D‐graph‐theory‐based approach to remove unwanted supporting structures. (d) A LiDAR intensity‐based method to remove unwanted supporting structures. (e) Downsampled voxels to fill the breaking skeletons, resulting an improved 3D skeletons using 3D points smoothing and line interpolation. (f) The quantification of 3D morphological traits using the refined skeletons, including branch points and the number of branches.

Before performing morphological analysis of fruit trees, we still need to further refine tree skeletons due to supporting structures and missing skeletons caused by branch occlusion and mapping methods. Orchards commonly include supporting structures for individual trees (e.g. branch, post and wire, or trellis supports) to obtain desired tree canopy structures such as a suitable crown size and branch spacing, optimising sunlight exposure and improving air circulation for better fruit yield and quality (Liu *et al*., 2018). Thus, based on the LiDAR‐derived 3D skeletons (Figure 3c), we further developed a tailored pruning algorithm to remove unwanted supports from the tree skeletons (Figure 4a).

Using Tree 52 (row 6, column 8 in the orchard) with both supports and branch occlusion presented (Figure 4b), manual removal of supporting structures (indicated by red‐coloured arrows in Figure 4b, lower left) could be extremely time‐consuming and prone‐to‐error. Hence, we used both 3D graph theory and LiDAR intensity values, that is, the return strength of the laser pulse (Song *et al*., 2002), to automate the task. For the 3D graph‐based pruning method (Figure 4c), we first used the LiDAR‐derived 3D skeletons of Tree 52 obtained at the bud burst stage (Figure 4b; upper right) to generate an undirected 3D graph $G\left(V,E\right)$, consisting of orange‐coloured nodes (i.e. vertices, *V*) and links connecting the nodes (i.e. edges, *E*). This step was accomplished by performing the Radius‐based Nearest Neighbour (R‐NN) search (Karger and Ruhl, 2002; Zhou *et al*., 2018) using a KDTree structure, that is, a space‐partitioning data structure for organising points (Zhou *et al*., 2008), which identified points within a specified radius from a given 3D point, with radius automatically computed according to the diameter of every tree trunk. Then, we applied the square of Euclidean distance to identify outermost edge points (i.e. tree branch tips; coloured light green) between two given nodes and the lowest point, that is, the trunk base, located with the lowest z‐axis value of the 3D graph (coloured red; Figure 4c, left). Finally, we employed the Dijkstra's shortest path algorithm (Shekhar and Xiong, 2003) to detect shortest paths between the outermost edge points and the lowest point, eliminating 3D skeletons that were not relatively vertically positioned between tree trunks and branches (Figure 4c, right).

As the graph‐based pruning that might not be able to fully remove supporting structures, a LiDAR intensity‐based pruning method was also developed as supporting structures and tree branches were normally made of different materials and thus dissimilar laser reflectivity values. This approach (Figure 4d) used the radius of every tree trunk as the parameter to perform the R‐NN algorithm, which assigned normalised LiDAR intensity values (Note S6) to all the skeleton points (Figure 4d, left; values were pseudo‐coloured according to a 0 ~ 1 scale bar). Then, intensity values of all the skeleton points were compared with the average tree‐level reflectivity value, identifying supporting structures with higher intensity values in the crown (yellow‐coloured; Figure 4d, left). To demonstrate the generalisation of the above algorithmic steps, we chose three pear trees with diverse morphological features and performed the structure removal task with either the graph‐based or the LiDAR intensity‐based approach, which resulted in satisfactory outcomes for different types of tree morphologies (Note S6).

Nevertheless, due to branch occlusion and potential over removal of supporting structures, the pruned tree skeletons could contain gaps (i.e. missing skeletons; Figure 4b, lower right). Hence, we performed voxel downsampling (Chen *et al*., 2023; Zou *et al*., 2020) on the original LiDAR‐collected 3D points and overlapped the voxels with the pruned skeletons (Figure 4e, upper left). Then, the Euclidean distance clustering algorithm (Aloise *et al*., 2009) was applied to identify missing skeletons, followed by the application of the Dijkstra's algorithm to find shortest paths from breaking skeletons to the lowest trunk base, so that the downsampled voxels could fill the missing skeletons (green‐coloured points; Figure 4e, upper right). Notably, a precise voxel screening was followed to ensure that 3D points from the removed supporting structures were not reintroduced (Note S6). Finally, we combined 3D points smoothing (Landrieu *et al*., 2017) with line interpolation (Mao *et al*., 2019) to finalise the task (Figure 4e, below), resulting in a refined tree skeleton (Figure 4f) that was suitable for precisely quantifying morphological traits such as branch points and the number of tree branches (Num_Branch_; Note S7).

### 3D morphological analysis using point clouds at the bud burst stage

Using the refined tree skeletons, we recovered associated LiDAR‐collected 3D points using their related transformation matrices (Mémoli and Sapiro, 2004) for morphological analysis at the tree level. For canopy‐related traits, we first identified crown region using the density‐based spatial clustering of applications with noise (DBSCAN) algorithm (Ferrara *et al*., 2018), enabling the calculation of the number of 3D points between the bottom of a tree trunk (i.e. lowest tip; Figure 4c, left) and the tallest tip of a trunk. 3D points above the tallest tip of a trunk were treated as the crown region. Then, we measured a range of morphological traits using LiDAR‐collected 3D points at the bud burst stage, including maximum tree height (Height_Max_), crown projection area (ProjArea_Crown_), crown volume (Volume_Crown_) and surface area (SurfArea_Crown_), and crown diameter (Diameter_Crown_):

1. Height_Max_ was computed based on the difference between the highest point in the crown and the lowest point in the trunk after sorting all 3D points according to their z‐axis values (i.e. height).
2. ProjArea_Crown_ was quantified using the overhead projection of all 3D points of a given tree onto a ground‐based plane, followed by the computation of the 2D convex hull polygon area enclosing the projection area (Virtanen *et al*., 2020) to obtain this trait (Figure 2b).
3. Both Volume_Crown_ and SurfArea_Crown_ were obtained through forming a 3D convex hull (Zhou *et al*., 2018) of the tree crown, followed by the calculation of the surface area and volume of the convex hull (Sullivan and Kaszynski, 2019).
4. Diameter_Crown_ was quantified using the maximum values of a tree crown's most east–west and north–south points. The maximum length of every pear tree was calculated as the trait.

### 3D floral trait analysis using point clouds at the bloom stage

**Figure 5:**
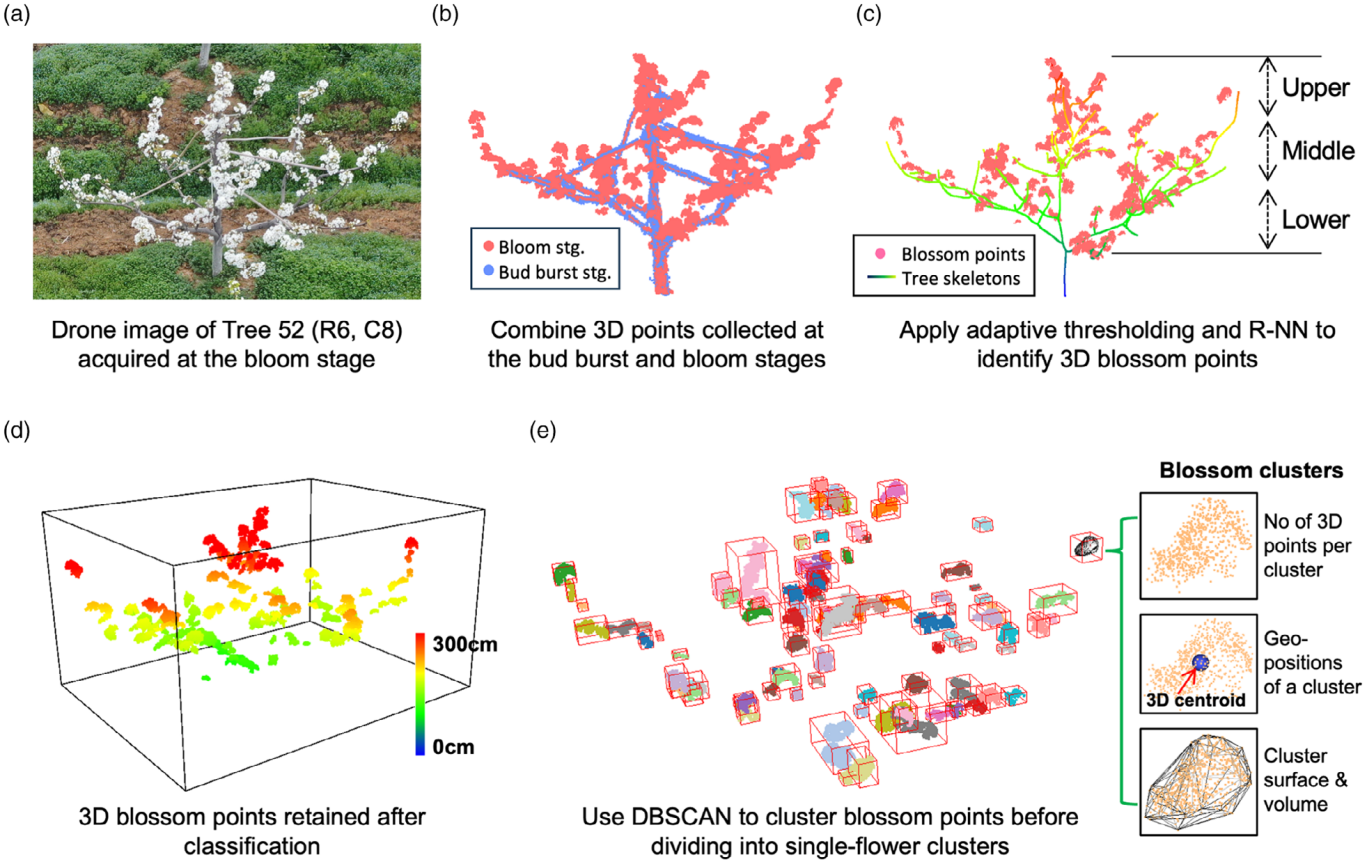
3D floral analysis using refined tree‐level 3D points at the bud burst stage and 3D blossom points collected at the bloom stage. (a) A bloom stage image of Tree 52. (b) Combine 3D points acquired at the bloom and bud burst stages. (c, d) Apply adaptive thresholding and R‐NN removal method to retain 3D blossom points with spatial information. (e) Apply DBSAN to produce 3D blossom clusters, followed by the use of a K‐Means classifier to obtain single‐flower groups from the blossom clusters; a range of tree‐level key floral traits was measured, including the number of 3D blossom points, the number of blossom clusters, positions of blossom clusters, blossom cluster volume and surface.

Using Tree 52 during flowering for visualisation (Figure 5a), we combined both LiDAR‐derived tree skeletons at bud burst stage (Figure 4f) and drone‐derived 3D colour points at the bloom stage (Figure 5b) to perform tree‐ and branch‐level floral analysis. As RGB values of pear flowers (or apple flowers) were very different compared with other tree‐level organs or structures (e.g. leaves and branches), we first converted RGB channels to greyscale images and then applied an adaptive global threshold algorithm (Meyer and Neto, 2008) to largely remove non‐blossom 3D points. Still, as some 3D points could not be removed due to high intensity values caused by natural illuminance, we employed the R‐NN search to remove neighbouring points based on the LiDAR‐derived bud burst skeletons (Figure 5c), with the scanning radius set as the radius of the tree trunk (*r* = *R*_*Trunk*_). Then, the adaptive thresholding and R‐NN removal method were combined, helping us retain 3D blossom points tagged with GNSS‐tagged information, as well as divide blossom points into upper, middle and lower groups according to their vertical positions in the crown (Figure 5c,d).

After obtaining blossom groups, we then employed the DBSCAN algorithm to group 3D blossom points into clusters, with neighbouring radius (*r* = *R*_*Trunk*_) and the minimum number of 3D points per cluster (*n* ≥ 20, derived by specialists) pre‐configured. An unsupervised K‐means classifier (Likas *et al*., 2003) was employed to divide blossom clusters into three groups (Figure 5e): (1) noise (*n* < 50 in a given cluster), (2) single‐flower and (3) multiple flowers. For multi‐flower groups, we iteratively divided the blossom clusters until no multi‐flower group could be found for the tree. Finally, all the single‐flower groups were triangulated, based on which traits such as the number of 3D points per cluster, 3D centroid of a cluster, surface area (Surf_Blossom_) and volume per cluster (Volume_Blossom_) were quantified using voxelization, 3D convex hull and topological assessment. For apple blossom clusters, same steps were followed to measure key floral traits.

### Software implementation and availability

When implementing the OrchardQuant‐3D pipeline, we followed the modular design by breaking source codes into modules with specific functions to promote better reusability and easier debugging. We used Python (V3.8) (Millman and Aivazis, 2011) as the main programming language together with a range of 2D and 3D CV and ML libraries and frameworks, including OpenCV (4.8.1) (Howse, 2013), Open3D (0.18.0) (Zhou *et al*., 2018), Whitebox (2.3.1) (Lindsay, 2016), VTK (9.2.6) (Sullivan and Kaszynski, 2019) and SciPy (1.9.1) (Virtanen *et al*., 2020). To automate tree‐level canopy and floral trait analysis for different sizes of orchards, we applied adaptive parameterisation to derive parameters for algorithms embedded in the pipeline to reduce hardcoded values. For example, the distance parameterisation was based on measures of tree trunks and reference points (e.g. GCPs) in the orchard, whereas colour (e.g. RGB values) or LiDAR intensity values were normalised according to unified scales followed across key growth stages. By doing so, we were able to derive most of the input parameters for the pipeline automatically (Note S8). For several parameters and experiential constants that were impossible to automate, plant specialists were involved in setting values to achieve optimal outcomes of phenotypic analysis (Note S8).

In order to promote open research, we chose to use software libraries that are openly accessible. Besides the above 2D/3D CV and ML libraries, we also employed: (1) NumPy (1.23.2), Pandas (2.0.3), GeoPandas (0.13.2), Dijkstra (0.2.1) and PyShp (2.3.1) for scientific data processing; (2) Scikit‐Image (0.20.0), Rasterio (1.3.9) and GDAL (3.6.2) for 2D image analysis; (3) Matplotlib (3.7.3) for result visualisation; (3) scikit‐learn (1.3.2) (Pedregosa *et al*., 2011) for building classifiers; (4) laspy (2.5.1), CSF (1.1.5) and pc‐skeletor (1.0.0) for 3D point clouds processing and 3D trait analysis. Furthermore, to ensure that our work could reach broader researchers, we created a graphical user interface (GUI) for key steps of the OrchardQuant‐3D pipeline (i.e. tree segmentation, 3D tree skeletonization, canopy‐ and branch‐level trait analysis), so that nonexperts can easily utilise our work (Note S9). We employed the Tkinter toolkit (Shipman, 2013) to develop the cross‐platform GUI software.

When conducting software development and testing, a Windows 10 workstation (NVIDIA 1080 GPU, Intel® Core™ i7‐8700 CPU and 64 GB RAM) was used. Benchmark timing recorded for pear orchard analysis was around 80–90 s per tree (with 60–65 s computational time spent on calculating tree branches). To enable the research community to reproduce our work, source codes of the OrchardQuant‐3D pipeline, embedded modules and trained DL models have been uploaded to our GitHub repository in executable Jupyter notebook files together with testing files (in .LAS and RTK shapefile formats), runtime and configuration guides (see Open Access).

### Ground‐truthing and evaluation metrics

For the pear orchard in China, specialists manually scored several morphological traits from the 70 trees, including maximum height (with a ruler, from the tip of the canopy to the lowest point of the trunk), tree‐level branch numbers (Num_Branch_) and branch‐level blossom clusters (Num_Blossom_; measured from eight trees randomly selected). For Volume_Crown_ and canopy or floral traits (e.g. SurfArea_Blossom_) that were impractical to measure, CloudCompare (Girardeau‐Montaut, 2015) was used to manually measure these traits based on the original 3D point clouds. The above measures were carried out at least three times at key growth stages, so that the mean value could be used to minimise errors during manual scoring. For the apple orchard in the United Kingdom, a team of field specialists conducted manual scoring of traits such as tree height and total blossom clusters on individual trees 5 days before the drone phenotyping (e.g. 11/04/2024 and 17/04/2024). Ninety‐five trees were randomly selected from the 1104 trees for manual assessment. Similar to the pear study, manual scores were averaged and then used to verify computationally derived traits. This study also employed the coefficient of determination (R^2^), root mean square error (RMSE) and *p*‐value to validate the accuracy of the computationally derived traits against manual scoring using the IBM SPSS Statistics 23 software (George and Mallery, 2019).

## Results

### Tree‐level analysis of canopy and blossom traits

**Figure 6:**
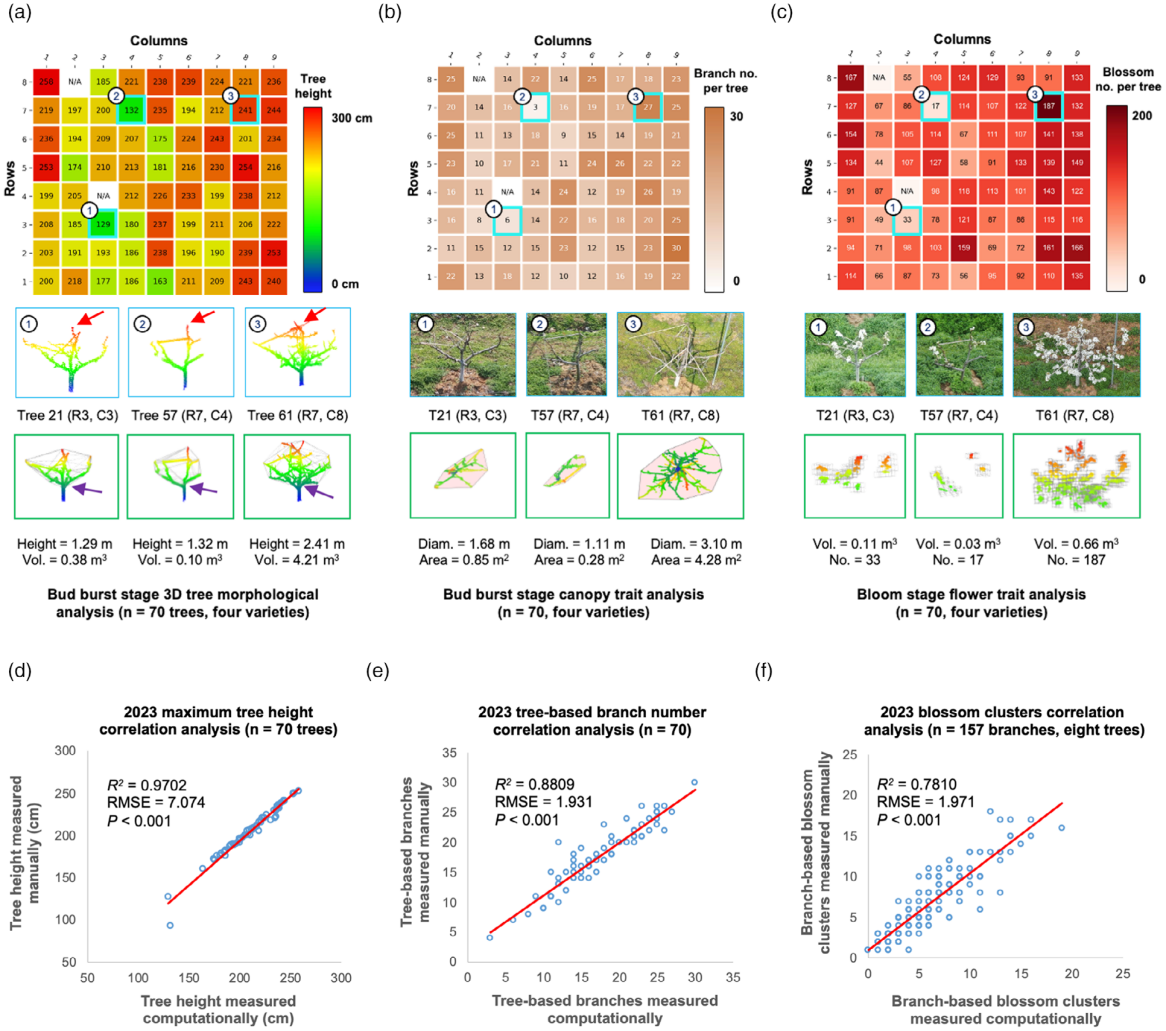
Apply the OrchardQuant‐3D method to analyse 70 pear trees in the orchard, showing both morphological and blossom‐related phenotypic differences between individual trees at the bud burst and flowering stages. (a) A pseudo‐coloured maximum tree height map with a height scale bar (0–300 cm), showing tree‐level maximum height values derived by the OrchardQuant‐3D method at the bud burst stage. Three exemplars (Trees 21, 57 and 61) selected for visual display due to their different canopy and floral characteristics. 3D point clouds (from a side perspective) and crown region convex hulls of the three exemplars are also given with measurements, demonstrating their phenotypic differences. (b, c) Similarly, a tree‐level branch number map and a tree‐level blossom cluster number map were generated together with two pseudo‐coloured scale bars (0–30 branches and 0–200 blossom clusters, respectively), showing tree‐level branch (at the bud burst stage) and blossom cluster values (at the bloom stage) computed by the OrchardQuant‐3D method. Photographs of the three exemplars acquired at the two stages, visualisation for computing canopy projection areas and blossom cluster volumes are also provided together with trait analyses, including crown diameter, canopy projection area, blossom cluster volume and the number of blossom clusters. These helped us demonstrate the phenotypic differences of pear trees using key canopy and floral traits. (d–f) Coefficient of determination (*R* ^2^) and root mean square error (RMSE) computed to evaluate correlations between OrchardQuant‐3D derived and manually scored maximum tree height, tree‐level branch number and branch‐level blossom clusters, showing a high degree of accuracy of computationally estimated traits with significant *p‐*values (*p* < 0.001).

The OrchardQuant‐3D was applied to perform phenotypic analysis for different sizes of pear and apple orchards. For example, we computed a range of tree‐level traits (e.g. Height_Max_ and Num_branch_), canopy‐level morphological features (e.g. Volume_Crown_, SurfArea_Crown_, Diameter_Crown_ and ProjArea_Crown_) and key floral traits (e.g. Num_Blossom_, Volume_Blossom_, SurfArea_Blossom_ and ProjArea_Blossom_) in the pear orchard. For visual display, we produced a pseudo‐coloured tree height map (0–300 cm; Figure 6a, upper) to demonstrate computationally derived Height_Max_ values for the 70 trees, showing height differences between the pear trees at the bud burst stage. Besides tree height, other canopy‐level traits such as Volume_Crown_ (m^3^) and SurfArea_Crown_ (m^2^) were measured based on 3D convex hulls derived from 3D point clouds in the crown region, with or without supporting structures (Note S10). The crown region was defined between the topmost of crowns (pointed with red arrows) and the top of tree trunks (pointed with purple‐coloured arrows), showing different crown sizes between the cultivars (Figure 6a). To further demonstrate phenotypic differences, three exemplars of pear trees were selected from the orchard (i.e. Tree 21, Tree 57 and Tree 61) together with 3D tree structures, crown volumes and the quantification of key morphological traits (Figure 6a, lower). Tree‐level measures of crown volume (Table S1) and surface area in the pear orchard (Table S2) are provided.

To further quantify varietal differences of the four varieties, we computed traits such as tree‐level Num_Branch_, ProjArea_Crown_ (m^2^) and Diameter_Crown_ (m) at the bud burst stage, among which the Num_Branch_ trait was presented by a pseudo‐coloured map according to a scale bar (0–30 branches per pear tree; Figure 6b, upper). Bud burst stage photographs of the three exemplars were given together with their overhead 3D projection images (Figure 6b, lower), and measures of the Diameter_Crown_ (Table S3) and ProjArea_Crown_ traits (Table S4). Besides canopy‐level traits, key blossom‐related features such as tree‐level Num_Blossom_ and SurfArea_Blossom_ were analysed, including an orchard‐level blossom cluster map (0–200 clusters per tree; Figure 6c, upper), blossom photographs, 3D bounding boxes of blossom voxels (Figure 6c, lower) and the quantification of Num_Blossom_, Volume_Blossom_, SurfArea_Blossom_ and ProjArea_Blossom_ (Tables S5–S8).

### Evaluation of OrchardQuant‐3D‐derived traits

While we could not manually evaluate all the computationally derived traits listed above, the accuracy of several key traits was examined against manually scored values taken from the 70 pear trees. We employed both *R* ^2^ and RMSE to perform the following evaluation: (1) tree height was estimated to a very high degree of accuracy (R^2^ = 0.97, RMSE = 7.074 cm per tree; Figure 6d); (2) the estimation of branch number (*R* ^2^ = 0.88, RMSE = 1.931 branches per tree; Figure 6e) and the number of blossom clusters (*R* ^2^ = 0.78, RMSE = 1.971 clusters per branch; Figure 6f) suggested slightly less accuracy; and (3) *P‐*values for the above regression analyses were all significant (*P* < 0.001).

Besides comparing key OrchardQuant‐3D‐derived traits against manual scoring, we also compared our pipeline with three representative methods, TreeQSM, AdTree and AppleQSM, for measuring the Num_Branch_ trait. To ensure meaningful comparisons, we used 3D points of Tree 38 with supporting structures removed (45 branches according to manual scoring), as well as default parameters published with the three methods (Note S11). The TreeQSM (28 branches quantified; Figure S11.1), AdTree (5494 branches due to many computationally generated tiny pseudo‐branches) and AppleQSM (eight branches due to different crown structures between apples and pears; Figure S11.2) methods produced various results, whereas the OrchardQuant‐3D method identified 42 branches from Tree 38 (with three small branches missing; Figure S11.3). Additionally, we explored varietal differences using 12 canopy and floral traits by performing one‐way ANOVA, which helped us determine whether significant differences could be found between the four pear varieties based on these traits (Note S12).

### In‐depth analysis of canopy and floral traits to assess varietal differences

**Figure 7:**
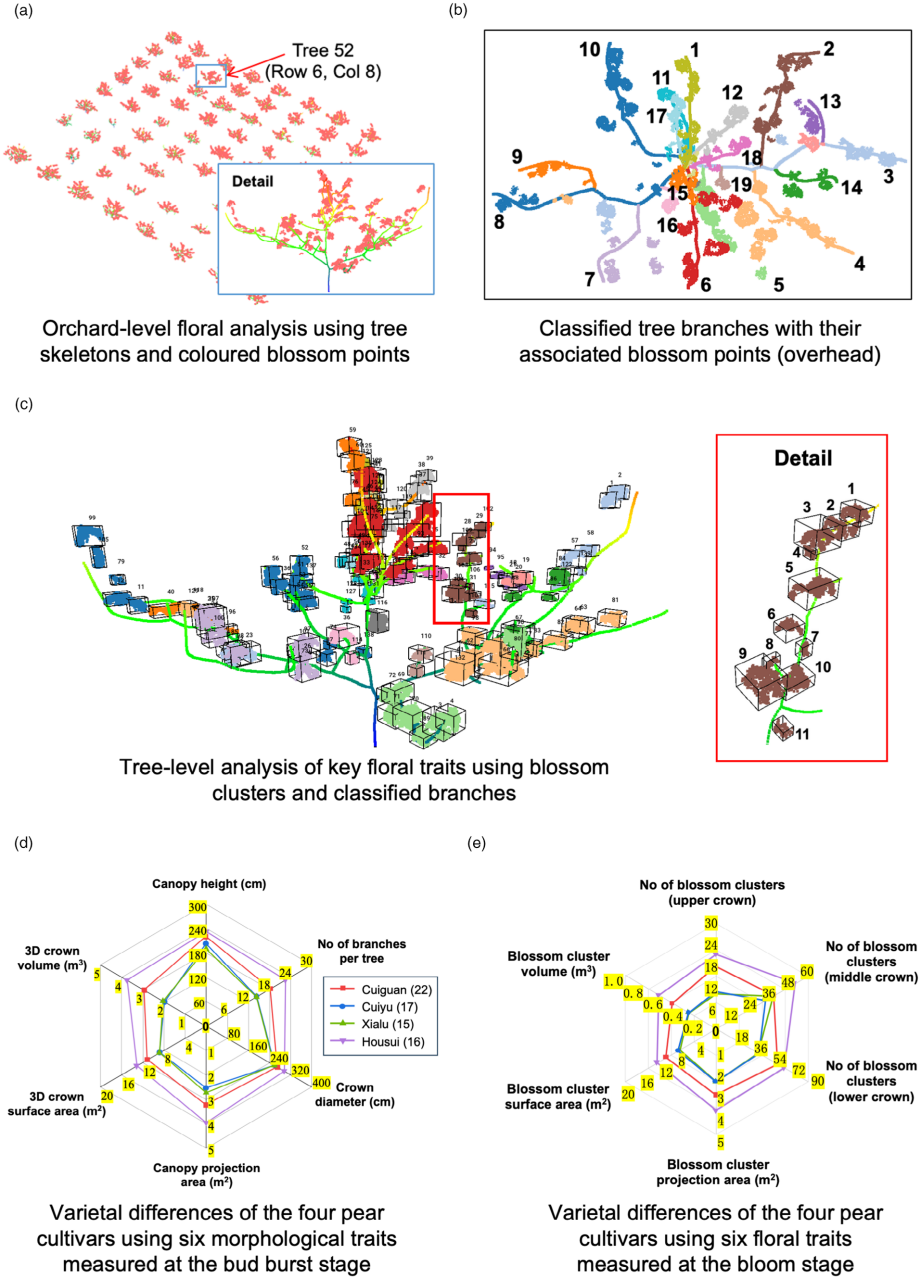
Using the OrchardQuant‐3D method to analyse key floral traits of the 70 trees in the pear orchard, based on which varietal differences between the four cultivars at the bud burst and bloom stages were measured using radar diagrams. (a) Combine bud burst 3D tree skeletons and bloom‐phase 3D blossom points to represent varietal differences between the 70 trees. An exemplar (i.e. Tree 52) was used for visual display. (b) Use classified tree branches to group 3D blossom points at the tree level, obtaining branch‐level blossom features. (c) Tree‐ and branch‐level analysis of key floral traits including the number of blossom clusters, cluster‐based volume, surface area and positions. (d, e) Radar diagrams used to show varietal differences of four pear cultivars at the bud burst and blossom stages using six morphological and six blossom‐related traits.

When performing detailed analysis of key floral traits to assess differences in the four pear varieties, we first combined the LiDAR‐collected bud‐stage 3D tree skeletons (without supporting structures) with drone‐collected bloom‐stage 3D colour points (Figure 7a). Using Tree 52 for visualisation, beside tree‐level flower characteristics, detailed branch‐level assessment of blossom‐related features was also enabled, with blossom clusters on every branch numbered and pseudo‐coloured (Figure 7b). This combined approach facilitated us in measuring key floral traits such as branch‐level Num_Blossom_, Volume_Blossom_, SurfArea_Blossom_, ProjArea_Blossom_ and every blossom cluster's geo‐coordinates, all of which were impractical to score previously (Figure 7c). Branch‐level analysis results of key floral traits (brown coloured in Figure 7c) in the pear orchard are provided in Table S9.

Building on 3D mapping of canopy and floral traits, we then assessed varietal differences of the four pear cultivars according to their crown and blossom characteristics. Two radar diagrams were produced to incorporate both canopy morphological and floral differences: (1) during bud burst, a radar diagram generated using six tree‐level crown‐related traits (Figure 7d), indicating that Xialu and Cuiyu were morphologically very similar, whereas Cuiguan and Housui possessed more branches and bigger crowns, and Housui variety's crown volume and surface area were the biggest among the four varieties; (2) during blossom, a floral‐feature radar diagram was created, which suggested that Housui had the largest values for blossom‐related features (e.g. number of clusters and 3D blossom volumes), followed by Cuiguan (17% less on average), with Cuiyu and Xialu sharing similar features except for the Num_Blossom_ trait in the middle crown (Figure 7e).

### The use of OrchardQuant‐3D in a large‐scale apple orchard study

**Figure 8:**
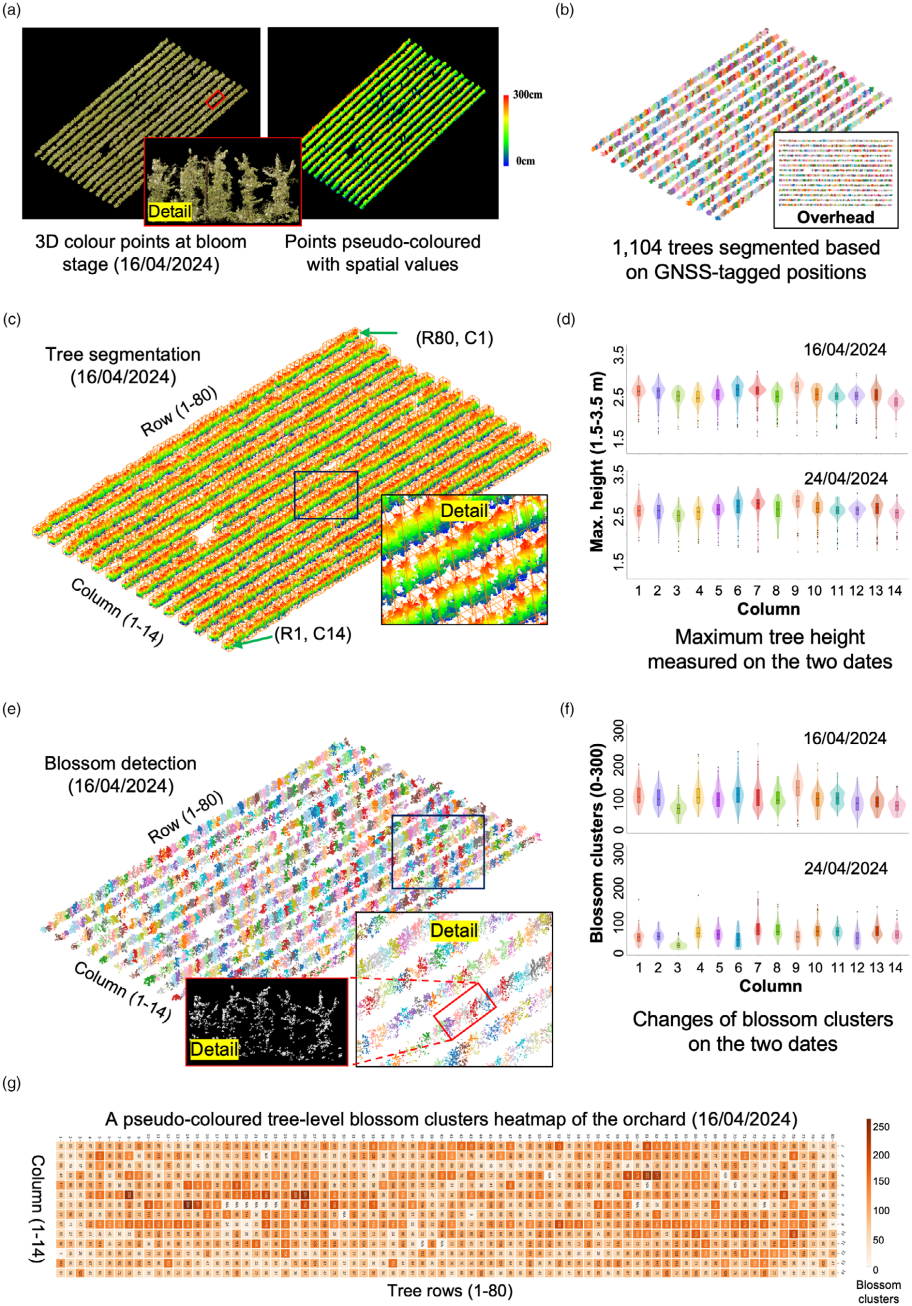
Apply the OrchardQuant‐3D method to study a large‐scale apple orchard with 1104 trees during flowering. (a) 3D coloured point clouds collected by drones and point clouds pseudo‐coloured with spatial signals on 16/04/2024. (b) A total of 1104 coloured apple tree objects in the orchard that were segmented using the OrchardQuant‐3D method. (c) 3D bounding boxes applied to tree objects to demonstrate a clear and non‐overlapping tree boundary. (d) Two violin diagrams created to compare column‐based maximum tree height values quantified computationally on two dates, 16/04/2024 and 24/04/2024. (e) Tree‐level blossom points extracted from the drone‐derived point clouds, which were used to compute key floral traits. (f) The comparison of column‐based average blossom clusters per tree on 16/04/2024 and 24/04/2024. (g) A pseudo‐coloured heat map produced to show tree‐level blossom clusters of the apple orchard on 16/04/2024.

The OrchardQuant‐3D method is not only applicable to analyse trees in small‐sized fruit orchards, but also scalable to provide automated analysis in large‐scale orchards. We applied the pipeline to analyse an apple orchard with 1104 trees at NIAB's East Malling site (Kent, England). At the bud burst stage, we used a tractor‐mounted LiDAR to acquire medium‐density 3D points to help position trees with GNSS. Then, drone 3D mappings were performed on between 16/04/2024 (when trees were approaching the peak blossom date) and 24/04/2024 (i.e. petal fall). After that, we produced 3D colour point clouds of the apple orchard with GNSS signals (Figure 8a). The large‐scale tree segmentation resulted in coloured tree objects (Figure 8b), indicating a clean and non‐overlapping tree segmentation. 3D bounding boxes for segmented trees are also visualised (Figure 8c).

The tree‐level segmentation empowered us to perform analysis for key canopy and floral traits at the tree, row/column and orchard levels. We used violin diagrams to present the data distribution of the Height_Max_ trait on both 16/04/2024 and 24/04/2024 (Figure 8d), showing column‐based average height values between the two dates were very similar. The majority of the trees were 2.5–3.0 m in height, with trees in Column 3 and Column 14 relatively shorter than those in other columns. Several exceptional cases (i.e. outliers) were observable in the violine diagrams, which were caused by terrain features, dissimilar canopy growth at the edges of the orchard, dead trees and tree gaps. We chose to perform the correlation analysis using zone‐based height values (5 columns and 3 rows per zone, height values of 15 trees in the zone were averaged) between the two dates, resulting in *R* ^2^ = 0.847 (RMSE = 0.27, *p* < 0.001), indicating a highly significant positive correlation of the computational scored Height_Max_ trait on the two dates. Canopy‐level morphological analyses such as tree‐level Volume_Crown_ on the two dates are also provided (Tables S10 and S11).

Similarly, we examined key floral traits at the two dates. 3D coloured blossom points were first measured to derive tree‐level blossom clusters by the OrchardQuant‐3D (Figure 8e). Then, two violin diagrams were produced to compare column‐wise blossom clusters (Tables S12 and S13), indicating that apple blossoms dropped noticeably within the 8 days, with 30%–70% decreases across the columns (Figure 8f). This was also verified by in‐field observation as the peak blossom occurred around 17/04/2024. Then, we conducted the correlation analysis of tree‐level blossom clusters (16/04/2024) against manual scoring (17/04/2024), achieving a positive correlation (R^2^ = 0.649; normalised RMSE = 0.160 due to slightly different scoring methods; *p* < 0.001) based on 95 randomly selected trees. A blossom heat map was created to demonstrate tree‐level floral differences in the orchard on 16/04/2024 (Figure 8g). Other floral traits (e.g. tree‐level Volume_Blossom_) are also provided (Tables S14 and S15).

### A pilot study for expanding the pipeline with deep learning to detect apples

To further demonstrate the usefulness of the OrchardQuant‐3D pipeline, as well as the unique value of fusing LiDAR‐ and drone‐collected point clouds for 3D orchard mapping, we conducted a pilot study to carry out large‐scale apple detection using the pipeline. As the accuracy of colour‐based thresholding could be affected by nature illuminance and leaf/branch occlusions during fruit development, we therefore explored the integration of DL models into the pipeline for 3D fruit detection.

**Figure 9:**
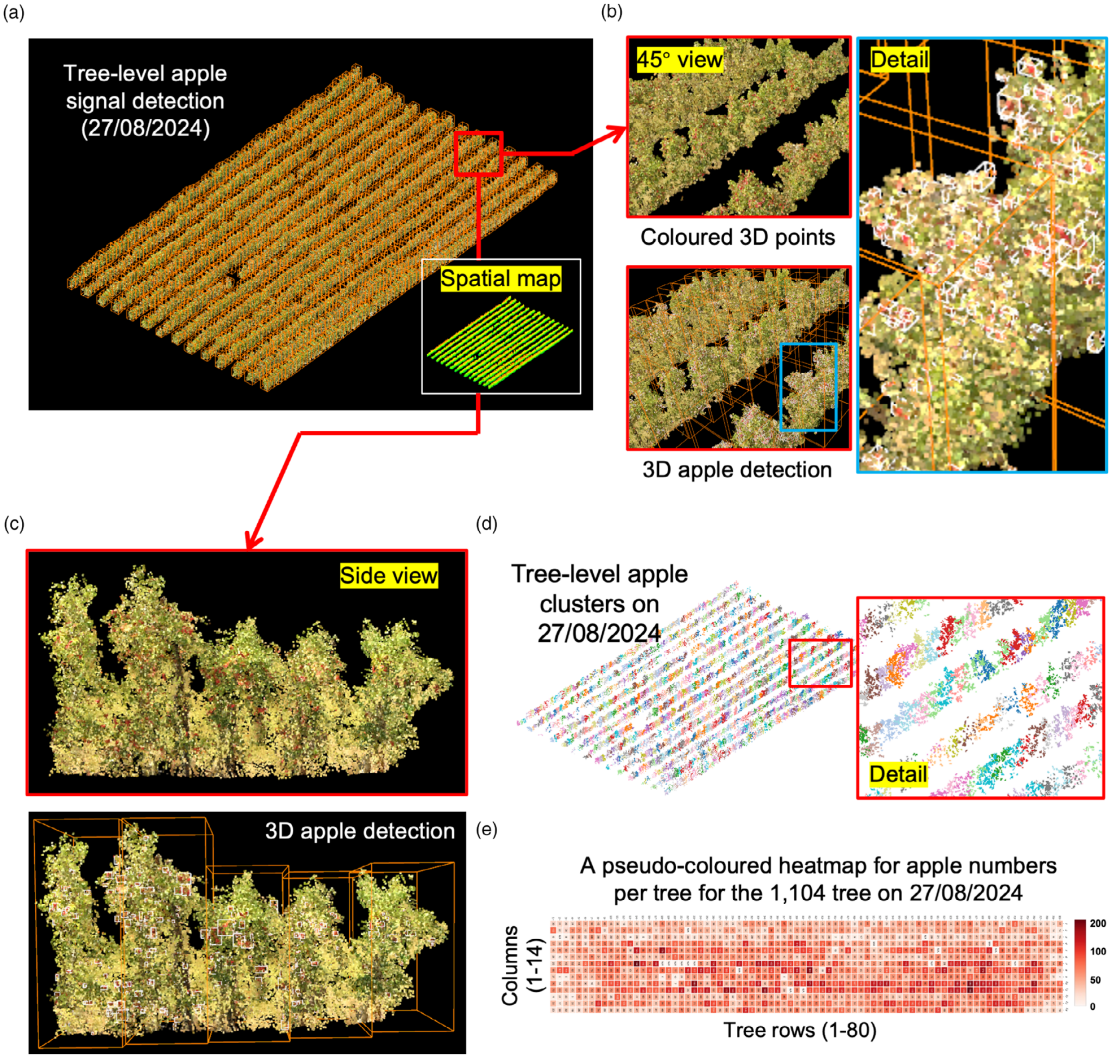
The extension of the OrchardQuant‐3D pipeline to detect fruits in the orchard using a standard deep learning model. (a) 3D coloured point clouds of 1104 apple trees collected by drones together with a pseudo‐coloured spatial map on 27/08/2024. (b, c) tree‐level apple objects segmented, followed by 3D bounding boxes applied to identified apple objects, with both 45‐degree and side views. (d) Tree‐level apple objects used to compute key fruit traits such as tree‐level apple numbers and volumes. (e) A pseudo‐coloured heat map produced to show tree‐level apple numbers of the orchard.

Based on drone‐collected 3D points acquired on 27/08/2024 (Figure 9a), we first randomly selected 341 trees in the orchard, with around 70% of the trees (n = 238) for training and 30% for testing (n = 103). Then, based on 3D points' CIELAB (i.e. LAB) values (Tkalcic and Tasic, 2003), we divided the 3D points into two categories (i.e. apples and others) using CloudCompare (Girardeau‐montaut, 2019), with misclassified and fragmented apple clusters removed. Next, a standard Stratified Transformer model (Lai *et al*., 2022) was trained using the annotated 3D points, followed by the application of the trained DL model to segment apple‐related 3D points in the orchard together with multi‐scale feature fusion and hierarchical attention mechanisms. The detection of apple‐like objects (enclosed by white‐coloured 3D bounding boxes; Figure 9b,c; Tables S16 and S17) was then assessed by evaluation metrics such as mean Intersection over Union (mIoU = 0.885) and multiply‐Accumulate Operations (mAcc = 0.925), whose results indicated highly accurate 3D apple points detection. Finally, we integrated the trained DL model into the pipeline, so that we could separate detected 3D apple points according to individual trees (Figure 9d). This led to the production of an apple number heat map showing tree‐level fruit production differences of the 1104 trees (Figure 9e). Furthermore, correlation analyses were performed between tree‐level apple cluster numbers and volumes (27/08/2024) against tree‐level blossom cluster numbers and volumes (24/04/2024) using *Pearson*'s correlation coefficient (*r*), resulting in correlated tree‐level apple and blossom numbers (*r* = 0.606) and volumes (*r* = 0.677), even though data collections were 4 months apart (Figure S12.1, Note S12). The above study suggests that the OrchardQuant‐3D pipeline can be easily extended to other key growth stages such as fruit development with the addition of new modules and functions powered by traditional techniques and DL models.

## Discussion

The advance of large‐scale and high‐throughput phenotyping technologies presents clear applications to speed up orchard fruit breeding and precision tree management. Selecting desired canopy‐level characteristics (e.g. crown volume and branch number) can have direct impacts on light interception and air circulation, helping breeders and growers develop varieties for various tree vigour, disease resistance levels and adaptabilities to diverse environments (Liu *et al*., 2021). Similarly, consistent and optimal blossom production together with flowering timing and density can improve the transition between flowers to fruit sets, which have major benefits for managing fruit production (Kumara *et al*., 2019). Hence, how to optimise canopy‐level and key floral traits will help us improve planting density, tree pruning and flowering thinning to optimise fruit production and quality.

This study presents a reliable and scalable solution for the above missions, which combined drone‐ and LiDAR‐collected datasets to perform multi‐source and scalable 3D mapping to study different types of pear and apple trees in two distinct orchards. By fusing multi‐source datasets, we were able to position trees with mm‐level accuracy and perform automated tree‐level analysis of key canopy and floral traits, which led to the quantification of varietal differences of pear cultivars in China and large‐scale floral and fruit analysis of over a thousand apple trees in the United Kingdom. This advanced our ability to accomplish comprehensive 3D phenotyping of tree‐level analysis for different types of orchards and fruit trees and thus empower researchers, breeders and growers to select desired canopy structure, blossom and fruit characteristics for orchard breeding and precise tree management, which, to the best of our knowledge, has not yet been introduced to this research domain.

### Multi‐source data fusion for 3D trait analysis

Multi‐source data fusion (e.g. RGB, multi‐spectral imaging, 3D point clouds, near‐infrared and Raman spectroscopy) offers a multifaceted approach that can assist us to assess plant performance from various perspectives, leveraging the strengths of different data types to provide a more comprehensive and in‐depth understanding of target traits of interest for breeders, growers and plant researchers (Comba *et al*., 2019; Xu *et al*., 2023). In this study, we registered drone‐derived colour point clouds with LiDAR‐collected high‐quality 3D points, so that spatial and colour information can be combined to facilitate the analysis of canopy and floral traits. This data fusion approach helped us to: (1) use accurate GNSS signals collected by LiDAR to update geo‐information of drone‐derived point clouds, leading to mm‐level tree positioning after segmenting trees in different types of orchards; (2) assemble multi‐source 3D points to build more precise 3D tree models to mitigate obstructed views from the air (drones) and ground (LiDAR); (3) assign precise drone‐collected colour information to LiDAR 3D points, enabling the addition of spectral signals to spatial signals with reduced errors and increased confidence in the analysis. Moreover, multi‐source data fusion assisted us to combine tree‐level phenotypic features at different growth stages. For example, tree‐level morphological features at the bud burst stage were integrated into the analysis of floral and fruit characteristics (e.g. blossom and apple clusters) at flowering and fruiting stages, which offered unprecedented insights into the growth dynamics of target yield and quality traits that were impossible to measure previously.

### Scalable tree‐level phenotypic analysis

We developed various new methods to perform tree‐level trait analysis in fruit trees, ranging from the establishment of hyperplanes with a gridding system to align and position tree trunks, to the application of complex 3D graph theory and feature engineering to quantify branches and branch‐level blossom phenotypic features. The tree positioning method helped us enhance the accuracy of 3D tree mapping, achieving mm‐level resolution when locating trees in the orchard. This can further enable accurate spatial analysis of tree density, tree‐level growth, blossom and yield distribution, and potentially disease spread. In particular, many toolkits are now equipped with different GPS systems, making it problematic to understand location‐specific factors across an orchard. Hence, our approach demonstrates that it is feasible to integrate signals from multiple sources, with different spatial resolutions, through a gridding system, which can then be used to consistently examine phenotypic variations collected by different devices. Moreover, when the tree positions are precisely recorded, it is easier to repeatedly measure tree‐level developmental patterns and their interactions with the environment (e.g. soil properties and micro‐climate conditions) over time, which is essential to ecological studies and predictive modelling to understand genetics (G), environment (E) and agronomic management (M) and their interactions (G × E × M) for multi‐seasonal fruit orchard improvement and associated fruit productivity.

The 3D graph theory based on the 3D skeletonization method took into consideration supporting structures and skeleton gaps caused by leaf and branch occlusions. On the one hand, it helped us derive a comprehensive 3D representation of tree morphological features; on the other hand, it enabled us to computationally study traits at both tree and branch levels, which can empower researchers to measure tree growth and development together with structural adaptations in greater detail. In particular, a precisely positioned 3D tree model can be utilised to assess growth dynamics (e.g. rates of change) of canopy volume, leaf area index (LAI) and branch angles across different growth stages and seasons, enabling an in‐depth understanding of tree growth and structural development on a scale that is previously impossible. These novel advances will be important for supporting and improving fruit tree breeding to address the present and future challenges in the field (Chakraborty *et al*., 2019), including the exploration of climate‐resilient traits and the implementation of precise orchard management strategies. Hence, we trust that the OrchardQuant‐3D method will contribute to the development of a more productive and sustainable orchard system.

### Key floral traits and orchard‐level trait analysis

The detailed analysis of key floral traits together with fruit estimation can form the foundation for researchers and growers to evaluate whether blossoms are pollinated effectively and the transition rate from blossoms into mature fruits, which is a key orchard management task in order to maximise yield production (Solomakhin and Blanke, 2010). This study presented a highly accurate blossom detection method together with geo‐positioning blossom clusters and fruit detection, which can guide us to implement precise thinning applications for blossom or fruitlet reduction according to blossom loads and canopy structures of individual trees. For example, using the blossom detection and localisation method, the dose of chemical thinning agents can be calculated and tailored to individual trees across orchards according to the blossom load and canopy structures. This can be further combined with precise variable rate spray technology, chemical thinning agents (or other sprayable products such as pesticides) based on tree‐level requirements, helping optimise fruit yield and quality for different types of orchards and thus indicate the real‐world impacts of the OrchardQuant‐3D pipeline.

It is important to point out that orchards are not homogenous. There are substantial variations in canopy and floral parameters between individual trees and sections within an orchard due to soil properties (Perry *et al*., 2010) and varied topographic patterns (Aggelopoulou *et al*., 2010). Traditionally, the orchard‐level variations were difficult to quantify due to the lack of suitable phenotyping toolkits, requiring laborious manual assessment, followed by relatively subjective pruning and thinning decisions for orchard management (Giles *et al*., 1987). Hence, the orchard‐level 3D mapping capability presented in this study is highly valuable, creating high‐resolution fruit tree treatments for: (1) the identification of trees or zones overloaded with blossoms that could lead to small or low‐quality fruitlets, facilitating zone‐based blossom thinning; (2) enabling precision agronomic management (e.g. spray systems) with variable rates to improve application efficiency (e.g. chemical or mechanical thinning), which will lead to reduced costs and increased yields. Furthermore, from a genetic breeding perspective, the lack of orchard‐level methods to study key canopy, floral and fruit traits and their changes during GxExM has limited breeders' ability to effectively select preferred genotypes (Allard *et al*., 2016), reducing the pace of identifying genetic factors (e.g. genetic loci) for marker‐assisted selection (MAS) for orchard improvement (Ru *et al*., 2015). Hence, the OrchardQuant‐3D method is also likely to help breeders develop precise tree planting and variety planning for tree breeding programmes.

### Limitations and future work

It is important to note the limits of this study and suggest future developments. Based on the applications of the OrchardQuant‐3D method in both apple and pear orchard studies, we concluded the following matters that are worth considering: (1) the integration of GNSS information can accurately locate individual trees in the orchards, which suggests the importance of utilising an RTK device or drones equipped with RTK technology if mm‐level tree positioning is required; (2) although we demonstrated that the OrchardQuant‐3D pipeline was able to study fruit development using DL‐powered modules and functions, we did not conduct an in‐depth study after the flowering stage (i.e. the transition from blossoms to mature fruits) and relevant tree‐level yield studies because the focus of our study was on canopy (e.g. tree pruning) and floral traits (e.g. flower thinning); (3) we believe the DL‐powered approach combining colour with spatially oriented features can be easily continuously improved in future developments, linking different key growth stages during orchard growth and development for more accurate predictions of marketable yield; still, further testing is required to assess how the system responds to edge cases (e.g. very sparse or overlapping tree structures); (4) the branch analysis algorithm developed in this study has not been systematically assessed by pear and apple trees with significantly different shapes and structures, indicating the generalisation and robustness of our work can be improved and optimised in future studies; (5) it is also important to incorporate key environmental factors (soil conditions, solar radiation, rainfall and pollination) into the assessment of orchard performance to promote precision orchard management, delivering new insights into drivers of phenotypic variability and 3D orchard mapping to monitor fruit yield formation; and (6) the data‐fusion solution helped us minimise branch and leaf occlusion for the production of high‐quality 3D tree skeletons, but the approach could be further improved using DL‐powered point cloud completion methods (Chen *et al*., 2023) when detecting fruitlets and fruits (extremely valuable metrics for growers and fruit marketing groups) prior to harvest as fruits are commonly occluded by leaves, branches and neighbouring fruits.

Besides the above areas for improvement, the OrchardQuant‐3D method could be improved by the addition of multi‐ and hyper‐spectral signals. Through data‐fusion algorithms developed in the study, multi‐source signals will be able to provide an extra layer of information to assist precise irrigation and fertilisation plans to maintain healthy tree canopies, manage blossom density through thinning to balance yield and fruit quality, and empower pest and disease controls by highlighting problematic varieties and underperforming areas with spectral signatures (Talebpour *et al*., 2019). Furthermore, the potential of combining data sources (RGB, multi‐ or hyper‐spectral, 3D) from different time points in the orchard can be enormous. For example, fruitlets on the trees can be occluded by many leaves; however, the blossom detection is easier than fruitlets due to less leaf occlusion. Hence, by combining tree morphology before and after flowering with blossom density and fruitlets, it is possible to mitigate obstructed views of fruitlets with previous knowledge of blossoms. Moreover, such a combined approach is likely to facilitate us in monitoring temporal changes of key yield‐related factors, from blossoms to fruitlets and fruits, making yield prediction more accurate for fruit production.

## Conclusion

This study presents the OrchardQuant‐3D method, an automated and scalable analysis pipeline that incorporates data‐fusion, GNSS‐RTK tree positioning, complex 3D graph theory and various feature engineering algorithms to combine drone‐derived and LiDAR‐collected 3D points for performing tree‐ and orchard‐level phenotypic analysis of canopy, floral and fruit traits for orchard fruits such as pear and apple. We demonstrated that the use of spatial and colour information collected by drones and LiDAR devices could enable automated trait analysis with a mm‐level accuracy, which facilitated the quantification of varietal differences of different pear cultivars and large‐scale apple orchard surveillance for blossom and fruit assessment. To the best of our knowledge, such a comprehensive analytic methodology has not yet been introduced to 3D orchard phenotyping. Hence, we trust that our work demonstrates a step change in our ability to conduct multi‐source and scalable 3D orchard phenotyping, which will greatly advance and benefit areas such as orchard fruit breeding, digital agronomy and precise crop management to optimise orchard productivity and relevant orchard research under complex field conditions.

## Funding

The pear orchard work in Nanjing was supported by the Jiangsu Agricultural Science and Technology Innovation Fund (CX(22)2025 to NAU). The apple orchard work at East Malling together with Robert Jackson, Charles Whitfield and Ji Zhou was both supported by Innovate UK's precision orchard management and environment (POME) grant (IUK 10072930 to NIAB). Ji Zhou, Robert Jackson and Greg Deakin were also partially supported by One CGIAR's SeedEqual Initiative (5507‐CGIA‐07 to Ji Zhou), as well as the United Kingdom Research and Innovation's (UKRI) Biotechnology and Biological Sciences Research Council (BBSRC) AI in Plant Research Grant (BB/Y513969/1 to Ji Zhou). The machine learning modelling was partially conducted on the CropDiversity infrastructure, supported by the BBSRC's ALERT Grant (BB/X019683/1 to JHI). The bilateral research activities were partially supported by the BBSRC's International Partnership Grant (BB/Y514081/1 to NIAB).

## Conflict of interest

The authors declare no competing financial interests.

## Author contributions

Ji Zhou, Yunpeng Xia, Hanghang Li and Charles Whitfield wrote the manuscript with inputs from all the authors; Fanhang Zhang, Gang Sun, Kaijie Qi, Robert Jackson and Felipe Pinheiro conducted field experiments, drone and LiDAR phenotyping, and ground truthing in China and the United Kingdom under Shaoling Zhang, Charles Whitfield, Shutian Tao and Ji Zhou's supervision; Yunpeng Xia, Hanghang Li, Gang Sun, Xiaoman Liu, Yue Mu and Ji Zhou developed algorithms and modules in the OrchardQuant‐3D method; Yunpeng Xia, Hanghang Li, Fanhang Zhang, Gang Sun and Greg Deakin performed statistical analysis and result interpretation under Ji Zhou's supervision. All authors read and approved the manuscript. Yunpeng Xia, Hanghang Li and Fanhang Zhang contributed equally to this work.

## Supporting information


**Note S1** The drone flight parameters applied in China and the UK orchards.
**Note S2** LiDAR software suite used in data pre‐processing.
**Note S3** The identification of missing trees or dead trees in the orchard.
**Note S4** The computation of geo‐coordinates of the 70 pear trees in the orchard.
**Note S5** Data fusion results using 3D point clouds collected by drone and LiDAR.
**Note S6** Removing tree‐level supporting structures at the tree level.
**Note S7** The quantification of tree branches.
**Note S8** Adaptive parameterisation and hard‐coded values.
**Note S9** The graphical user interface of OrchardQuant‐3D.
**Note S10** Impacts of support structures when measuring tree height and crown volume.
**Note S11** Comparisons of different methods for quantifying tree branches.
**Note S12** Statistical analysis of tree‐level canopy, floral, and fruit traits.
**Table S1** Crown volume (m^3^) of 70 pear trees in the orchard in 2023.
**Table S2** Surface area (m^2^) of 70 pear trees in the orchard in 2023.
**Table S3** Canopy diameter (cm) of 70 pear trees in the orchard in 2023.
**Table S4** Canopy projected area (m^2^) of 70 pear trees in the orchard in 2023.
**Table S5** The number of flower clusters of 70 pear trees in the orchard.
**Table S6** The volume of flower clusters of 70 pear trees in the orchard (m^3^).
**Table S7** Surface area of flower clusters of 70 pear trees in the orchard (m^2^).
**Table S8** Projection area of flower clusters of 70 pear trees in the orchard (m^2^).
**Table S9** Branch‐level blossom cluster analysis with both geo‐positions and trait analysis.
**Table S10** Tree‐level canopy volume (m^3^) measured in the apple orchard on 16 April 2024.
**Table S11** Tree‐level canopy volume (m^3^) measured in the apple orchard on 24 April 2024.
**Table S12** Tree‐level blossom cluster number measured in the apple orchard on 16 April 2024.
**Table S13** Tree‐level blossom cluster number measured in the apple orchard on 24 April 2024.
**Table S14** Tree‐level blossom cluster volume (m^3^) measured in the apple orchard on 16 April 2024.
**Table S15** Tree‐level blossom cluster volume (m^3^) measured in the apple orchard on 24 April 2024.
**Table S16** Tree‐level apple number in the orchard on 27 August 2024.
**Table S17** Tree‐level apple volume (m^3^) in the orchard on 27 August 2024.

## Data Availability

The data that support the findings of this study are openly available in OrchardQuant‐3D at https://github.com/The‐Zhou‐Lab/OrchardQuant‐3D/releases.
