# Supplementary material for: OrchardQuant‐3D: combining drone and LiDAR to perform scalable 3D phenotyping for characterising key canopy and floral traits in fruit orchards
Source: Plant Biotechnol J. 2025 Jul 23;23(11):4910–29. doi: 10.1111/pbi.70229 (PMC12576445; doi:10.1111/pbi.70229)
Supplement: Supplementary file 1 — Note S1 The drone flight parameters applied in China and the UK orchards. Note S2 LiDAR software suite used in data pre‐processing. Note S3 The identification of missing trees or dead trees in the orchard. Note S4 The computation of geo‐coordinates of the 70 pear trees in the orchard. Note S5 Data fusion results using 3D point clouds collected by drone and LiDAR. Note S6 Removing tree‐level supporting structures at the tree level. Note S7 The quantification of tree branches. Note S8 Adaptive parameterisation and hard‐coded values. Note S9 The graphical user interface of OrchardQuant‐3D. Note S10 Impacts of support structures when measuring tree height and crown volume. Note S11 Comparisons of different methods for quantifying tree branches. Note S12 Statistical analysis of tree‐level canopy, floral, and fruit traits. Table S1 Crown volume (m3) of 70 pear trees in the orchard in 2023. Table S2 Surface area (m2) of 70 pear trees in the orchard in 2023. Table S3 Canopy diameter (cm) of 70 pear trees in the orchard in 2023. Table S4 Canopy projected area (m2) of 70 pear trees in the orchard in 2023. Table S5 The number of flower clusters of 70 pear trees in the orchard. Table S6 The volume of flower clusters of 70 pear trees in the orchard (m3). Table S7 Surface area of flower clusters of 70 pear trees in the orchard (m2). Table S8 Projection area of flower clusters of 70 pear trees in the orchard (m2). Table S9 Branch‐level blossom cluster analysis with both geo‐positions and trait analysis. Table S10 Tree‐level canopy volume (m3) measured in the apple orchard on 16 April 2024. Table S11 Tree‐level canopy volume (m3) measured in the apple orchard on 24 April 2024. Table S12 Tree‐level blossom cluster number measured in the apple orchard on 16 April 2024. Table S13 Tree‐level blossom cluster number measured in the apple orchard on 24 April 2024. Table S14 Tree‐level blossom cluster volume (m3) measured in the apple orchard on 16 April 2024. Table S15 Tree‐level blossom [file PBI-23-4910-s001.docx]

# Supporting Information

*Notes, Figures, Tables and References*

**OrchardQuant-3D: combining drone and LiDAR to perform scalable 3D phenotyping for characterising key canopy and floral traits in fruit orchards**

## Authors:

Yunpeng Xia^1,+^, Hanghang Li^1,+^, Fanhang Zhang^2,+^, Gang Sun^1^, Kaijie Qi^2^, Robert Jackson^3^, Felipe Pinheiro^3^, Xiaoman Liu^4^, Yue Mu^1^, Shaoling Zhang^2^, Greg Deakin^3^, Charles Whitfield^3,*^, Shutian Tao^2,*^, Ji Zhou^1,4*^

+ Contributed equally; * Corresponding authors

## Affiliations:

^1^College of Engineering, Academy for Advanced Interdisciplinary Studies, Plant Phenomics Research Centre, Nanjing Agricultural University, Nanjing 210095, China

^2^College of Horticulture, State Key Laboratory of Crop Genetics and Germplasm Enhancement, Nanjing Agricultural University, Nanjing 210095, China

^3^Data Sciences, East Malling Research (EMR), National Institute of Agricultural Botany (NIAB), Crop Science Centre (CSC), Cambridge CB3 0LE, United Kingdom

^4^College of Sciences, Nanjing Agricultural University, Nanjing 210095, China

## Corresponding authors:

Charles.Whitfield@niab.com; TaoST@njau.edu.cn; Ji.Zhou@NJAU.edu.cn or Ji.Zhou@NIAB.com

## Supplementary Notes:

### Note S1 – The drone flight parameters applied in China and the UK orchards.

In this study, we employed the drone flight methodology adopted from previous research (Sun *et al.*, 2022) and utilised DJI GS PRO software (DJI, Shenzhen, China) to plan orthogonal flight paths for data collection in pear and apple orchards. After defining the orchard area in the DJI GS PRO software, we extended the boundaries by 2-3 meters to ensure the coverage of aerial phenotyping. The camera orientation was aligned with the flight path, and the drone was set to waypoint hovering mode for photography, maintaining a flight altitude of 8 m (12 m for apple orchard), a forward overlap ratio of 80%, a side overlap ratio of 75%, and a gimbal angle of 45°. After executing the first flight mission, the flight path angle was rotated by 90° for the second flight to complete data acquisition missions.

### Note S2 – LiDAR software suite used in data pre-processing.

We used the software bounded with the Robin system to generate raw point clouds of the orchards, following the same processing steps published previously (Zhu *et al.*, 2021). The steps include: (1) the raw data imported into the folder created by the MMPprocess software; (2) the AEROoffice software used to generate the acquisition path and optimise the GNSS data quality through grafNav; (3) AEROoffice used to process signal breakpoints and stationary data; (4) the MMPprocess software used to generate raw point clouds and associate LiDAR-recorded global navigation satellite system (GNSS) signals with the point clouds. Point clouds were further processed using the TerraSolid software (Korzeniowska and Łącka, 2011), which removed noise points, classified and calibrated point clouds, and finally saved the processed point clouds in a LAS file.

### Note S3 – The identification of missing trees or dead trees in the orchard.

During the data acquisition, drone and LiDAR could encounter missing pear and apple trees in the orchard. Hence, we developed a method to address these missing trees points from canopy height model (CHM; **Fig S3.1**). By applying the gridding method, we obtained the tree centroid points within every grid (**Fig S3.2**; left). Then, we performed a perspective transformation to obtain the rotation matrix of point clouds collected by LiDAR and drones. If point clouds were not found or shorter than 1.2 m high (i.e. a dead tree) in a given grid (**Fig S3.2**; right), the grid would be treated as a missing tree (coloured red) in the orchard.


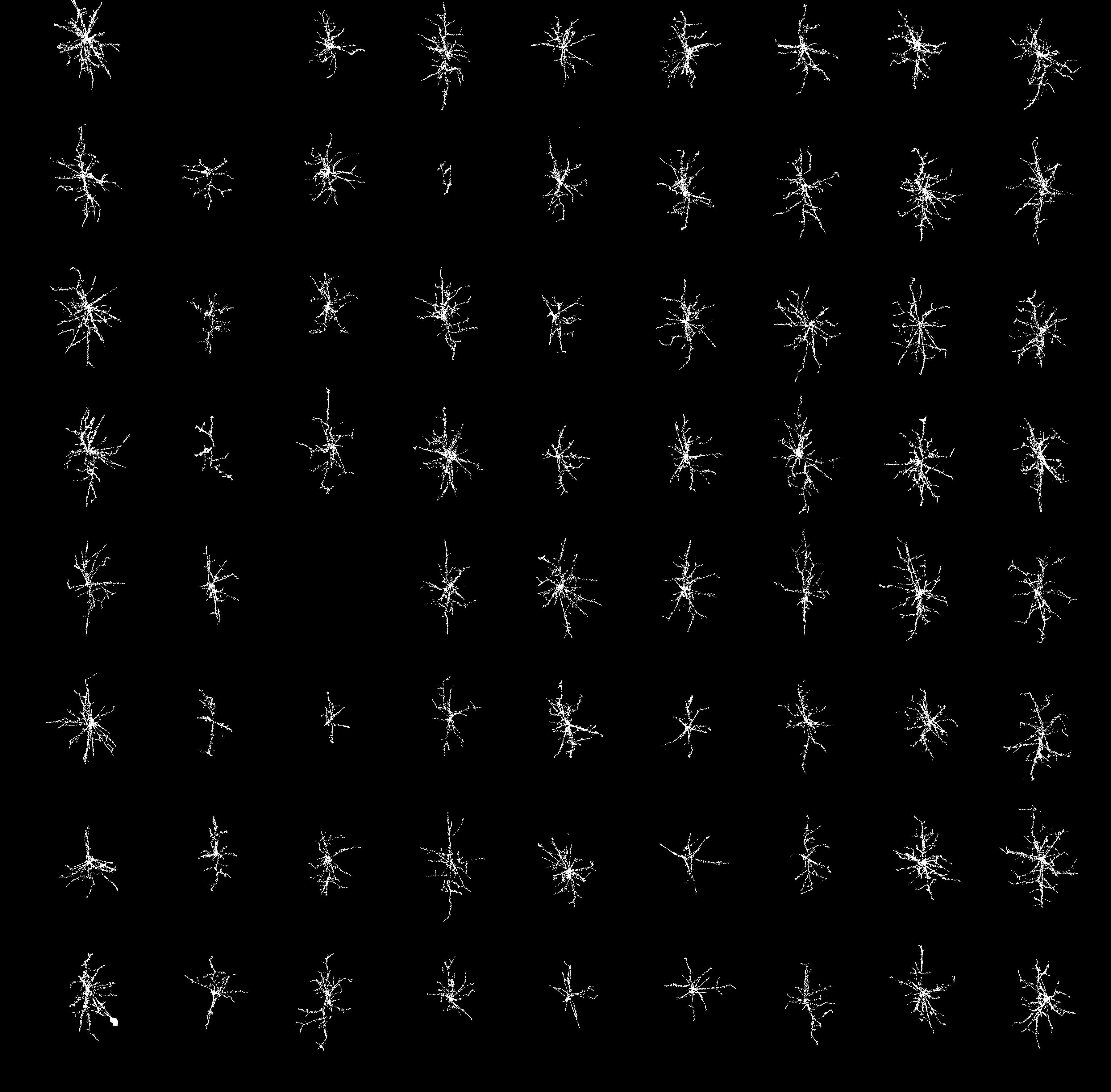


**Gaps caused by dead or undeveloped trees**

**Figure S3.1** Canopy height model showing missing tree data collected by LiDAR

**Figure S3.2** Missing trees or gaps caused by dead trees

### Note S4 – The computation of geo-coordinates of the 70 pear trees in the orchard.

Tree trunks were aligned and placed at the centre of grids in a unified gridding system. Then, x and y coordinates (float values) of the centroids of 70 pear trees in the gridding system was assigned based on the GNSS signals obtained by a backpack LiDAR, with a geo-coordinate system of the Geocentric Datum of Australia 1994 (GDA94). The table below lists the geo-coordinates of the pear trees in the orchard, positioning trees with mm-level accuracy.

| **Col**  **Row** | **1** | **2** | **3** | **4** | **5** | **6** | **7** | **8** | **9** |
| --- | --- | --- | --- | --- | --- | --- | --- | --- | --- |
| **1** | x=**-**2185249.701  y=13857619.429 | x=-2185246.489  y=13857616.746 | x=-2185243.913  y=13857614.433 | x=-2185240.828  y=13857611.831 | x=-2185237.984  y=13857609.367 | x=-2185234.855  y=13857606.825 | x=-2185232.305  y=13857604.246 | x=-2185229.301 y=13857601.870 | x=-2185226.431  y=13857599.216 |
| **2** | x=-2185246.891  y=13857622.577 | x=-2185243.703  y=13857620.069 | x=-2185240.994  y=13857617.620 | x=-2185238.163  y=13857615.027 | x=-2185235.385  y=13857612.399 | x=-2185232.093  y=13857610.056 | x=-2185229.269  y=13857607.624 | x=-2185226.453  y=13857605.164 | x=-2185223.544  y=13857602.579 |
| **3** | x=-2185244.006  y=13857625.952 | x=-2185240.889  y=13857623.413 | x=-2185238.102  y=13857620.951 | x=-2185235.071  y=13857618.548 | x=-2185232.340  y=13857615.909 | x=-2185229.408  y=13857613.257 | x=-2185226.386  y=13857610.903 | x=-2185223.513  y=13857608.311 | x=-2185220.710  y=13857605.736 |
| **4** | x=-2185241.066  y=13857629.280 | x=-2185238.013  y=13857626.695 | N/A | x=-2185232.380  y=13857621.677 | x=-2185229.528  y=13857619.320 | x=-2185226.372  y=13857616.716 | x=-2185223.584  y=13857614.133 | x=-2185220.804  y=13857611.594 | x=-2185217.694  y=13857609.206 |
| **5** | x=-2185238.267  y=13857632.537 | x=-2185235.253  y=13857629.955 | x=-2185232.346  y=13857627.554 | x=-2185229.577  y=13857624.901 | x=-2185226.777  y=13857622.462 | x=-2185223.699  y=13857619.870 | x=-2185220.829  y=13857617.417 | x=-2185217.975  y=13857614.827 | x=-2185214.963  y=13857612.414 |
| **6** | x=-2185235.397  y=13857635.943 | x=-2185232.317  y=13857633.275 | x=-2185229.399  y=13857630.883 | x=-2185226.731  y=13857628.224 | x=-2185223.868  y=13857625.940 | x=-2185220.802  y=13857623.151 | x=-2185217.895  y=13857620.550 | x=-2185215.055  y=13857618.159 | x=-2185212.123  y=13857615.603 |
| **7** | x=-2185232.597  y=13857639.208 | x=-2185229.400  y=13857636.657 | x=-2185226.535  y=13857634.167 | x=-2185223.863  y=13857631.494 | x=-2185221.002  y=13857629.145 | x=-2185218.016  y=13857626.355 | x=-2185215.046  y=13857624.000 | x=-2185212.188  y=13857621.378 | x=-2185209.041  y=13857619.047 |
| **8** | x=-2185229.730  y=13857642.552 | N/A | x=-2185223.693  y=13857637.400 | x=-2185221.060  y=13857634.759 | x=-2185218.053  y=13857632.367 | x=-2185215.062  y=13857629.791 | x=-2185212.082  y=13857627.353 | x=-2185209.235  y=13857624.799 | x=-2185206.346  y=13857622.111 |

### Note S5 – Data fusion results using 3D point clouds collected by drone and LiDAR.

We used registration algorithms to merge 3D point clouds collected by the two sources. Tree 8 (R1, C8) and Tree 62 (R7, C9), with different tree structures, were used to visualise the data fusion results, for both 3D tree skeletons and point clouds (**Fig. S5**). Through data fusion, we have calibrated and eliminated spatial differences caused by different geographic coordinate systems used by the drone and LiDAR. Also, due to data fusion, we could also reliably combine point clouds collected by different collection approaches and at different growth stages.


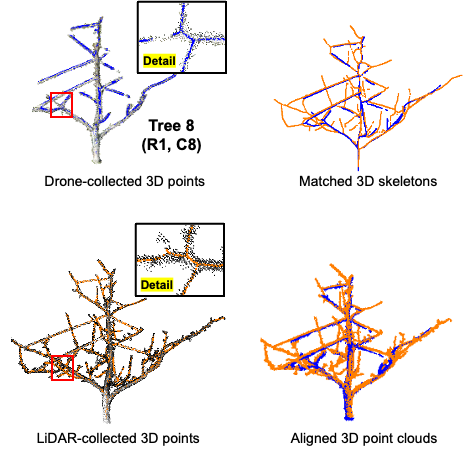

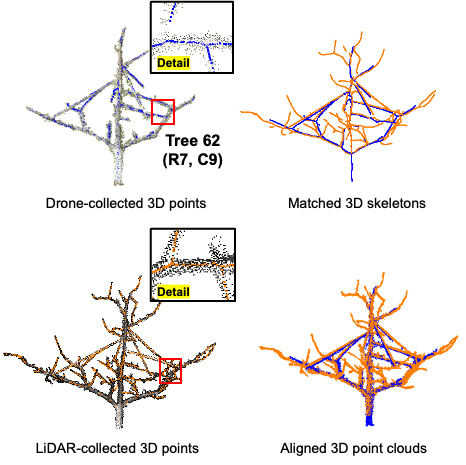


**Figure S5** Data fusion of different types of pear trees

### Note S6 – Removing tree-level supporting structures at the tree level.

*1. Remove supporting structures*

In the orchard, support structures were installed using the same standardised fixed method. The graph-based algorithm can largely identify and remove most of the supporting structures. Only some supporting structures required LiDAR intensity-based method to improve the skeleton pruning. To demonstrate the results of the two pruning methods, we selected three trees from the orchard, Tree22 (R3, C4), Tree38 (R5, C3), and Tree48 (R6, C4), whose tree morphological features were very different (**Fig. S6.1-3**; upper left and right). After applying, both graphical and intensity-based methods’ processing results were concluded below, showing the processed 3D tree skeletons, with small supporting structures in green-coloured rectangles required LiDAR intensity-based method to remove as the graph-based method could not eliminate some horizonal structures (**Fig. S6.1-3**; lower left). Results from the LiDAR intensity approach are also given (**Figure S6.1-3**; lower right).

**
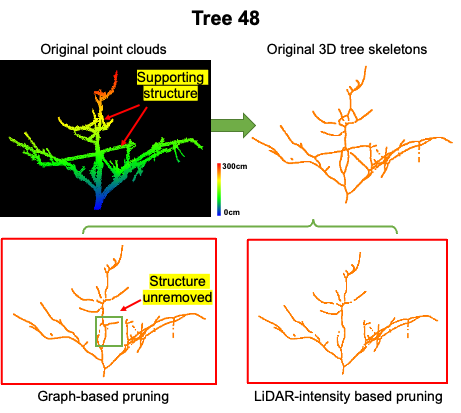
**

**Fig S6.1** Remove supporting structures for Tree 22 using both graph-based and LiDAR intensity-based methods

**
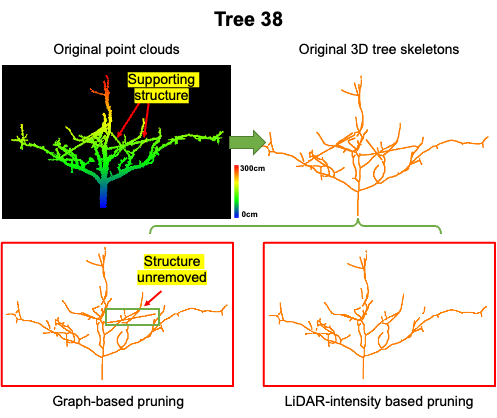
**

**Fig S6.2** Remove supporting structures for Tree 38 using both graph-based and LiDAR intensity-based methods


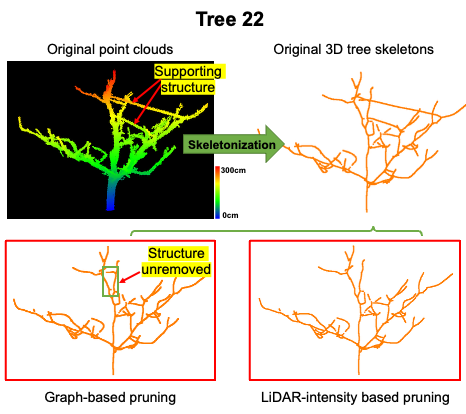


**Fig S6.3** Remove supporting structures for Tree 48 using both graph-based and LiDAR intensity-based methods

*2. Calculate normalised LiDAR intensity values*

We adopted a spatial proximity approach (Jones *et al.*, 2006) to assign normalised LiDAR intensity values to 3D skeleton points. First, we aligned 3D point clouds before and after the tree-level skeletonization. Then, each skeleton point was treated as a query centre, so that a radius-based nearest neighbor (R-NN) method (Karger and Ruhl, 2002) could be applied to identify sets of 3D points within a certain radius (i.e. the radius of the tree trunk) in the aligned point clouds. Finally, we calculated the average intensity value of all the 3D points within the scanning radius as the LiDAR intensity value for the corresponding skeleton point.

If only the surface of the point clouds possesses intensity values, we followed the following methods to computer and assign intensity values: (1) the nearest surface point search (i.e., for each skeleton point, searching for the nearest surface point) to assign intensity values; (2) a scanning radius for each skeleton point to collect all surface points within that radius, based on which the average or distance-weighted average was computed as the intensity value; (3) the division of 3D point clouds into uniform voxel grids, so that the average intensity of surface points within each voxel was calculated and assigned to skeleton points associated with the corresponding voxels.

*3. Prevent removed supporting structure points from being reintroduced when filling gaps*

After completing the removal of support structures, we conduct a spatial position comparative analysis (Novotni and Klein, 2001) between the removed supporting-structure 3D points and the newly introduced 3D points through the voxelization. By setting strict spatial distance constraints and neighbourhood criteria using the scanning range (i.e. 50% of the radius of the tree trunk in the associated tree), we effectively prevented 3D points from support structures to be reintroduced when filling gaps in the final tree skeletons using both down-sampled voxels and line interpolation (**Fig. 4e** in the main text).

### Note S7 – The quantification of tree branches.

We use a combined method to quantify the number of tree branches, including the complex graph theory and the Euclidean distance clustering. The algorithmic steps include: (1) the removal of the trunk points from the finalised 3D tree skeletons after **Note S6**; (2) using the distance set for the linear interpolation as the input parameter, we performed the Euclidean distance clustering segmentation on the remaining 3D points to separate the potential tree branches; (3) subsequently, for every separated branch, we find the outermost edge points and nearest neighbour points with branches removed; (4) finally, the Dijkstra's algorithm (Shekhar and Xiong, 2008) was applied to locate the longest path between outermost points and nearest neighbour points, which was treated as a tree branch. After identifying a branch, we repeated the above steps until all the branches of a given tree were recognised, iteratively.

### Note S8 – Adaptive parameterisation and hard-coded values.

When quantifying tree branches and removing supporting structures, we optimised the point cloud differentiation thresholding (i.e. between tree branches and support structures) through a grid-based search, the range was set as [1.0, 2.0] with a step size of 0.1. We found that LiDAR-based intensity value with a multiplier threshold of 1.1 yielded the most optimal F1score (>0.8) and Intersection over Union (IoU > 0.7).

When performing the blossom clustering, we set 20 3D points as our clustering threshold, which was due to the manual assessment of single blossom clusters collected from the 95 trees in the orchard. The experiential constant also helped us achieve the best balance between preserving flower clusters and filtering noise. Notably, depending on the size of the blossom clusters (i.e. resolutions of the 3D mapping), the 20-point setting might need to be altered for different 3D orchard mapping. Additionally, we optimised hard-coded parameters through adaptive parameterisation. For example, we used the radius and diameter of a given tree trunk as the searching radius for radius-based nearest neighbor algorithm for tree-level structure, canopy, and branch assessment. The average point spacing in the 3D point clouds used for linear interpolation to fix gaps in the tree skeletons was also followed the same approach, enabling us to enhance the algorithm's robustness and adaptability.

After extensive testing, the hard-coded values and experiential constant we pre-configured for key parameters are: (1) hyperplane projection ratio, which was used to select 3D point clouds from the bottom 10% of the trunk in the z-direction; (2) the number of iterations for registering drone- and LiDAR-collected point clouds was set to 50; (3) based on manually assessed tree structures, thresholding for obtaining the outermost edge points was set to 0.02 m and 0.05 m, respectively; (4) the size of voxels was set to 0.03 m, which were based on the density in the collected 3D point clouds; (5) the Euclidean distance clustering threshold was set to 0.05 m; and, (6) the grid size of flower cluster voxel was set to 0.1 m according to manual measures from 95 trees selected in the orchard.

### Note S9 – The graphical user interface of OrchardQuant-3D.

We created a graphical user interface (GUI) for non-expert users to utilise our work. The GUI for the OrchardQuant-3D pipeline was developed using the Python native GUI package, Tkinter (Shipman, 2013) and can be executed on Microsoft Windows operating system (Windows 10 and 11 tested). The executable file (Gui.ipynb) can be downloaded from our laboratory’s GitHub repository (https://github.com/The-Zhou-Lab/OrchardQuant-3D/releases).

*1. Input files*

On the GUI (**Fig. S9.1**; left), users first need to select the path to the LiDAR point cloud files. The LiDAR path includes point cloud files in LAS or LAZ format, and SHP files containing 3D or geographic coordinate information (RTK data). Then, users need to set the output file path, where multiple folders will be automatically generated to save the results. Finally, after the user has selected the paths (**Fig. S9.1** right), clicking the “Next” option to proceed.


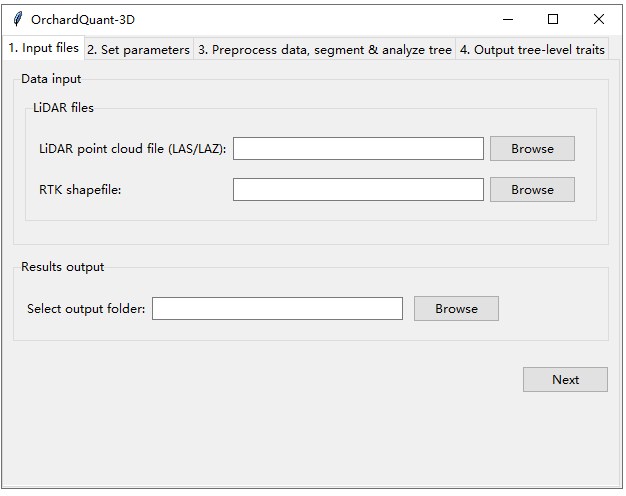

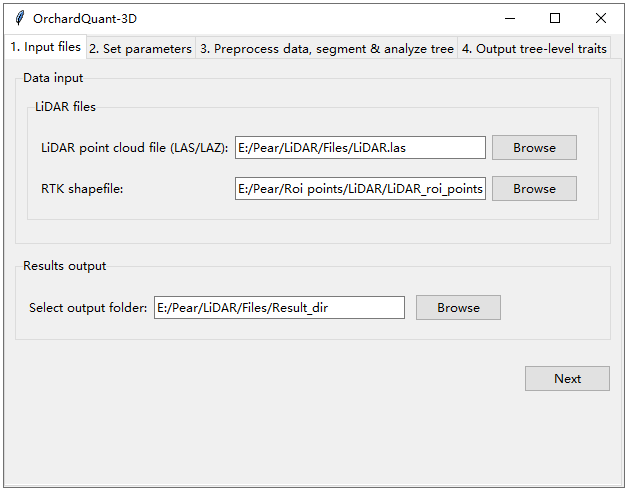


**Figure S9.1** Screenshots of OrchardQuant-3D’s input files section

*2. Set parameters*

Users can customize parameters in the interface, which are pre-filled in the GUI tailored for the resolution and density of our 3D orchard point clouds (testing file can be downloaded from our GitHub repository, see **Note S8**). Key values include parameters for the Statistical Outlier Removal (SOR) denoising algorithm, the Cloth Simulation Filter (CSF) ground point filtering algorithm (**Fig. S9.2**), as well as the spatial resolution to generate the Canopy Height Model (CHM) of the 3D orchard. Input parameters for tree skeletonization are based on the semantic Laplacian algorithm (Meyer *et al.*, 2023), which can be set in the GUI for weighted coefficients and downsampling grid size. Parameters for canopy-level traits (e.g. branches) are provided.


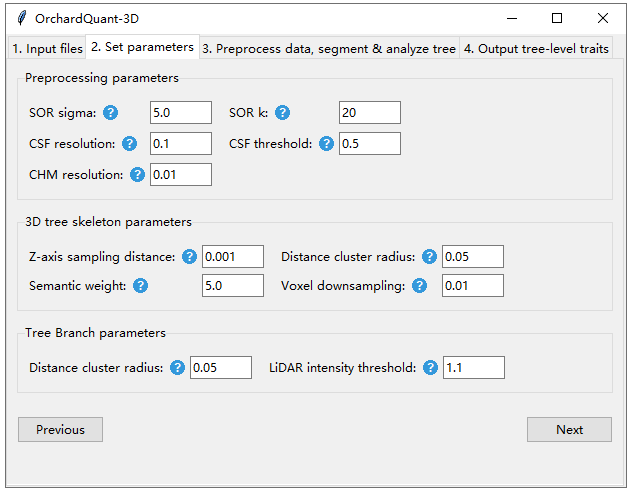


**Figure S9.2** Set input parameters for the OrchardQuant-3D GUI

Users can click the blue information icon buttons in the GUI to get detailed explanations of corresponding input parameters, making it easier to understand why and how to set input parameters (**Fig. S9.3**; left). We have established parameter ranges in the information windows, as well as error messages if the input parameters are outside the suitable range (**Fig. S9.3**; right).


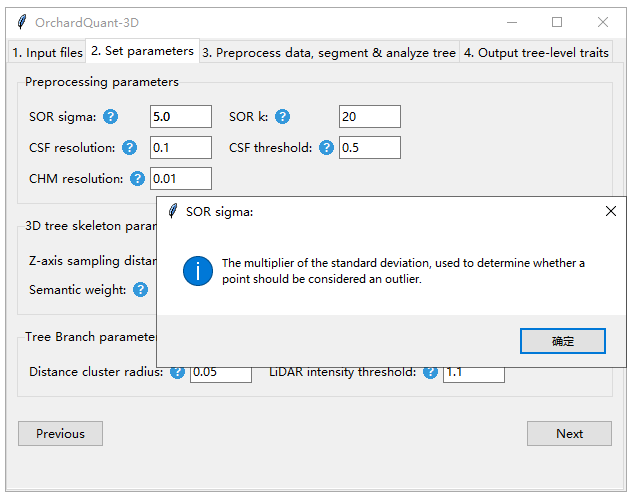

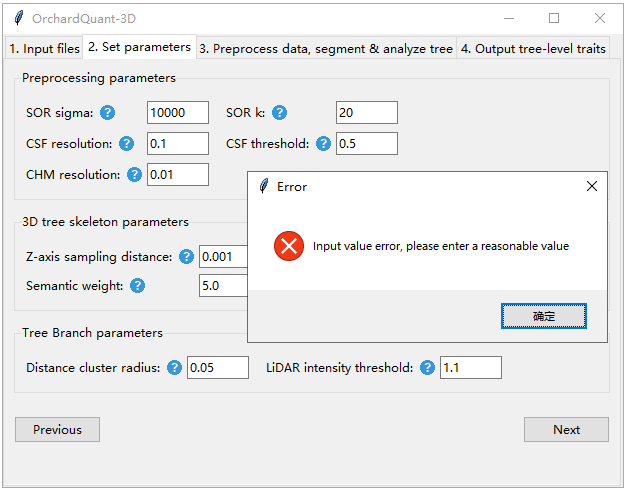


**Figure S9.3** Information icons and error messages in the OrchardQuant-3D GUI

*3. Preprocess data, segment & analyse trees*

Batch processing of 3D point clouds can be performed automatically (**Fig. S9.4**; left). By clicking the “Data preprocessing” option in the GUI, the input point clouds can be analysed to obtain above-ground 3D points, with a progress bar displayed. Then, users can click the “Tree segmentation” option to segment the above-ground tree point clouds to segment individual trees in the orchard. Finally, users can click the “3D skeletonization” option to derive 3D skeleton points of the fruit trees (**Fig. S9.** **4**; right).


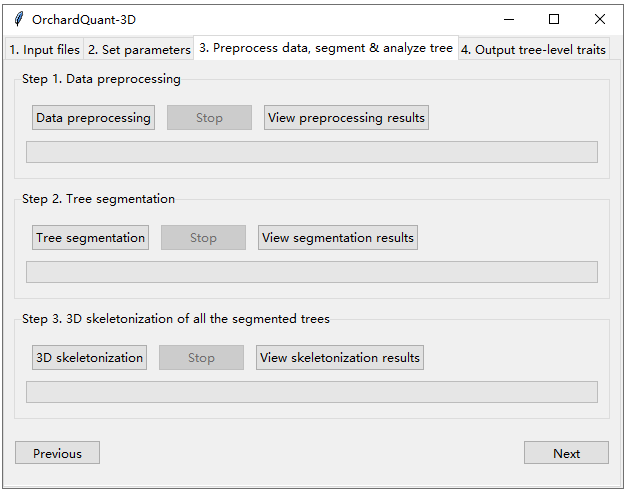

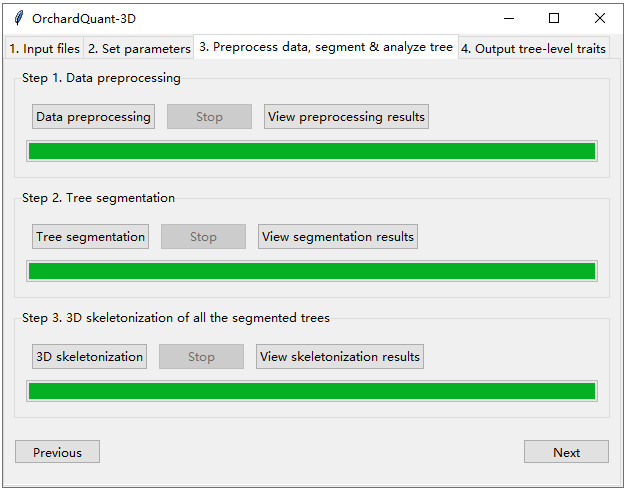


**Figure S9.4** The window of the OrchardQuant-3D GUI’s pre-processing, segmenting and analysing tree-level traits

Users can easily view the output files generated at each processing stage by clicking the “View preprocessing results”, “View segmentation results”, and “View skeletonization results” buttons in the GUI (**Fig. S9.5**). This function also allows users to evaluate the results of every step, thereby ensuring the successful execution of the entire workflow.


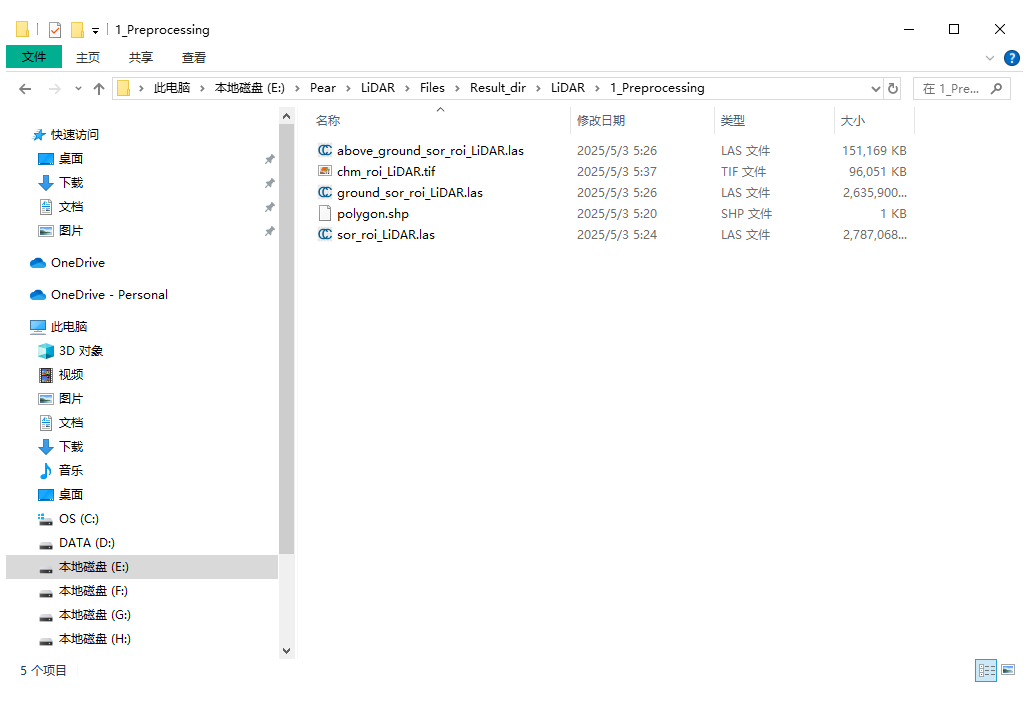

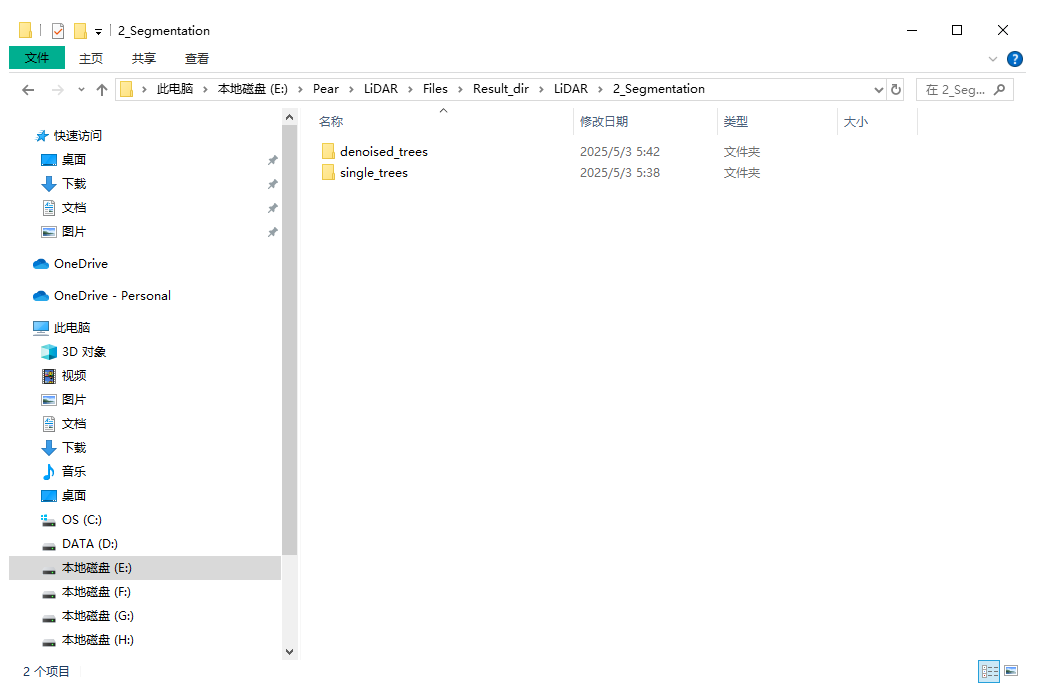

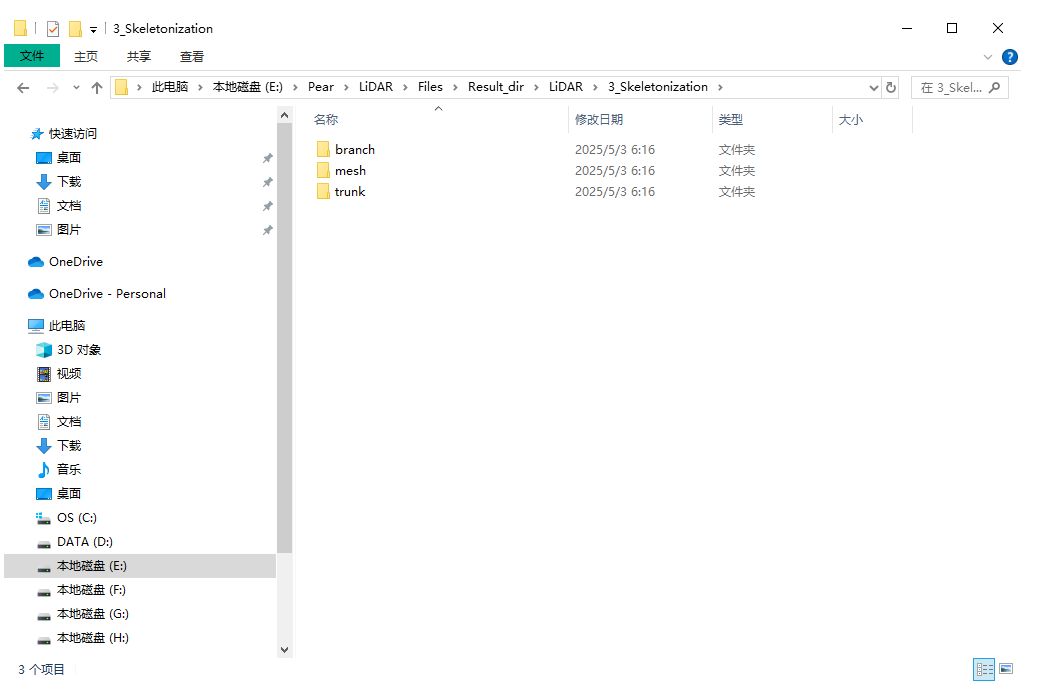


**Figure 5** Use the OrchardQuant-3D GUI to view results of preprocessing, tree segmentation and tree-level phenotypic analysis

*4. Output tree-level traits*

Based on the results obtained, the final section can be used to automate branch-level and tree-level trait analyses. By clicking “Quantify branch traits” in the GUI, branch characteristics can be measured. By clicking “Quantify crown traits”, tree-level canopy traits can be quantified and exported **(Fig. S9.6**).


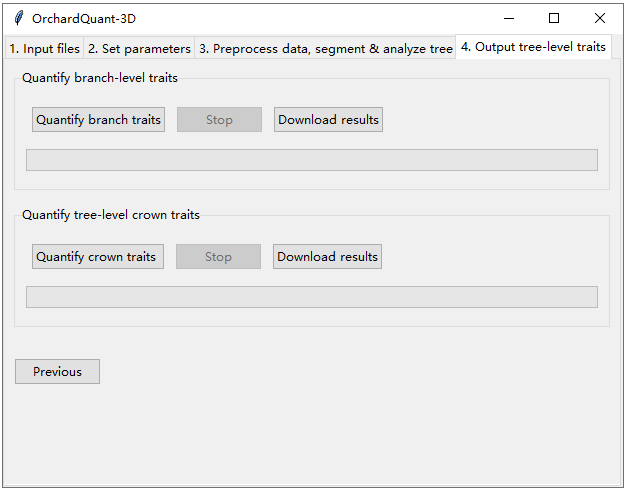

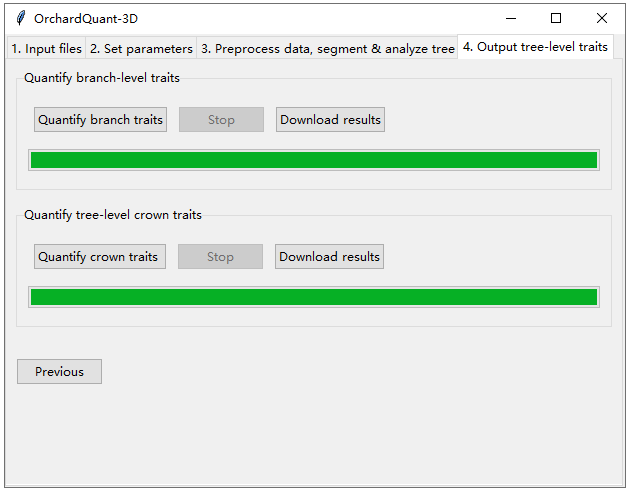


**Figure S9.6** The OrchardQuant-3D GUI for tree-level trait analysis

Finally, by clicking the “Download Results” button, users can download the processed results to a given folder, which saves skeleton point clouds generated during branch-level trait analysis (**Fig. S9.7**; left), and canopy-level traits (**Fig. S9.7**; right).


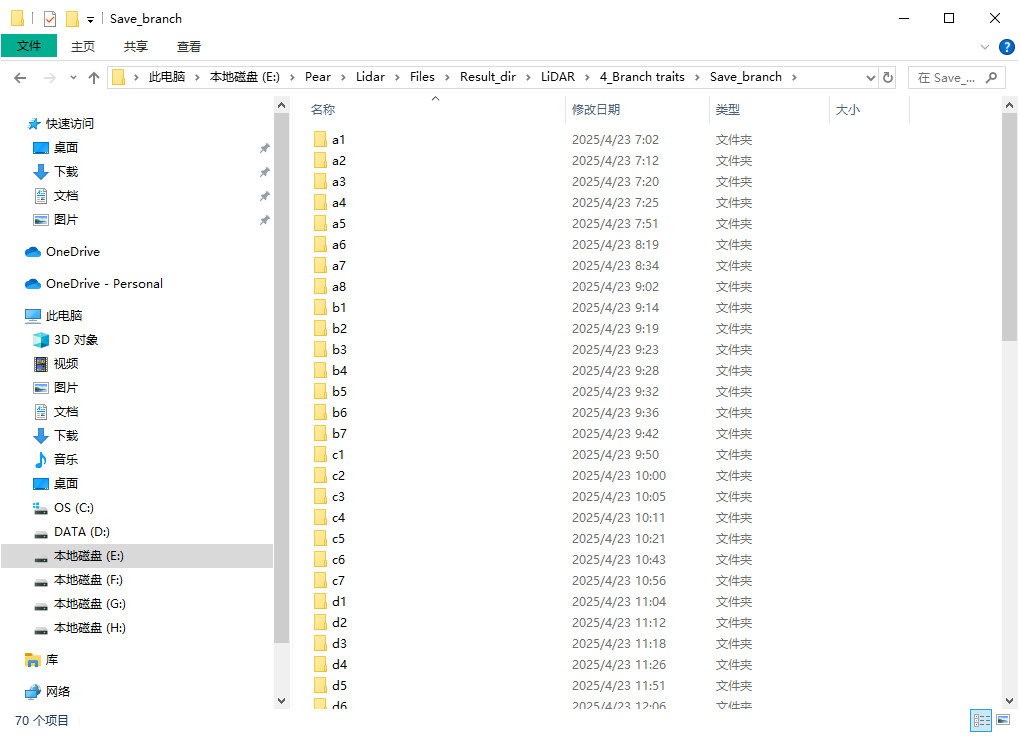

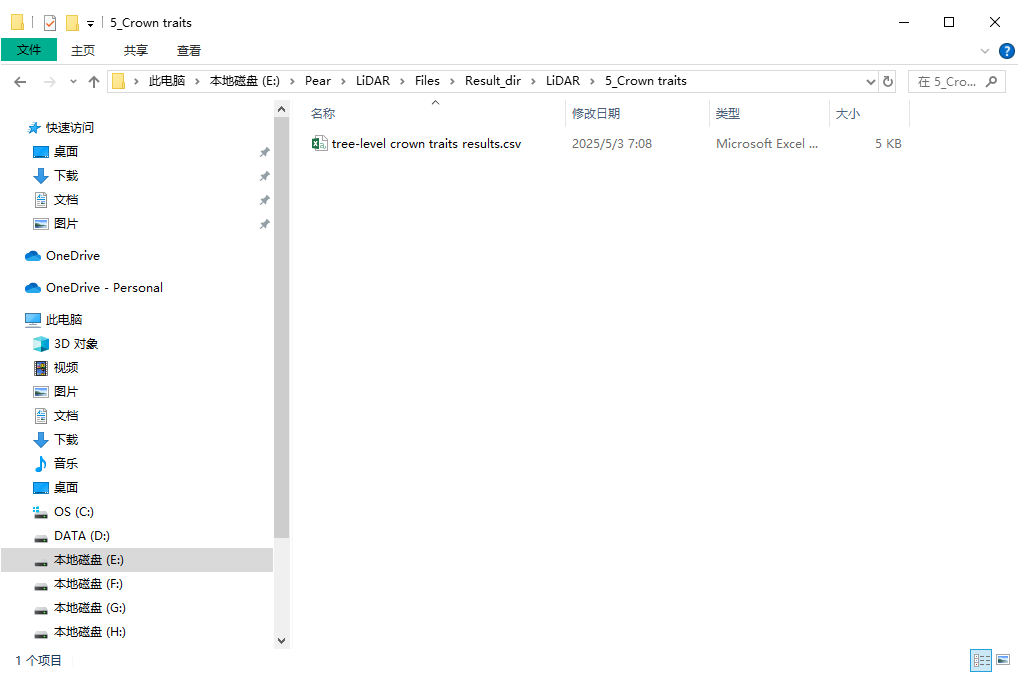


**Figure S9.7** The OrchardQuant-3D GUI for downloading analysis results

### Note S10 – Impacts of support structures when measuring tree height and crown volume.

According to our assessment, 3D points with or without support structures did not affect the accuracy of our tree height and crown volume measurements. When calculating the tree height trait, the median value of the top 1% 3D points’ z-axis values subtracted by the z-axis value of the bottom point of the tree trunk was used to quantify the tree height value for every tree in the orchard (**Fig. S10**; left). As for the crown volume trait (Num_Branch_), we employ a 3D convex hull method to compute this trait. We selected Tree1 (Row 1, Colome 1) for visualisation (**Fig. S10**; right). Since the support structures are embedded within tree branches, the measures of 3D convex hull (in m^3^) remain unchanged with or without supporting structures and thus did not affect the crown volume trait measurement.


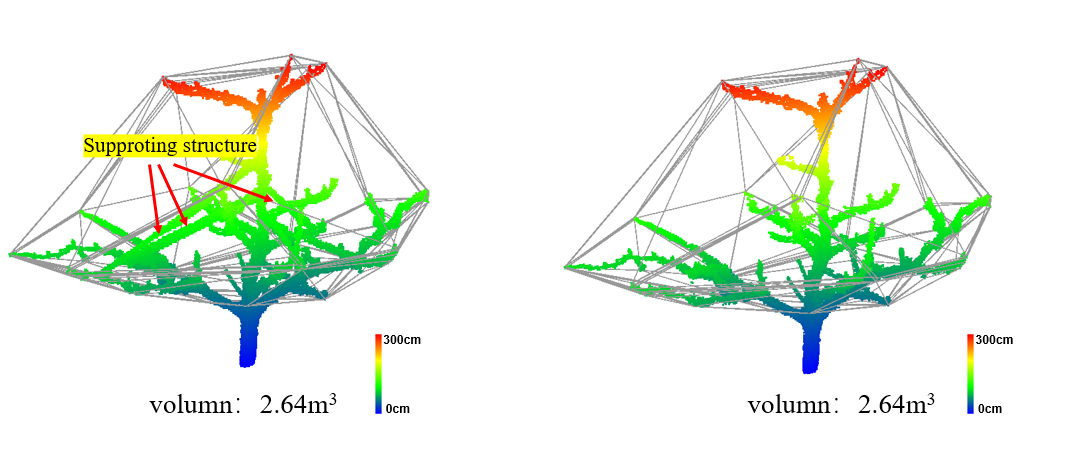


**Figure S10** Pear tree crown’s 3D convex hull with and without supporting structures

### Note S11 – Comparisons of different methods for quantifying tree branches.

Besides comparing key OrchardQuant-3D-derived traits against manual scoring, we also compared our pipeline with three representative methods, TreeQSM, AdTree, and AppleQSM, for the Num_Branch_ trait. To ensure meaningful comparisons, we used 3D points of Tree 38 (Row 5 and Column 3 in the orchard) with supporting structures removed. Manually, specialists scored 45 branches of the tree (**Fig. S11.1**; left). Default parameters published with the three methods were used in the following comparison.

*1. TreeQSM*

We downloaded the TreeQSM (Raumonen *et al.*, 2013) and applied it to analyse bud burst stage 3D tree points (https://github.com/InverseTampere/TreeQSM/blob/master/src/create_input.m). The TreeQSM scored 28 brances for Tree 38, which suggested a low accuracy in segmenting some parts of the 3D tree model (**Fig. S11.1**, right; see the red-coloured rectangle), indicating that the TreeQSM method might be less accurate for complex pear tree structures.


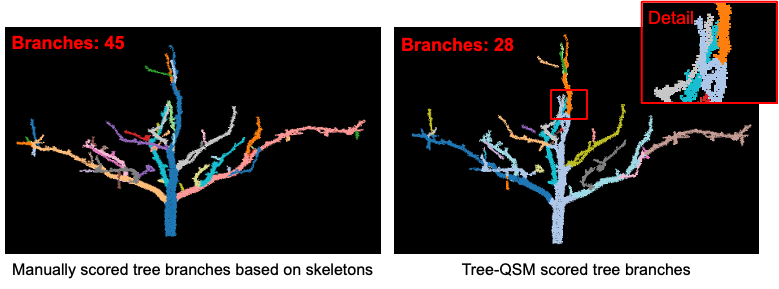


**Figure S11.1** The quantification of tree branches manually and using TreeQSM

*2. AdTree & AppleQSM*

Then, we used the AdTree (Du *et al.*, 2019) to analyse 3D point clouds of Tree 38, resulting in 5,494 branches due to many tiny pseudo branches wrongly reconstructed at tree branch connection and end points (**Fig. S11.2**; left). When testing the AppleQSM ,method (Qiu *et al.*, 2024)，we used the published source code from GitHub repository and recommended input parameters (https://github.com/suptimq/AppleQSM/tree/master/code/config). We found that the algorithm concluded 8 branches when analysing pear tree point clouds, which might be caused by different tree structures between apples and pears (**Fig. S11.2**; right).

**Figure S11.2** The quantification of tree branches using AdTree and AppleQSM

*3. The OrchardQuant-3D method*

Finally, we applied the OrchardQuant-3D to quantify branches for Tree 38, which identified 42 branches with only three small branches missing in the analysis (**Fig. S11.3**; left). Importantly, the method performed well when studying complex branch connection points analysis (**Fig. S11.3**; right). Also, it required limited manual adjustment of input parameters, making the method more robust across different tree types.

**Figure S11.3** The quantification of tree branches using OrchardQuant-3D

### Note S12 – Statistical analysis of tree-level canopy, floral, and fruit traits.

*1. 12 tree-level canopy and floral trait analysis*

Based on the data distribution (whether the data followed a normal distribution and homogeneity of variance), we determined whether the Bonferroni method (post-hoc multiple comparisons) or the non-parametric Kruskal-Wallis method should be used in the statistical analysis. The table below measures varietal differences between the four varieties in the orchard (^**^ *P* ≤ 0.01, ^***^ *P* ≤ 0.001).

| **Trait** | **Variety comparison** | ***P_value*** |
| --- | --- | --- |
| **Canopy surface area** | Cuiguan and Cuiyu | 0.091572 |
|  | Cuiguan and Xialu | 0.43234 |
|  | Cuiguan and Housui | 0.154611 |
|  | Cuiyu and Xialu | 1 |
|  | Cuiyu and Housui | 8.08E-05^***^ |
|  | Xialu and Housui | 0.001223^**^ |
| **Canopy volume** | Cuiguan and Cuiyu | 0.054714 |
|  | Cuiguan and Xialu | 0.233718 |
|  | Cuiguan and Housui | 0.123233 |
|  | Cuiyu and Xialu | 1 |
|  | Cuiyu and Housui | 2.51E-05^***^ |
|  | Xialu and Housui | 0.000319^***^ |
| **Canopy projection area** | Cuiguan and Cuiyu | 0.247135 |
|  | Cuiguan and Xialu | 1 |
|  | Cuiguan and Housui | 0.180336 |
|  | Cuiyu and Xialu | 1 |
|  | Cuiyu and Housui | 0.000491^***^ |
|  | Xialu and Housui | 0.011898^*^ |
| **Tree height** | Cuiguan and Cuiyu | 0.141049 |
|  | Cuiguan and Xialu | 0.012733^*^ |
|  | Cuiguan and Housui | 0.533067 |
|  | Cuiyu and Xialu | 1 |
|  | Cuiyu and Housui | 0.001268^**^ |
|  | Xialu and Housui | 5.97E-05^***^ |
| **Number of branches** | Cuiguan and Cuiyu | 0.130771 |
|  | Cuiguan and Xialu | 0.305896 |
|  | Cuiguan and Housui | 0.098971 |
|  | Cuiyu and Xialu | 1 |
|  | Cuiyu and Housui | 6.85E-05^***^ |
|  | Xialu and Housui | 0.000364^***^ |
| **Number of flower clusters in the lower canopy** | Cuiguan and Cuiyu | 0.013361^*^ |
|  | Cuiguan and Xialu | 0.013839^*^ |
|  | Cuiguan and Housui | 1 |
|  | Cuiyu and Xialu | 1 |
|  | Cuiyu and Housui | 0.001344^**^ |
|  | Xialu and Housui | 0.001465^**^ |
| **Number of flower clusters in the middle canopy** | Cuiguan and Cuiyu | 1 |
|  | Cuiguan and Xialu | 1 |
|  | Cuiguan and Housui | 0.2263 |
|  | Cuiyu and Xialu | 1 |
|  | Cuiyu and Housui | 0.019204^*^ |
|  | Xialu and Housui | 0.263899 |
| **Number of flower clusters in the upper canopy** | Cuiguan and Cuiyu | 0.002776^**^ |
|  | Cuiguan and Xialu | 0.003524^**^ |
|  | Cuiguan and Housui | 1 |
|  | Cuiyu and Xialu | 1 |
|  | Cuiyu and Housui | 9.86E-05^***^ |
|  | Xialu and Housui | 0.000139^***^ |
| **Flower clusters projection area** | Cuiguan and Cuiyu | 0.219959 |
|  | Cuiguan and Xialu | 0.261467 |
|  | Cuiguan and Housui | 0.128473 |
|  | Cuiyu and Xialu | 1 |
|  | Cuiyu and Housui | 0.00049^***^ |
|  | Xialu and Housui | 0.000764^***^ |
| **Flower clusters surface area** | Cuiguan and Cuiyu | 0.068546 |
|  | Cuiguan and Xialu | 0.124984 |
|  | Cuiguan and Housui | 0.207466 |
|  | Cuiyu and Xialu | 1 |
|  | Cuiyu and Housui | 8.61E-05^***^ |
|  | Xialu and Housui | 0.000264^***^ |
| **Flower clusters volume** | Cuiguan and Cuiyu | 0.0002^***^ |
|  | Cuiguan and Xialu | 0.001189^**^ |
|  | Cuiguan and Housui | 0.640697 |
|  | Cuiyu and Xialu | 1 |
|  | Cuiyu and Housui | 4.77E-07^***^ |
|  | Xialu and Housui | 4.65E-06^***^ |

*2. 1,104 tree-level floral and fruit traits analyses*

Correlation analyses were performed between tree-level apple cluster numbers and volumes on 2024/08/27 with tree-level blossom cluster numbers and volumes on 2024/04/24 using *Pearson*’s correlation coefficient (*r*), resulting in 0.677 for cluster volumes and 0.606 for cluster numbers.


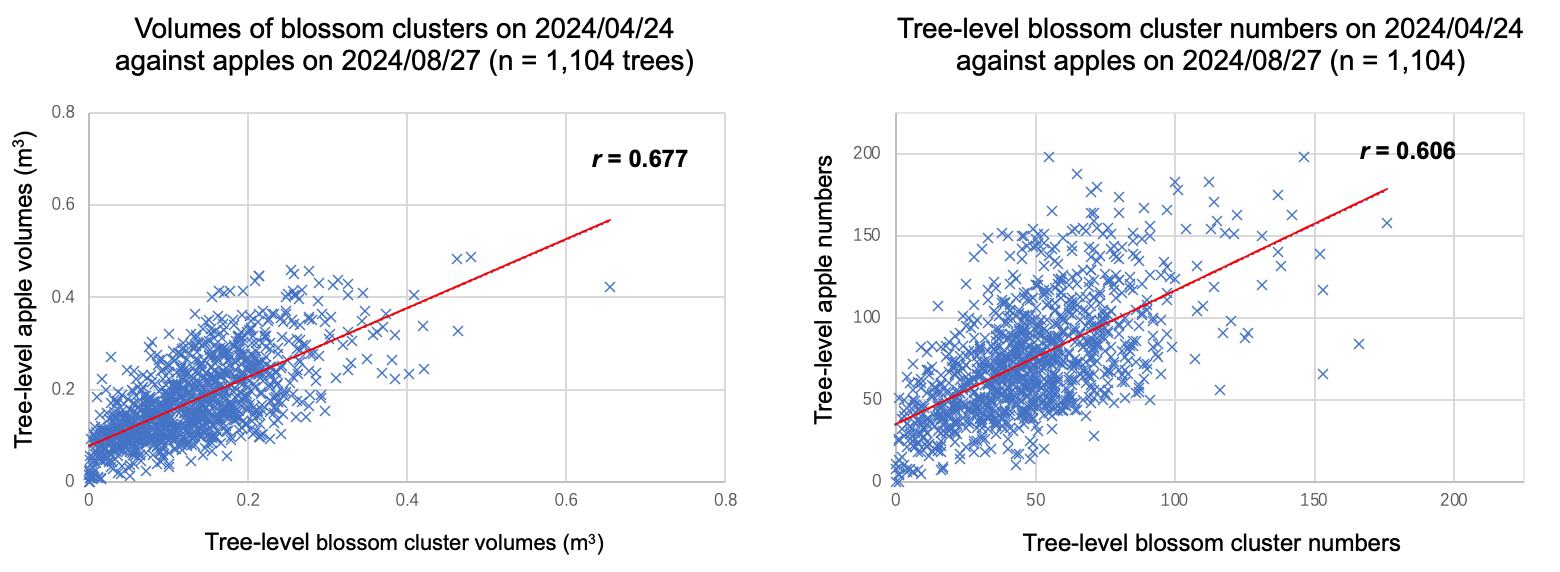


**Figure S12.1** *Pearson*’s correlation coefficient computed to compare tree-level blossom/apple numbers and volumes

# Supplementary Tables:

### Table S1. Crown volume (m^3^) of 70 pear trees in the orchard in 2023.

| **Col**  **Row** | **1** | **2** | **3** | **4** | **5** | **6** | **7** | **8** | **9** |
| --- | --- | --- | --- | --- | --- | --- | --- | --- | --- |
| **1** | 2.64 | 1.91 | 2.43 | 1.41 | 0.97 | 1.85 | 1.76 | 3.30 | 3.48 |
| **2** | 2.37 | 1.39 | 1.66 | 2.94 | 2.98 | 1.50 | 1.19 | 3.89 | 5.45 |
| **3** | 3.02 | 1.11 | 0.38 | 1.64 | 2.96 | 1.35 | 2.25 | 2.22 | 4.06 |
| **4** | 2.79 | 1.44 | N/A | 2.04 | 3.76 | 3.02 | 2.31 | 4.36 | 3.93 |
| **5** | 4.87 | 0.98 | 2.96 | 3.38 | 1.57 | 2.42 | 4.45 | 3.95 | 3.00 |
| **6** | 5.10 | 1.34 | 1.88 | 2.96 | 1.16 | 3.38 | 3.43 | 3.61 | 3.83 |
| **7** | 4.09 | 1.50 | 2.19 | 0.10 | 2.18 | 2.33 | 3.07 | 4.21 | 3.87 |
| **8** | 4.96 | N/A | 1.78 | 2.90 | 2.49 | 3.29 | 2.44 | 2.73 | 3.98 |

### Table S2. Surface area (m^2^) of 70 pear trees in the orchard in 2023.

| **Col**  **Row** | **1** | **2** | **3** | **4** | **5** | **6** | **7** | **8** | **9** |
| --- | --- | --- | --- | --- | --- | --- | --- | --- | --- |
| **1** | 10.49 | 8.66 | 10.53 | 6.97 | 5.72 | 8.37 | 8.21 | 12.24 | 12.70 |
| **2** | 9.69 | 7.07 | 7.91 | 12.16 | 11.22 | 7.55 | 6.85 | 13.12 | 16.83 |
| **3** | 11.74 | 6.30 | 3.16 | 7.98 | 11.19 | 6.72 | 9.68 | 9.18 | 14.00 |
| **4** | 11.01 | 7.24 | N/A | 9.37 | 13.52 | 11.60 | 10.22 | 14.86 | 14.14 |
| **5** | 15.76 | 5.70 | 11.71 | 12.72 | 7.68 | 9.89 | 15.57 | 13.50 | 11.62 |
| **6** | 15.85 | 6.73 | 8.24 | 11.16 | 6.30 | 12.30 | 12.83 | 13.06 | 12.71 |
| **7** | 14.15 | 6.95 | 9.20 | 1.48 | 9.56 | 9.96 | 12.32 | 14.10 | 13.61 |
| **8** | 15.45 | N/A | 8.04 | 11.80 | 9.89 | 12.39 | 10.45 | 10.31 | 13.81 |

### Table S3. Canopy diameter (cm) of 70 pear trees in the orchard in 2023.

| **Col**  **Row** | **1** | **2** | **3** | **4** | **5** | **6** | **7** | **8** | **9** |
| --- | --- | --- | --- | --- | --- | --- | --- | --- | --- |
| **1** | 286 | 240 | 316 | 209 | 218 | 235 | 267 | 287 | 292 |
| **2** | 236 | 246 | 209 | 355 | 229 | 226 | 256 | 277 | 332 |
| **3** | 295 | 217 | 168 | 260 | 252 | 205 | 303 | 233 | 287 |
| **4** | 299 | 260 | N/A | 310 | 327 | 299 | 326 | 336 | 309 |
| **5** | 335 | 209 | 346 | 304 | 232 | 254 | 385 | 296 | 305 |
| **6** | 327 | 198 | 199 | 302 | 192 | 294 | 310 | 318 | 254 |
| **7** | 338 | 181 | 233 | 111 | 263 | 265 | 303 | 310 | 350 |
| **8** | 283 | N/A | 222 | 339 | 232 | 251 | 288 | 260 | 299 |

### Table S4. Canopy projected area (m^2^) of 70 pear trees in the orchard in 2023.

| **Col**  **Row** | **1** | **2** | **3** | **4** | **5** | **6** | **7** | **8** | **9** |
| --- | --- | --- | --- | --- | --- | --- | --- | --- | --- |
| **1** | 3.04 | 2.51 | 3.48 | 1.82 | 1.68 | 2.45 | 2.41 | 3.41 | 3.76 |
| **2** | 2.66 | 1.79 | 2.34 | 4.18 | 2.49 | 2.38 | 1.92 | 3.52 | 5.49 |
| **3** | 4.10 | 1.41 | 0.85 | 2.36 | 2.79 | 1.84 | 3.04 | 2.65 | 4.54 |
| **4** | 3.32 | 2.07 | N/A | 3.10 | 4.29 | 3.52 | 3.02 | 4.47 | 4.70 |
| **5** | 4.53 | 1.56 | 3.94 | 4.02 | 1.96 | 2.86 | 4.23 | 4.41 | 3.60 |
| **6** | 4.98 | 1.91 | 2.32 | 3.55 | 1.71 | 3.83 | 4.21 | 4.39 | 3.39 |
| **7** | 4.45 | 1.91 | 3.00 | 0.28 | 2.50 | 3.33 | 4.41 | 4.28 | 4.12 |
| **8** | 4.29 | N/A | 2.16 | 3.75 | 2.90 | 3.09 | 3.36 | 2.79 | 3.91 |

### Table S5. The number of flower clusters of 70 pear trees in the orchard

| **Col**  **Row** | **1** | **2** | **3** | **4** | **5** | **6** | **7** | **8** | **9** |
| --- | --- | --- | --- | --- | --- | --- | --- | --- | --- |
| **1** | 114 | 66 | 87 | 73 | 56 | 95 | 92 | 110 | 135 |
| **2** | 94 | 71 | 98 | 103 | 159 | 69 | 72 | 161 | 166 |
| **3** | 91 | 49 | 33 | 78 | 121 | 87 | 86 | 115 | 116 |
| **4** | 91 | 87 | N/A | 98 | 118 | 113 | 101 | 143 | 122 |
| **5** | 134 | 44 | 107 | 127 | 58 | 91 | 133 | 139 | 149 |
| **6** | 154 | 78 | 105 | 114 | 67 | 111 | 107 | 141 | 138 |
| **7** | 127 | 67 | 86 | 17 | 114 | 107 | 122 | 187 | 132 |
| **8** | 167 | N/A | 55 | 108 | 124 | 129 | 93 | 91 | 133 |

### Table S6. The volume of flower clusters of 70 pear trees in the orchard (m^3^)

| **Col**  **Row** | **1** | **2** | **3** | **4** | **5** | **6** | **7** | **8** | **9** |
| --- | --- | --- | --- | --- | --- | --- | --- | --- | --- |
| **1** | 0.44 | 0.21 | 0.28 | 0.21 | 0.23 | 0.41 | 0.27 | 0.51 | 0.53 |
| **2** | 0.34 | 0.29 | 0.34 | 0.33 | 0.64 | 0.24 | 0.22 | 0.65 | 0.71 |
| **3** | 0.40 | 0.16 | 0.11 | 0.23 | 0.59 | 0.32 | 0.27 | 0.46 | 0.58 |
| **4** | 0.37 | 0.30 | N/A | 0.36 | 0.79 | 0.46 | 0.30 | 0.69 | 0.55 |
| **5** | 0.53 | 0.18 | 0.36 | 0.37 | 0.19 | 0.54 | 0.39 | 0.64 | 0.54 |
| **6** | 0.54 | 0.28 | 0.36 | 0.39 | 0.17 | 0.52 | 0.40 | 0.54 | 0.52 |
| **7** | 0.55 | 0.18 | 0.27 | 0.03 | 0.53 | 0.52 | 0.38 | 0.66 | 0.60 |
| **8** | 0.53 | N/A | 0.14 | 0.33 | 0.45 | 0.62 | 0.36 | 0.38 | 0.50 |

### Table S7. Surface area of flower clusters of 70 pear trees in the orchard (m^2^)

| **Col**  **Row** | **1** | **2** | **3** | **4** | **5** | **6** | **7** | **8** | **9** |
| --- | --- | --- | --- | --- | --- | --- | --- | --- | --- |
| **1** | 9.86 | 8.17 | 9.24 | 5.68 | 5.70 | 8.29 | 7.88 | 12.09 | 12.21 |
| **2** | 8.98 | 6.92 | 7.65 | 10.57 | 10.99 | 7.31 | 6.50 | 12.88 | 15.94 |
| **3** | 10.53 | 5.24 | 2.66 | 7.12 | 10.25 | 6.67 | 8.47 | 8.32 | 13.32 |
| **4** | 9.80 | 6.98 | N/A | 8.50 | 13.43 | 10.79 | 9.73 | 14.03 | 13.35 |
| **5** | 14.77 | 5.39 | 10.79 | 11.23 | 6.10 | 9.98 | 14.45 | 12.72 | 10.91 |
| **6** | 14.33 | 6.13 | 7.53 | 10.00 | 5.61 | 10.99 | 12.52 | 12.07 | 12.12 |
| **7** | 13.06 | 6.24 | 8.16 | 0.96 | 9.34 | 9.82 | 11.23 | 13.27 | 13.44 |
| **8** | 16.08 | N/A | 5.76 | 10.60 | 8.75 | 11.20 | 10.16 | 9.51 | 12.07 |

### Table S8. Projection area of flower clusters of 70 pear trees in the orchard (m^2^)

| **Col**  **Row** | **1** | **2** | **3** | **4** | **5** | **6** | **7** | **8** | **9** |
| --- | --- | --- | --- | --- | --- | --- | --- | --- | --- |
| **1** | 2.72 | 2.30 | 2.98 | 1.57 | 1.66 | 2.34 | 2.36 | 3.28 | 3.49 |
| **2** | 2.51 | 1.76 | 2.30 | 3.46 | 2.24 | 2.22 | 1.99 | 3.38 | 5.18 |
| **3** | 3.71 | 1.07 | 0.82 | 2.13 | 2.56 | 1.76 | 2.60 | 2.43 | 4.14 |
| **4** | 2.90 | 2.09 | N/A | 2.76 | 4.08 | 3.15 | 2.87 | 4.08 | 4.41 |
| **5** | 4.11 | 1.54 | 3.55 | 3.45 | 1.66 | 2.86 | 4.50 | 4.10 | 3.30 |
| **6** | 4.52 | 1.58 | 2.23 | 3.27 | 1.66 | 3.51 | 3.93 | 4.04 | 3.17 |
| **7** | 4.14 | 1.67 | 2.52 | 0.22 | 2.46 | 3.16 | 4.03 | 4.00 | 4.03 |
| **8** | 4.50 | N/A | 1.32 | 3.44 | 2.38 | 2.95 | 3.23 | 2.64 | 3.74 |

### Table S9. Branch-level blossom cluster analysis with both geo-positions and trait analysis

| **Cluster ID** | **X coordinate** | **Y coordinate** | **Z coordinate** | **No of 3D points** | **Surface area (cm^2^)** | **Volume (cm^3^)** |
| --- | --- | --- | --- | --- | --- | --- |
| **1** | -2.18521508e+06 | 1.38576170e+07 | 4.97122444e+01 | 283 | 201 | 221 |
| **2** | -2.18521507e+06 | 1.38576171e+07 | 4.96548467e+01 | 184 | 213 | 180 |
| **3** | -2.18521498e+06 | 1.38576171e+07 | 4.96014315e+01 | 475 | 383 | 562 |
| **4** | -2.18521500e+06 | 1.38576172e+07 | 4.95461175e+01 | 65 | 36 | 14 |
| **5** | -2.18521513e+06 | 1.38576172e+07 | 4.94788267e+01 | 454 | 333 | 430 |
| **6** | -2.18521508e+06 | 1.38576173e+07 | 4.92956855e+01 | 161 | 137 | 112 |
| **7** | -2.18521521e+06 | 1.38576173e+07 | 4.92706445e+01 | 71 | 58 | 28 |
| **8** | -2.18521514e+06 | 1.38576174e+07 | 4.92050810e+01 | 80 | 70 | 35 |
| **9** | -2.18521524e+06 | 1.38576176e+07 | 4.92655889e+01 | 568 | 416 | 674 |
| **10** | -2.18521529e+06 | 1.38576174e+07 | 4.92376703e+01 | 288 | 219 | 198 |
| **11** | -2.18521537e+06 | 1.38576176e+07 | 4.91384171e+01 | 70 | 65 | 35 |

### Table S10. Tree-level canopy volume (m³) measured in the apple orchard on 16^th^ April 2024.

| **Col**  **Row** | **1** | **2** | **3** | **4** | **5** | **6** | **7** | **8** | **9** | **10** | **11** | **12** | **13** | **14** |
| --- | --- | --- | --- | --- | --- | --- | --- | --- | --- | --- | --- | --- | --- | --- |
| **80** | 1.27 | 1.64 | 1.77 | 1.64 | 1.93 | 1.73 | 2.05 | 0.96 | 1.97 | 1.87 | 1.14 | 1.36 | 1.46 | 1.16 |
| **79** | 1.72 | 1.61 | 1.69 | 1.53 | 1.54 | 1.97 | 1.47 | 1.33 | 2.09 | 1.83 | 1.51 | 1.26 | 1.80 | 1.40 |
| **78** | 1.19 | 1.84 | 1.75 | 1.94 | 1.72 | 2.05 | 1.55 | 1.41 | 1.94 | 1.64 | 1.41 | 1.11 | 1.34 | 1.28 |
| **77** | 1.72 | 2.35 | 2.67 | 1.55 | 1.98 | 2.21 | 2.05 | 2.51 | 2.55 | 2.40 | 2.15 | 2.13 | 1.72 | 1.38 |
| **76** | 2.24 | 2.61 | 2.25 | 1.77 | 1.12 | 2.70 | 2.50 | 2.03 | 2.66 | 2.62 | 2.24 | 2.07 | 2.03 | 1.51 |
| **75** | 1.98 | 2.17 | 2.20 | 2.21 | 1.79 | 2.75 | 2.42 | 2.44 | 2.56 | 1.72 | 2.42 | 2.10 | 2.26 | 1.01 |
| **74** | 2.00 | 2.01 | 1.84 | 1.73 | 1.51 | 2.12 | 1.35 | 2.17 | 2.28 | 1.68 | 1.87 | 1.90 | 2.18 | 1.93 |
| **73** | 1.96 | 2.00 | 2.18 | 1.43 | 1.86 | 2.40 | N/A | 2.07 | 2.48 | 2.13 | 0.77 | 1.64 | 2.36 | 1.36 |
| **72** | 1.52 | 2.57 | 2.00 | 1.31 | 1.48 | 2.53 | 0.51 | 1.82 | 2.15 | 2.26 | 1.23 | 1.79 | 1.83 | 1.59 |
| **71** | 1.37 | 2.62 | 2.13 | 1.65 | 1.61 | 2.94 | 1.36 | 1.64 | 2.84 | 2.02 | 1.98 | 1.81 | 2.06 | 1.90 |
| **70** | 1.55 | 1.71 | 2.22 | 1.84 | N/A | 2.47 | 1.74 | 1.95 | 2.39 | 2.39 | 1.16 | 2.01 | 1.75 | 2.13 |
| **69** | 1.91 | 1.73 | 2.17 | N/A | 1.10 | 2.60 | 1.61 | 2.20 | 2.76 | 2.18 | 1.61 | 1.78 | 1.90 | 2.33 |
| **68** | 2.25 | 1.90 | 2.19 | 1.05 | 2.79 | 2.56 | 2.19 | 2.61 | 2.93 | 2.14 | 1.81 | 1.86 | 1.98 | 1.29 |
| **67** | 1.50 | 2.29 | 2.37 | 1.88 | 2.33 | 2.81 | 2.30 | 1.57 | 2.59 | 2.35 | 2.11 | 2.18 | 1.73 | 1.81 |
| **66** | 1.68 | 2.38 | 2.28 | 1.61 | 2.05 | 2.79 | 2.51 | 1.85 | 2.80 | 2.70 | 2.30 | 1.52 | 2.14 | 2.04 |
| **65** | 2.17 | 2.36 | 2.03 | 1.89 | 1.94 | 2.41 | 2.36 | 1.97 | 2.67 | 2.09 | 1.60 | 1.92 | 1.75 | 1.62 |
| **64** | 1.91 | 2.06 | 2.12 | 2.04 | 1.76 | 2.43 | 2.03 | 2.22 | 2.75 | 1.86 | 1.68 | 2.22 | 2.05 | 1.56 |
| **63** | 1.81 | 2.35 | 2.71 | 2.53 | 1.80 | 2.58 | 1.91 | 2.21 | 2.75 | 1.70 | 1.81 | 2.04 | 2.24 | 2.18 |
| **62** | 1.88 | 2.30 | 2.61 | 2.94 | 1.79 | 2.31 | 0.95 | 2.25 | 2.56 | 1.75 | 1.67 | 2.13 | 2.23 | 1.72 |
| **61** | 2.27 | 2.35 | 2.22 | 2.68 | N/A | 1.83 | 1.57 | 1.28 | 2.53 | 2.14 | 1.93 | 2.12 | 1.94 | 1.95 |
| **60** | 1.57 | 2.16 | 2.32 | 2.69 | 1.01 | 2.80 | N/A | 1.63 | 2.51 | 2.12 | 2.38 | 2.07 | 1.76 | 1.36 |
| **59** | 1.96 | 2.91 | 2.00 | 2.53 | 2.18 | 3.32 | 1.93 | 2.44 | 2.82 | 2.54 | 2.08 | 2.21 | 2.04 | 1.77 |
| **58** | 1.82 | 2.43 | 2.24 | 2.05 | 2.37 | 2.83 | 2.12 | 2.01 | 2.53 | 2.61 | 1.90 | 2.12 | 2.35 | 1.66 |
| **57** | 2.08 | 1.58 | 2.34 | 1.40 | 2.13 | 3.00 | 2.12 | 2.52 | 2.44 | 2.19 | 1.94 | 2.04 | 2.08 | 1.33 |
| **56** | 2.32 | 1.90 | 2.39 | 1.44 | 2.45 | 2.89 | 2.36 | 2.43 | 2.39 | 1.75 | 1.88 | 1.52 | 1.78 | 1.71 |
| **55** | 2.07 | 2.55 | 2.32 | 1.89 | 2.61 | 2.70 | 1.66 | 1.74 | 2.66 | 1.82 | 2.38 | 1.96 | 1.72 | 1.62 |
| **54** | 1.24 | 2.48 | 2.07 | 1.71 | 2.67 | 2.86 | 1.94 | 1.58 | 2.76 | 1.82 | 2.55 | 1.95 | 1.62 | 1.54 |
| **53** | 1.63 | 1.97 | 2.28 | 1.67 | 2.03 | 2.55 | 1.95 | 1.86 | 2.53 | 2.38 | 1.83 | 1.90 | 1.63 | 1.34 |
| **52** | 2.75 | 2.22 | 2.67 | 0.91 | 1.43 | 2.96 | 1.10 | 1.97 | 2.69 | 2.38 | 2.06 | 2.04 | 1.80 | 1.80 |
| **51** | 1.67 | 2.59 | 2.68 | N/A | 1.36 | 2.98 | 2.19 | 1.50 | 2.67 | 2.24 | 1.74 | 2.27 | 1.88 | 1.73 |
| **50** | 1.76 | 2.37 | 2.56 | 1.80 | 1.78 | 2.85 | 1.21 | 1.15 | 2.40 | 2.08 | 2.06 | 1.74 | 1.73 | 0.95 |
| **49** | 1.62 | 2.15 | 2.28 | 2.21 | 1.20 | 2.03 | 1.36 | 0.81 | 2.65 | 1.77 | 1.94 | 2.17 | 2.24 | 1.32 |
| **48** | 1.29 | 1.65 | 2.03 | 2.42 | 0.97 | 2.16 | 0.97 | 1.05 | 2.83 | 1.93 | 1.56 | 1.94 | 1.32 | 2.02 |
| **47** | 2.43 | 2.35 | 2.68 | 1.95 | 1.80 | 2.69 | 1.28 | 1.25 | 2.91 | 1.64 | 1.41 | 1.73 | 1.48 | 1.96 |
| **46** | 1.98 | 2.44 | 2.00 | 1.73 | 0.83 | 3.02 | 1.72 | 1.66 | 2.30 | 1.87 | 1.80 | 1.78 | 1.48 | 1.41 |
| **45** | 2.44 | 2.63 | 1.46 | 2.02 | 0.99 | 2.97 | 2.10 | 1.48 | 2.42 | 1.63 | 1.68 | 2.27 | 1.48 | 1.92 |
| **44** | 2.24 | 2.66 | 2.28 | 2.38 | 1.72 | 2.08 | 1.16 | 1.68 | 2.16 | 2.35 | 1.66 | 2.83 | 1.72 | 1.85 |
| **43** | 2.71 | 2.79 | 2.16 | 2.61 | 1.81 | 2.55 | 1.42 | 0.97 | 2.74 | 2.77 | 1.69 | 2.34 | 1.82 | 1.78 |
| **42** | 2.98 | 2.51 | 1.93 | 2.06 | 1.51 | 2.33 | 2.01 | 1.58 | 2.95 | 1.87 | 1.61 | 2.05 | 1.84 | 1.44 |
| **41** | 2.74 | 2.69 | 1.83 | 2.36 | 1.61 | 2.39 | 2.23 | 1.71 | 2.53 | 1.94 | 1.18 | 2.11 | 1.67 | 1.01 |
| **40** | 2.53 | 2.93 | 2.40 | 2.60 | 1.86 | 2.29 | 1.95 | 1.59 | 2.39 | 1.87 | N/A | 2.27 | 1.55 | 1.63 |
| **39** | 2.07 | 2.72 | 2.17 | 2.27 | 2.32 | 2.31 | 2.13 | 1.96 | 2.14 | 2.00 | 1.42 | 2.10 | 1.46 | 1.47 |
| **38** | 2.08 | 2.24 | 2.23 | 2.06 | 2.35 | 1.83 | 1.94 | 1.91 | 2.32 | 1.30 | N/A | 2.53 | 1.72 | 1.98 |
| **37** | 2.09 | 2.49 | 1.82 | 2.50 | 2.31 | 2.36 | 2.41 | 1.93 | 1.85 | 1.91 | 1.55 | 2.54 | 2.09 | 1.72 |
| **36** | 2.39 | 2.29 | 2.40 | 2.59 | 2.37 | 2.53 | 2.17 | 1.93 | 3.06 | 2.38 | 1.90 | 2.33 | 1.79 | 2.16 |
| **35** | 2.74 | 2.77 | 2.70 | 2.68 | 2.44 | 2.55 | 2.74 | 2.20 | 3.13 | 1.72 | 1.76 | 2.48 | 2.13 | 1.95 |
| **34** | 2.51 | 1.72 | 2.74 | 1.83 | 2.36 | 2.18 | 2.35 | 1.87 | 2.76 | 2.01 | 1.53 | 2.09 | 1.57 | 1.66 |
| **33** | 2.61 | 2.16 | 2.77 | 1.46 | 1.84 | 2.47 | 2.53 | 2.28 | 2.42 | 1.86 | 2.01 | 1.84 | 0.33 | 1.88 |
| **32** | 2.68 | 2.53 | 2.97 | 2.36 | 2.21 | 2.29 | 2.23 | 2.06 | 3.03 | 1.49 | 1.84 | 2.17 | 1.65 | 2.25 |
| **31** | 2.67 | 1.75 | 2.91 | 2.53 | 2.25 | 2.86 | 2.36 | 2.01 | 2.68 | 1.40 | 2.02 | 2.25 | 0.82 | 1.80 |
| **30** | 2.14 | 2.23 | 2.71 | 1.89 | 1.88 | 2.58 | 2.34 | N/A | 2.69 | 1.20 | 1.94 | 1.85 | 0.59 | 1.21 |
| **29** | 2.45 | 2.14 | 2.22 | 2.12 | 1.87 | 2.73 | 1.62 | 1.32 | 2.64 | 1.90 | 2.31 | 2.82 | 1.14 | 2.03 |
| **28** | 2.06 | 2.34 | 2.49 | 2.20 | 1.77 | 2.85 | 1.55 | 2.22 | 2.46 | 2.35 | 2.54 | 2.41 | 1.56 | 1.98 |
| **27** | 1.86 | 2.89 | 2.75 | 2.28 | 1.60 | 2.56 | 2.00 | 2.42 | 2.76 | 2.49 | 1.59 | 2.26 | 2.14 | 1.91 |
| **26** | 1.81 | 2.59 | 2.94 | 2.20 | 1.96 | 2.71 | 2.59 | 2.37 | 2.36 | 2.19 | 1.68 | 2.46 | 2.22 | 2.05 |
| **25** | 1.72 | 1.79 | 2.82 | 1.60 | 1.79 | 2.60 | 1.68 | 2.50 | 2.11 | 2.30 | 2.01 | 2.52 | 1.99 | 1.60 |
| **24** | 1.86 | 2.17 | 2.89 | 2.22 | 1.95 | 2.66 | 1.62 | 2.83 | 2.75 | 2.63 | 2.17 | 2.48 | 1.25 | 2.09 |
| **23** | 1.96 | 2.24 | 2.58 | 2.20 | 1.93 | 2.55 | N/A | 2.20 | 2.70 | 2.02 | 1.54 | 2.67 | 1.45 | 1.85 |
| **22** | 1.77 | N/A | 2.55 | 1.86 | 1.97 | 3.06 | N/A | 2.56 | 2.77 | 2.09 | 1.61 | 2.69 | 0.82 | 1.75 |
| **21** | 2.06 | 2.08 | 2.45 | 1.91 | 2.32 | 2.67 | N/A | 2.12 | 1.73 | 1.41 | 2.38 | 2.77 | 1.01 | 1.89 |
| **20** | 2.23 | 2.44 | 2.47 | 2.23 | 2.40 | 2.99 | N/A | 2.07 | 1.59 | 0.75 | 2.64 | 2.63 | 1.75 | 2.04 |
| **19** | 2.66 | 2.99 | 2.75 | 2.43 | 2.10 | 3.30 | N/A | 2.30 | 2.75 | 2.21 | 2.65 | 2.81 | 1.46 | 1.75 |
| **18** | 2.51 | 2.34 | 2.61 | 3.26 | 2.13 | 2.55 | N/A | 1.86 | 3.26 | 1.78 | 2.13 | 2.29 | 1.08 | 1.82 |
| **17** | 2.20 | 2.28 | 2.73 | 2.25 | 1.81 | 2.68 | 2.01 | 1.62 | 2.84 | 1.77 | 2.26 | 2.53 | 1.21 | 1.53 |
| **16** | 2.44 | 2.40 | 2.65 | 1.81 | 1.92 | 2.59 | 2.45 | 2.31 | 2.98 | 1.89 | 2.31 | 2.75 | 1.76 | 1.84 |
| **15** | 1.85 | 2.32 | 2.15 | 1.39 | 2.02 | 2.64 | 3.00 | 2.18 | 2.77 | 1.87 | 2.66 | 2.41 | 2.30 | 2.61 |
| **14** | 1.91 | 2.17 | 1.98 | 1.84 | 2.30 | 2.35 | 3.02 | 2.31 | 2.93 | 1.74 | 2.15 | 2.22 | 1.94 | 2.58 |
| **13** | 2.28 | 1.78 | 1.87 | 1.94 | 1.69 | 3.15 | 2.13 | 2.46 | 3.11 | 1.73 | 2.06 | 2.06 | 2.17 | 2.77 |
| **12** | 2.18 | 2.25 | 1.69 | 1.54 | 2.05 | 3.28 | 2.32 | 2.33 | 2.93 | 2.20 | 2.26 | 2.38 | 2.26 | 2.34 |
| **11** | 2.22 | 2.06 | 1.73 | 1.16 | 1.38 | 2.98 | 2.86 | 2.65 | 2.53 | 2.59 | 2.37 | 2.03 | 1.39 | 1.96 |
| **10** | 2.29 | 1.89 | 2.77 | 2.37 | 1.69 | 2.43 | 3.07 | 2.03 | 2.66 | 2.08 | 2.45 | 1.29 | 2.07 | 2.24 |
| **9** | 2.62 | 0.98 | 2.78 | 2.68 | 1.98 | 2.75 | 2.47 | 1.47 | 2.73 | 2.33 | 2.69 | 1.88 | 1.97 | 1.63 |
| **8** | 2.47 | 2.29 | 2.54 | 2.55 | 1.97 | 3.16 | 2.04 | 1.95 | 2.58 | 2.18 | 2.25 | 2.31 | 1.68 | 1.74 |
| **7** | 2.30 | 2.18 | 2.32 | 2.57 | 1.73 | 2.96 | 1.57 | 1.98 | 2.76 | 2.53 | 2.26 | 2.21 | 1.75 | 1.79 |
| **6** | 2.00 | 2.11 | 2.14 | 1.90 | 1.69 | 2.59 | 2.05 | 1.89 | 2.80 | 2.03 | 2.19 | 2.52 | 2.10 | 1.88 |
| **5** | 2.53 | 2.51 | 2.17 | 2.21 | 1.88 | 2.71 | 1.99 | 1.79 | 3.06 | 2.11 | 2.57 | 2.36 | 1.80 | 1.54 |
| **4** | 1.82 | 1.89 | 1.88 | 2.01 | 1.70 | 2.59 | 1.82 | 1.85 | 2.75 | 1.71 | 2.00 | 1.54 | 1.32 | 1.87 |
| **3** | 1.64 | 0.78 | 1.75 | 1.37 | 1.37 | 1.63 | 1.43 | 1.47 | 2.02 | 1.24 | 1.26 | 1.58 | 1.35 | 1.83 |
| **2** | 1.06 | 0.77 | 1.67 | 1.45 | 1.39 | 1.81 | 0.85 | 1.35 | 1.80 | 0.62 | 1.48 | 1.38 | 1.47 | 1.37 |
| **1** | 1.29 | 1.15 | 1.48 | 1.01 | 1.37 | 1.69 | 0.71 | 1.34 | 1.52 | 1.21 | 1.58 | 1.14 | 1.73 | 1.32 |

### Table S11. Tree-level canopy volume (m³) measured in the apple orchard on 24^th^ April 2024.

| **Col**  **Row** | **1** | **2** | **3** | **4** | **5** | **6** | **7** | **8** | **9** | **10** | **11** | **12** | **13** | **14** |
| --- | --- | --- | --- | --- | --- | --- | --- | --- | --- | --- | --- | --- | --- | --- |
| **80** | 1.64 | 1.76 | 1.68 | 1.91 | 2.19 | 1.99 | 2.23 | 1.17 | 2.10 | 2.06 | 1.18 | 1.48 | 1.66 | 1.52 |
| **79** | 2.24 | 1.71 | 1.73 | 1.82 | 1.31 | 2.42 | 1.74 | 1.72 | 2.09 | 1.56 | 1.74 | 1.68 | 2.13 | 1.76 |
| **78** | 1.48 | 1.92 | 2.18 | 2.38 | 2.58 | 2.39 | 1.85 | 1.61 | 2.37 | 1.56 | 2.00 | 1.52 | 1.77 | 1.49 |
| **77** | 1.75 | 2.37 | 3.33 | 2.00 | 2.42 | 2.67 | 2.63 | 2.71 | 3.04 | 2.60 | 2.48 | 2.53 | 2.30 | 1.58 |
| **76** | 2.09 | 3.01 | 2.80 | 2.54 | 2.02 | 3.04 | 3.05 | 2.41 | 3.04 | 2.99 | 2.85 | 2.45 | 2.77 | 1.64 |
| **75** | 2.14 | 2.32 | 2.61 | 2.52 | 2.28 | 3.25 | 2.97 | 2.85 | 2.80 | 1.73 | 2.96 | 2.39 | 2.97 | 1.32 |
| **74** | 2.12 | 2.37 | 2.27 | 1.96 | 1.86 | 2.65 | 1.47 | 2.50 | 2.73 | 2.00 | 3.11 | 2.08 | 2.89 | 2.68 |
| **73** | 2.00 | 2.23 | 2.61 | 1.91 | 2.24 | 2.83 | N/A | 2.34 | 2.88 | 2.17 | 1.92 | 1.95 | 2.84 | 1.64 |
| **72** | 1.81 | 3.14 | 2.45 | 1.92 | 1.76 | 3.14 | 0.97 | 2.36 | 2.62 | 2.49 | 2.03 | 2.33 | 2.19 | 2.01 |
| **71** | 1.48 | 2.94 | 2.52 | 2.08 | 1.95 | 3.41 | 2.04 | 2.25 | 3.27 | 2.39 | 2.49 | 2.36 | 2.43 | 2.65 |
| **70** | 1.35 | 2.16 | 2.47 | 2.39 | N/A | 2.84 | 2.21 | 2.53 | 2.70 | 2.69 | 2.06 | 2.45 | 2.21 | 2.78 |
| **69** | 2.02 | 1.63 | 2.37 | N/A | 2.35 | 3.05 | 2.20 | 2.81 | 3.05 | 2.22 | 2.09 | 2.28 | 2.45 | 3.07 |
| **68** | 2.22 | 2.46 | 2.35 | 1.69 | 2.69 | 3.19 | 2.55 | 3.11 | 3.08 | 2.69 | 2.30 | 2.39 | 2.75 | 2.08 |
| **67** | 1.67 | 2.31 | 2.86 | 2.26 | 2.61 | 3.10 | 2.62 | 2.10 | 3.04 | 3.08 | 2.63 | 2.75 | 2.19 | 2.51 |
| **66** | 2.00 | 2.81 | 2.61 | 1.88 | 2.38 | 3.29 | 2.93 | 2.46 | 3.27 | 2.79 | 2.74 | 1.92 | 2.72 | 2.70 |
| **65** | 2.52 | 2.90 | 2.19 | 2.17 | 1.90 | 2.92 | 2.76 | 2.68 | 3.02 | 2.07 | 2.14 | 2.32 | 2.28 | 2.07 |
| **64** | 2.21 | 2.48 | 2.62 | 2.18 | 1.83 | 2.83 | 2.62 | 2.84 | 3.34 | 2.24 | 2.54 | 2.75 | 2.66 | 2.35 |
| **63** | 1.87 | 2.37 | 3.08 | 2.96 | 1.78 | 2.95 | 2.50 | 2.69 | 3.42 | 1.93 | 2.33 | 2.83 | 2.75 | 2.81 |
| **62** | 1.91 | 2.31 | 2.98 | 3.59 | 1.73 | 2.53 | 1.41 | 2.59 | 3.15 | 2.30 | 2.32 | 2.68 | 2.90 | 2.38 |
| **61** | 2.31 | 2.19 | 2.50 | 3.27 | N/A | 2.30 | 2.03 | 1.56 | 3.07 | 2.66 | 2.40 | 2.51 | 2.39 | 2.63 |
| **60** | 1.42 | 1.92 | 2.31 | 2.98 | 1.99 | 3.43 | N/A | 2.01 | 3.04 | 2.66 | 2.96 | 2.81 | 2.74 | 1.94 |
| **59** | 2.11 | 2.82 | 2.33 | 2.54 | 2.37 | 3.90 | 2.30 | 2.91 | 3.44 | 3.19 | 2.70 | 2.67 | 2.66 | 2.30 |
| **58** | 2.03 | 2.06 | 2.75 | 2.10 | 2.27 | 3.26 | 2.61 | 2.32 | 3.02 | 2.75 | 2.43 | 2.63 | 3.37 | 2.36 |
| **57** | 2.52 | 1.51 | 2.49 | 1.32 | 2.29 | 3.66 | 2.66 | 2.80 | 2.96 | 2.39 | 2.48 | 2.65 | 2.92 | 2.31 |
| **56** | 2.56 | 2.34 | 2.51 | 1.40 | 2.67 | 3.10 | 2.95 | 2.95 | 2.62 | 2.24 | 2.49 | 2.31 | 2.65 | 2.29 |
| **55** | 2.01 | 2.70 | 2.57 | 1.65 | 2.86 | 3.08 | 1.66 | 2.15 | 2.89 | 2.30 | 2.91 | 2.33 | 2.33 | 2.40 |
| **54** | 1.24 | 2.34 | 2.62 | 1.97 | 2.59 | 3.55 | 2.40 | 2.09 | 3.32 | 2.68 | 3.29 | 2.35 | 2.31 | 2.12 |
| **53** | 2.06 | 1.82 | 2.97 | 1.91 | 2.02 | 3.45 | 2.26 | 2.28 | 3.01 | 2.83 | 2.63 | 2.32 | 2.24 | 1.91 |
| **52** | 3.01 | 2.63 | 3.65 | 0.68 | 1.43 | 3.41 | 1.71 | 2.89 | 2.96 | 2.78 | 2.89 | 2.49 | 2.30 | 2.08 |
| **51** | 2.03 | 2.18 | 3.31 | N/A | 1.83 | 3.39 | 2.52 | 2.05 | 2.97 | 2.68 | 2.42 | 2.91 | 2.44 | 2.17 |
| **50** | 1.95 | 2.54 | 3.17 | 2.35 | 2.11 | 3.13 | 1.54 | 1.96 | 2.63 | 2.68 | 2.40 | 2.62 | 1.81 | 1.50 |
| **49** | 1.52 | 2.51 | 2.54 | 2.54 | 1.03 | 2.51 | 1.60 | 1.29 | 2.92 | 2.39 | 2.58 | 2.46 | 2.53 | 1.66 |
| **48** | 1.47 | 2.05 | 2.45 | 2.75 | 1.67 | 2.72 | 1.09 | 1.46 | 3.41 | 2.36 | 2.63 | 2.23 | 1.99 | 2.60 |
| **47** | 2.33 | 2.68 | 3.20 | 1.98 | 2.01 | 3.54 | 1.77 | 1.73 | 3.62 | 2.26 | 2.11 | 2.22 | 2.00 | 2.76 |
| **46** | 1.98 | 2.72 | 2.11 | 2.49 | 0.91 | 3.48 | 2.28 | 1.92 | 2.74 | 2.49 | 2.01 | 2.19 | 1.86 | 2.07 |
| **45** | 2.25 | 2.71 | 1.73 | 2.57 | 1.29 | 3.42 | 2.48 | 2.00 | 3.02 | 1.97 | 2.14 | 2.84 | 1.85 | 2.68 |
| **44** | 2.16 | 2.91 | 2.55 | 3.16 | 2.36 | 2.42 | 1.72 | 2.46 | 2.71 | 3.18 | 2.32 | 3.30 | 2.13 | 2.31 |
| **43** | 2.79 | 2.60 | 2.64 | 2.86 | 2.16 | 3.52 | 1.97 | 1.36 | 3.21 | 3.53 | 1.77 | 3.13 | 2.10 | 2.49 |
| **42** | 2.80 | 2.83 | 2.57 | 2.38 | 1.92 | 3.52 | 2.80 | 2.22 | 3.43 | 2.32 | 2.07 | 2.72 | 2.29 | 2.34 |
| **41** | 2.05 | 2.69 | 2.50 | 2.90 | 1.85 | 3.43 | 2.51 | 2.13 | 3.15 | 2.44 | 1.76 | 2.56 | 2.37 | 1.83 |
| **40** | 2.39 | 2.67 | 2.54 | 2.75 | 2.11 | 2.79 | 2.59 | 2.06 | 3.01 | 2.19 | N/A | 3.14 | 2.49 | 2.08 |
| **39** | 1.46 | 2.60 | 2.62 | 2.45 | 2.88 | 3.04 | 2.85 | 2.30 | 2.68 | 2.38 | 1.94 | 2.79 | 1.89 | 2.64 |
| **38** | 1.89 | 2.41 | 2.61 | 2.30 | 2.70 | 2.64 | 2.96 | 2.38 | 2.66 | 1.58 | N/A | 3.07 | 2.35 | 2.53 |
| **37** | 1.74 | 1.97 | 2.29 | 2.70 | 2.80 | 3.30 | 3.18 | 2.56 | 2.78 | 1.95 | 1.79 | 3.36 | 2.73 | 2.57 |
| **36** | 1.83 | 1.94 | 2.87 | 3.01 | 2.58 | 3.02 | 2.62 | 2.56 | 3.38 | 2.80 | 2.28 | 3.45 | 2.52 | 2.56 |
| **35** | 1.57 | 2.17 | 3.00 | 2.96 | 2.98 | 3.12 | 3.23 | 2.68 | 3.78 | 2.48 | 2.31 | 3.34 | 2.73 | 2.93 |
| **34** | 2.41 | 1.70 | 3.05 | 1.93 | 2.58 | 3.12 | 2.71 | 2.75 | 3.43 | 2.61 | 1.96 | 2.70 | 2.26 | 2.46 |
| **33** | 2.29 | 2.10 | 2.80 | 1.66 | 2.46 | 3.18 | 3.01 | 2.97 | 3.00 | 2.50 | 2.26 | 2.43 | 0.60 | 2.94 |
| **32** | 1.88 | 1.97 | 2.99 | 2.62 | 2.77 | 2.86 | 2.80 | 2.61 | 3.67 | 2.47 | 2.43 | 2.71 | 2.22 | 2.95 |
| **31** | 2.09 | 1.88 | 3.05 | 2.83 | 2.67 | 3.35 | 2.67 | 2.31 | 3.31 | 2.10 | 2.55 | 3.11 | 1.54 | 2.75 |
| **30** | 2.06 | 1.97 | 2.94 | 2.28 | 2.11 | 3.29 | 2.76 | N/A | 3.20 | 1.84 | 2.40 | 2.83 | 1.03 | 2.25 |
| **29** | 2.27 | 2.50 | 2.24 | 2.52 | 2.30 | 3.75 | 2.05 | 1.94 | 3.28 | 2.67 | 2.91 | 2.97 | 1.80 | 2.72 |
| **28** | 1.82 | 2.72 | 2.64 | 2.53 | 2.25 | 3.57 | 2.08 | 3.13 | 3.22 | 2.97 | 2.93 | 3.01 | 2.37 | 2.68 |
| **27** | 1.79 | 2.54 | 2.76 | 2.73 | 2.37 | 3.48 | 2.95 | 3.37 | 3.45 | 3.27 | 2.39 | 2.98 | 2.41 | 2.39 |
| **26** | 1.71 | 2.01 | 2.75 | 2.57 | 2.16 | 3.48 | 3.53 | 2.94 | 3.38 | 2.86 | 2.26 | 3.30 | 2.91 | 2.75 |
| **25** | 1.79 | 1.88 | 2.72 | 1.89 | 1.94 | 3.32 | 2.21 | 3.32 | 2.79 | 3.09 | 2.82 | 3.10 | 2.77 | 2.56 |
| **24** | 1.61 | 2.08 | 2.66 | 2.59 | 2.17 | 2.83 | 2.06 | 3.63 | 3.20 | 3.39 | 2.88 | 3.13 | 1.74 | 2.63 |
| **23** | 1.64 | 1.62 | 2.99 | 2.47 | 2.16 | 3.19 | N/A | 3.01 | 2.99 | 2.80 | 2.16 | 3.06 | 1.93 | 3.29 |
| **22** | 1.63 | N/A | 2.48 | 2.05 | 1.90 | 3.57 | N/A | 3.03 | 3.11 | 2.58 | 1.82 | 3.05 | 1.30 | 2.90 |
| **21** | 2.04 | 2.63 | 2.44 | 2.06 | 2.38 | 3.04 | N/A | 2.62 | 2.27 | 2.08 | 3.09 | 3.33 | 1.39 | 2.96 |
| **20** | 1.80 | 2.84 | 2.22 | 2.96 | 2.87 | 3.44 | N/A | 2.66 | 2.23 | 1.89 | 3.07 | 3.07 | 1.99 | 3.03 |
| **19** | 2.44 | 2.88 | 2.32 | 3.27 | 2.26 | 3.47 | N/A | 2.63 | 3.43 | 2.84 | 3.28 | 3.13 | 2.21 | 2.99 |
| **18** | 3.09 | 2.52 | 2.16 | 3.42 | 2.28 | 2.98 | N/A | 2.30 | 3.85 | 2.38 | 2.99 | 2.94 | 1.68 | 2.55 |
| **17** | 2.13 | 2.58 | 2.84 | 2.72 | 1.99 | 3.09 | 2.85 | 1.85 | 3.54 | 2.67 | 2.80 | 3.23 | 1.84 | 2.64 |
| **16** | 2.61 | 2.66 | 2.60 | 1.93 | 2.15 | 2.81 | 3.13 | 2.71 | 3.44 | 1.82 | 2.93 | 3.35 | 2.17 | 2.25 |
| **15** | 2.22 | 2.44 | 2.07 | 1.79 | 2.23 | 3.13 | 3.66 | 2.60 | 3.19 | 2.26 | 3.60 | 3.29 | 2.15 | 2.90 |
| **14** | 1.88 | 2.19 | 1.81 | 2.22 | 2.56 | 2.79 | 3.48 | 2.56 | 3.10 | 2.27 | 2.51 | 3.11 | 2.47 | 2.96 |
| **13** | 2.61 | 1.87 | 1.80 | 2.46 | 1.80 | 3.43 | 2.47 | 3.19 | 2.95 | 1.96 | 2.50 | 2.84 | 2.72 | 3.18 |
| **12** | 2.75 | 2.16 | 1.97 | 1.77 | 1.79 | 3.53 | 3.11 | 2.84 | 3.13 | 2.64 | 2.91 | 2.87 | 3.13 | 2.85 |
| **11** | 2.09 | 2.44 | 1.71 | 1.90 | 1.56 | 2.98 | 3.58 | 3.14 | 2.71 | 2.87 | 2.76 | 2.97 | 1.96 | 2.97 |
| **10** | 1.85 | 1.95 | 2.33 | 2.66 | 1.51 | 2.94 | 3.68 | 2.39 | 2.57 | 2.66 | 2.88 | 2.20 | 2.38 | 2.77 |
| **9** | 2.44 | 1.27 | 2.33 | 2.67 | 2.08 | 3.37 | 2.85 | 2.03 | 2.77 | 2.94 | 3.36 | 3.05 | 2.18 | 2.46 |
| **8** | 2.13 | 2.73 | 2.50 | 2.64 | 1.79 | 3.78 | 2.23 | 2.18 | 2.86 | 2.30 | 2.74 | 3.15 | 1.98 | 1.98 |
| **7** | 1.68 | 2.71 | 2.14 | 2.91 | 1.82 | 3.10 | 2.26 | 2.48 | 3.03 | 2.60 | 2.78 | 3.10 | 1.75 | 2.33 |
| **6** | 1.67 | 1.97 | 1.60 | 2.63 | 2.08 | 2.35 | 2.45 | 2.33 | 3.21 | 2.43 | 2.67 | 3.33 | 2.48 | 2.58 |
| **5** | 2.00 | 2.96 | 1.85 | 2.57 | 1.82 | 3.04 | 2.28 | 2.50 | 3.36 | 2.48 | 2.69 | 3.20 | 1.96 | 2.17 |
| **4** | 1.45 | 2.20 | 1.92 | 2.53 | 2.21 | 2.58 | 2.34 | 2.42 | 3.29 | 2.23 | 1.92 | 2.18 | 1.53 | 2.17 |
| **3** | 1.96 | 0.91 | 1.71 | 1.84 | 1.76 | 2.07 | 1.61 | 1.93 | 2.67 | 1.79 | 1.74 | 2.08 | 1.20 | 2.28 |
| **2** | 1.36 | 0.88 | 1.72 | 1.70 | 1.50 | 2.15 | 1.28 | 1.74 | 2.25 | 1.13 | 1.94 | 1.96 | 1.63 | 1.94 |
| **1** | 1.03 | 1.56 | 1.25 | 1.48 | 1.66 | 1.94 | 0.92 | 1.83 | 1.71 | 1.44 | 1.98 | 1.31 | 2.06 | 1.33 |

### Table S12. Tree-level blossom cluster number measured in the apple orchard on 16^th^ April 2024.

| **Col**  **Row** | **1** | **2** | **3** | **4** | **5** | **6** | **7** | **8** | **9** | **10** | **11** | **12** | **13** | **14** |
| --- | --- | --- | --- | --- | --- | --- | --- | --- | --- | --- | --- | --- | --- | --- |
| **80** | 52 | 49 | 29 | 108 | 102 | 56 | 76 | 67 | 5 | 77 | 62 | 93 | 41 | 55 |
| **79** | 48 | 48 | 14 | 75 | 86 | 12 | 60 | 96 | 74 | 70 | 65 | 51 | 100 | 31 |
| **78** | 41 | 108 | 60 | 102 | 62 | 91 | 95 | 99 | 109 | 90 | 73 | 74 | 38 | 45 |
| **77** | 86 | 75 | 71 | 53 | 78 | 116 | 56 | 151 | 152 | 106 | 104 | 110 | 40 | 43 |
| **76** | 98 | 165 | 38 | 125 | 38 | 78 | 130 | 122 | 134 | 195 | 114 | 87 | 66 | 44 |
| **75** | 76 | 53 | 116 | 95 | 88 | 188 | 75 | 118 | 133 | 132 | 151 | 129 | 87 | 34 |
| **74** | 117 | 86 | 57 | 116 | 80 | 95 | 54 | 79 | 98 | 60 | 115 | 128 | 92 | 77 |
| **73** | 108 | 90 | 25 | 59 | 78 | 116 | N/A | 122 | 166 | 78 | 67 | 140 | 142 | 125 |
| **72** | 85 | 97 | 71 | 72 | 34 | 96 | 50 | 80 | 118 | 114 | 100 | 94 | 131 | 84 |
| **71** | 66 | 149 | 64 | 78 | 60 | 98 | 66 | 127 | 178 | 107 | 100 | 144 | 77 | 79 |
| **70** | 96 | 87 | 121 | 125 | N/A | 97 | 62 | 120 | 155 | 126 | 70 | 117 | 75 | 78 |
| **69** | 82 | 84 | 102 | N/A | 53 | 102 | 59 | 79 | 135 | 94 | 103 | 89 | 129 | 124 |
| **68** | 105 | 77 | 47 | 77 | 106 | 95 | 160 | 138 | 113 | 82 | 87 | 91 | 95 | 78 |
| **67** | 71 | 66 | 99 | 128 | 92 | 60 | 75 | 62 | 130 | 108 | 158 | 132 | 69 | 62 |
| **66** | 104 | 120 | 58 | 145 | 139 | 82 | 77 | 74 | 136 | 175 | 106 | 122 | 71 | 100 |
| **65** | 131 | 101 | 69 | 129 | 104 | 86 | 121 | 73 | 156 | 110 | 106 | 92 | 69 | 92 |
| **64** | 118 | 121 | 54 | 99 | 75 | 93 | 99 | 95 | 159 | 108 | 110 | 124 | 114 | 74 |
| **63** | 150 | 126 | 79 | 115 | 105 | 127 | 103 | 73 | 158 | 51 | 85 | 105 | 107 | 126 |
| **62** | 148 | 101 | 76 | 217 | 107 | 121 | 25 | 157 | 146 | 99 | 84 | 106 | 112 | 70 |
| **61** | 192 | 148 | 45 | 222 | N/A | 101 | 54 | 58 | 125 | 142 | 72 | 101 | 93 | 102 |
| **60** | 46 | 121 | 50 | 176 | 61 | 104 | N/A | 80 | 121 | 143 | 77 | 92 | 75 | 68 |
| **59** | 145 | 128 | 16 | 197 | 90 | 157 | 115 | 117 | 105 | 107 | 75 | 86 | 88 | 81 |
| **58** | 121 | 125 | 56 | 92 | 137 | 48 | 117 | 90 | 113 | 75 | 112 | 53 | 126 | 65 |
| **57** | 126 | 79 | 114 | 62 | 120 | 58 | 111 | 123 | 89 | 99 | 71 | 61 | 102 | 45 |
| **56** | 131 | 71 | 70 | 100 | 70 | 37 | 166 | 102 | 91 | 40 | 79 | 26 | 102 | 82 |
| **55** | 91 | 97 | 40 | 91 | 124 | 60 | 57 | 62 | 132 | 99 | 123 | 52 | 78 | 64 |
| **54** | 91 | 121 | 67 | 78 | 125 | 45 | 71 | 58 | 162 | 75 | 71 | 32 | 93 | 84 |
| **53** | 60 | 50 | 72 | 92 | 88 | 52 | 121 | 88 | 151 | 92 | 67 | 68 | 84 | 41 |
| **52** | 177 | 70 | 42 | 41 | 64 | 76 | 42 | 73 | 195 | 86 | 84 | 48 | 105 | 59 |
| **51** | 125 | 67 | 86 | N/A | 91 | 95 | 93 | 77 | 104 | 122 | 108 | 71 | 60 | 77 |
| **50** | 85 | 84 | 62 | 123 | 97 | 141 | 77 | 63 | 115 | 118 | 102 | 95 | 56 | 43 |
| **49** | 103 | 62 | 30 | 120 | 73 | 108 | 99 | 41 | 133 | 66 | 110 | 111 | 109 | 95 |
| **48** | 57 | 88 | 21 | 91 | 56 | 102 | 62 | 56 | 80 | 91 | 67 | 73 | 54 | 55 |
| **47** | 146 | 106 | 57 | 59 | 166 | 91 | 56 | 87 | 145 | 89 | 68 | 45 | 64 | 80 |
| **46** | 90 | 130 | 31 | 83 | 77 | 92 | 104 | 68 | 102 | 84 | 106 | 55 | 101 | 39 |
| **45** | 124 | 96 | 12 | 71 | 41 | 120 | 109 | 65 | 127 | 81 | 84 | 80 | 89 | 65 |
| **44** | 118 | 48 | 38 | 94 | 38 | 127 | 70 | 76 | 82 | 118 | 61 | 99 | 92 | 106 |
| **43** | 163 | 87 | 52 | 151 | 77 | 142 | 70 | 9 | 71 | 89 | 63 | 82 | 76 | 84 |
| **42** | 162 | 81 | 75 | 75 | 53 | 104 | 101 | 81 | 69 | 100 | 101 | 76 | 70 | 68 |
| **41** | 132 | 128 | 69 | 156 | 31 | 74 | 102 | 103 | 131 | 121 | 73 | 66 | 76 | 33 |
| **40** | 109 | 141 | 81 | 154 | 80 | 77 | 72 | 60 | 118 | 106 | N/A | 53 | 87 | 82 |
| **39** | 119 | 105 | 59 | 105 | 81 | 47 | 62 | 84 | 73 | 45 | 122 | 93 | 40 | 74 |
| **38** | 134 | 78 | 72 | 85 | 81 | 77 | 106 | 85 | 100 | 72 | N/A | 112 | 88 | 67 |
| **37** | 86 | 69 | 62 | 113 | 80 | 84 | 136 | 51 | 22 | 46 | 38 | 67 | 84 | 61 |
| **36** | 91 | 64 | 50 | 116 | 101 | 132 | 108 | 94 | 137 | 96 | 109 | 50 | 44 | 62 |
| **35** | 128 | 97 | 56 | 131 | 108 | 104 | 95 | 68 | 144 | 24 | 58 | 61 | 155 | 59 |
| **34** | 133 | 64 | 26 | 65 | 131 | 65 | 68 | 110 | 119 | 75 | 77 | 60 | 48 | 54 |
| **33** | 156 | 126 | 41 | 40 | 59 | 126 | 171 | 68 | 133 | 71 | 76 | 65 | 30 | 93 |
| **32** | 101 | 114 | 51 | 84 | 58 | 124 | 105 | 79 | 119 | 91 | 104 | 46 | 74 | 71 |
| **31** | 103 | 105 | 51 | 117 | 84 | 113 | 121 | 125 | 99 | 73 | 101 | 71 | 59 | 87 |
| **30** | 63 | 60 | 69 | 88 | 82 | 102 | 115 | N/A | 107 | 57 | 78 | 48 | 31 | 37 |
| **29** | 101 | 50 | 55 | 76 | 49 | 149 | 190 | 83 | 129 | 28 | 119 | 75 | 75 | 75 |
| **28** | 127 | 62 | 72 | 68 | 108 | 108 | 95 | 103 | 126 | 117 | 113 | 90 | 81 | 47 |
| **27** | 76 | 66 | 83 | 130 | 106 | 141 | 88 | 106 | 97 | 93 | 125 | 45 | 101 | 59 |
| **26** | 91 | 86 | 103 | 100 | 103 | 231 | 192 | 110 | 114 | 91 | 67 | 31 | 96 | 81 |
| **25** | 65 | 131 | 76 | 76 | 60 | 209 | 52 | 118 | 65 | 67 | 111 | 54 | 87 | 36 |
| **24** | 84 | 91 | 66 | 109 | 132 | 123 | 116 | 133 | 152 | 135 | 156 | 154 | 49 | 92 |
| **23** | 72 | 106 | 81 | 130 | 60 | 109 | N/A | 120 | 104 | 110 | 98 | 94 | 64 | 31 |
| **22** | 86 | N/A | 49 | 91 | 101 | 196 | N/A | 157 | 178 | 107 | 70 | 83 | 49 | 58 |
| **21** | 93 | 87 | 57 | 106 | 76 | 170 | N/A | 36 | 53 | 63 | 166 | 77 | 33 | 77 |
| **20** | 106 | 119 | 70 | 125 | 141 | 172 | N/A | 114 | 12 | 26 | 117 | 38 | 69 | 49 |
| **19** | 92 | 70 | 67 | 99 | 184 | 156 | N/A | 96 | 38 | 108 | 115 | 68 | 86 | 101 |
| **18** | 111 | 77 | 37 | 69 | 138 | 122 | N/A | 147 | 24 | 32 | 67 | 61 | 63 | 67 |
| **17** | 76 | 57 | 66 | 69 | 84 | 108 | 139 | 83 | 93 | 71 | 101 | 59 | 58 | 51 |
| **16** | 70 | 82 | 43 | 82 | 146 | 102 | 120 | 148 | 120 | 72 | 89 | 102 | 41 | 59 |
| **15** | 92 | 64 | 17 | 78 | 148 | 77 | 201 | 66 | 123 | 95 | 93 | 55 | 62 | 55 |
| **14** | 39 | 92 | 55 | 67 | 171 | 83 | 254 | 77 | 156 | 84 | 63 | 51 | 94 | 70 |
| **13** | 69 | 121 | 62 | 89 | 57 | 77 | 133 | 79 | 169 | 87 | 98 | 79 | 73 | 72 |
| **12** | 47 | 81 | 101 | 71 | 123 | 71 | 92 | 84 | 143 | 81 | 66 | 98 | 152 | 65 |
| **11** | 87 | 119 | 56 | 36 | 88 | 89 | 145 | 81 | 160 | 109 | 104 | 75 | 66 | 77 |
| **10** | 95 | 93 | 39 | 93 | 80 | 77 | 180 | 113 | 159 | 91 | 36 | 77 | 87 | 33 |
| **9** | 122 | 32 | 77 | 140 | 103 | 101 | 69 | 77 | 157 | 104 | 106 | 38 | 102 | 43 |
| **8** | 72 | 134 | 42 | 158 | 67 | 230 | 118 | 103 | 163 | 33 | 97 | 76 | 99 | 78 |
| **7** | 104 | 107 | 82 | 92 | 38 | 144 | 60 | 84 | 132 | 30 | 78 | 80 | 92 | 72 |
| **6** | 80 | 95 | 98 | 33 | 60 | 106 | 70 | 93 | 134 | 40 | 49 | 68 | 80 | 83 |
| **5** | 102 | 172 | 129 | 105 | 111 | 153 | 83 | 40 | 141 | 48 | 110 | 75 | 57 | 62 |
| **4** | 57 | 99 | 86 | 62 | 97 | 119 | 69 | 58 | 115 | 41 | 91 | 36 | 67 | 43 |
| **3** | 78 | 57 | 17 | 116 | 80 | 41 | 68 | 75 | 47 | 61 | 60 | 26 | 63 | 120 |
| **2** | 55 | 35 | 19 | 63 | 86 | 44 | 51 | 96 | 11 | 27 | 81 | 48 | 50 | 58 |
| **1** | 55 | 50 | 66 | 41 | 111 | 63 | 65 | 77 | 67 | 55 | 90 | 1 | 115 | 51 |

### Table S13. Tree-level blossom cluster number measured in the apple orchard on 24^th^ April 2024.

| **Col**  **Row** | **1** | **2** | **3** | **4** | **5** | **6** | **7** | **8** | **9** | **10** | **11** | **12** | **13** | **14** |
| --- | --- | --- | --- | --- | --- | --- | --- | --- | --- | --- | --- | --- | --- | --- |
| **80** | 29 | 48 | 2 | 76 | 76 | 6 | 99 | 33 | 1 | 46 | 34 | 56 | 41 | 49 |
| **79** | 25 | 32 | 6 | 69 | 28 | 1 | 86 | 73 | 11 | 57 | 60 | 15 | 49 | 46 |
| **78** | 19 | 61 | 30 | 95 | 35 | 57 | 57 | 68 | 62 | 47 | 70 | 31 | 32 | 44 |
| **77** | 36 | 54 | 20 | 30 | 43 | 89 | 92 | 108 | 58 | 85 | 65 | 91 | 21 | 31 |
| **76** | 47 | 77 | 10 | 44 | 29 | 63 | 71 | 53 | 71 | 137 | 70 | 54 | 47 | 44 |
| **75** | 38 | 28 | 36 | 59 | 92 | 82 | 126 | 85 | 60 | 45 | 58 | 116 | 48 | 31 |
| **74** | 54 | 51 | 22 | 59 | 45 | 90 | 55 | 50 | 93 | 57 | 53 | 82 | 50 | 80 |
| **73** | 44 | 30 | 2 | 50 | 70 | 81 | N/A | 51 | 87 | 62 | 29 | 67 | 65 | 36 |
| **72** | 50 | 49 | 16 | 43 | 56 | 67 | 23 | 79 | 56 | 47 | 41 | 76 | 57 | 41 |
| **71** | 40 | 86 | 13 | 70 | 54 | 50 | 36 | 63 | 67 | 25 | 88 | 82 | 63 | 54 |
| **70** | 49 | 34 | 19 | 49 | N/A | 74 | 65 | 80 | 70 | 71 | 63 | 69 | 45 | 36 |
| **69** | 49 | 41 | 40 | N/A | 49 | 42 | 56 | 69 | 27 | 47 | 60 | 69 | 36 | 120 |
| **68** | 89 | 46 | 22 | 56 | 38 | 46 | 118 | 80 | 28 | 64 | 45 | 55 | 48 | 53 |
| **67** | 44 | 27 | 53 | 52 | 69 | 12 | 45 | 46 | 54 | 122 | 58 | 63 | 19 | 45 |
| **66** | 46 | 42 | 36 | 66 | 53 | 49 | 71 | 49 | 62 | 65 | 55 | 58 | 52 | 33 |
| **65** | 46 | 29 | 16 | 51 | 32 | 44 | 38 | 51 | 81 | 60 | 70 | 52 | 42 | 40 |
| **64** | 82 | 54 | 10 | 28 | 52 | 40 | 56 | 54 | 44 | 70 | 67 | 43 | 66 | 81 |
| **63** | 45 | 35 | 15 | 24 | 24 | 16 | 79 | 44 | 58 | 54 | 51 | 64 | 76 | 58 |
| **62** | 43 | 56 | 17 | 166 | 65 | 32 | 19 | 113 | 60 | 51 | 43 | 54 | 85 | 78 |
| **61** | 77 | 78 | 4 | 95 | N/A | 44 | 53 | 41 | 65 | 35 | 57 | 38 | 62 | 75 |
| **60** | 32 | 26 | 9 | 76 | 67 | 35 | N/A | 55 | 42 | 40 | 53 | 71 | 54 | 32 |
| **59** | 82 | 46 | 1 | 60 | 43 | 6 | 46 | 77 | 51 | 80 | 33 | 54 | 48 | 44 |
| **58** | 44 | 75 | 20 | 45 | 33 | 7 | 39 | 58 | 53 | 78 | 60 | 16 | 131 | 49 |
| **57** | 47 | 29 | 17 | 39 | 52 | 4 | 36 | 75 | 62 | 59 | 33 | 28 | 97 | 54 |
| **56** | 73 | 37 | 7 | 42 | 33 | 3 | 88 | 55 | 48 | 49 | 43 | 11 | 73 | 67 |
| **55** | 63 | 52 | 9 | 45 | 46 | 11 | 46 | 36 | 37 | 56 | 28 | 19 | 66 | 59 |
| **54** | 53 | 36 | 22 | 49 | 75 | 9 | 54 | 29 | 52 | 53 | 35 | 21 | 53 | 56 |
| **53** | 48 | 45 | 20 | 60 | 51 | 18 | 85 | 57 | 31 | 80 | 45 | 34 | 52 | 40 |
| **52** | 153 | 25 | 29 | 9 | 58 | 22 | 26 | 62 | 56 | 49 | 43 | 32 | 54 | 50 |
| **51** | 71 | 38 | 31 | N/A | 50 | 32 | 57 | 40 | 52 | 121 | 50 | 36 | 79 | 84 |
| **50** | 50 | 42 | 19 | 75 | 49 | 73 | 60 | 63 | 41 | 85 | 95 | 48 | 36 | 62 |
| **49** | 63 | 55 | 23 | 25 | 37 | 24 | 56 | 33 | 42 | 47 | 51 | 57 | 86 | 56 |
| **48** | 53 | 49 | 17 | 71 | 61 | 21 | 39 | 47 | 28 | 62 | 58 | 41 | 37 | 67 |
| **47** | 61 | 58 | 15 | 30 | 98 | 28 | 47 | 46 | 46 | 70 | 46 | 40 | 72 | 59 |
| **46** | 40 | 50 | 8 | 60 | 25 | 42 | 70 | 77 | 62 | 84 | 57 | 32 | 57 | 50 |
| **45** | 57 | 45 | 8 | 32 | 43 | 69 | 100 | 43 | 51 | 55 | 71 | 68 | 38 | 39 |
| **44** | 47 | 39 | 16 | 66 | 61 | 68 | 33 | 85 | 28 | 75 | 50 | 34 | 88 | 72 |
| **43** | 58 | 57 | 22 | 77 | 71 | 58 | 60 | 16 | 36 | 115 | 57 | 70 | 54 | 94 |
| **42** | 69 | 34 | 30 | 44 | 37 | 40 | 59 | 63 | 34 | 59 | 90 | 21 | 69 | 47 |
| **41** | 35 | 71 | 24 | 87 | 46 | 34 | 69 | 100 | 42 | 114 | 71 | 22 | 52 | 24 |
| **40** | 64 | 68 | 46 | 97 | 65 | 23 | 74 | 42 | 37 | 114 | N/A | 31 | 43 | 40 |
| **39** | 55 | 39 | 15 | 74 | 62 | 25 | 57 | 91 | 24 | 77 | 75 | 47 | 34 | 43 |
| **38** | 45 | 45 | 50 | 90 | 72 | 33 | 91 | 59 | 36 | 49 | N/A | 42 | 76 | 29 |
| **37** | 39 | 38 | 21 | 56 | 47 | 30 | 153 | 31 | 9 | 57 | 48 | 44 | 55 | 22 |
| **36** | 44 | 47 | 16 | 58 | 45 | 50 | 51 | 49 | 39 | 70 | 78 | 39 | 45 | 51 |
| **35** | 27 | 48 | 23 | 74 | 54 | 86 | 142 | 84 | 80 | 30 | 51 | 33 | 72 | 62 |
| **34** | 50 | 28 | 15 | 39 | 88 | 14 | 63 | 71 | 57 | 59 | 72 | 26 | 96 | 107 |
| **33** | 44 | 65 | 9 | 39 | 37 | 69 | 89 | 52 | 48 | 46 | 43 | 38 | 51 | 83 |
| **32** | 37 | 40 | 22 | 76 | 47 | 39 | 87 | 68 | 43 | 42 | 54 | 19 | 90 | 73 |
| **31** | 38 | 36 | 21 | 55 | 83 | 33 | 59 | 94 | 30 | 125 | 72 | 56 | 48 | 71 |
| **30** | 25 | 36 | 25 | 75 | 92 | 29 | 68 | N/A | 36 | 72 | 41 | 27 | 56 | 51 |
| **29** | 21 | 22 | 8 | 45 | 52 | 58 | 66 | 52 | 29 | 39 | 97 | 11 | 39 | 25 |
| **28** | 13 | 21 | 16 | 47 | 58 | 68 | 48 | 78 | 21 | 62 | 70 | 44 | 39 | 50 |
| **27** | 34 | 14 | 26 | 43 | 76 | 36 | 75 | 117 | 41 | 77 | 63 | 20 | 73 | 36 |
| **26** | 38 | 30 | 34 | 36 | 50 | 66 | 176 | 63 | 25 | 44 | 64 | 17 | 81 | 68 |
| **25** | 16 | 55 | 15 | 50 | 38 | 60 | 53 | 138 | 9 | 49 | 72 | 20 | 86 | 29 |
| **24** | 41 | 40 | 12 | 74 | 62 | 27 | 71 | 131 | 64 | 84 | 84 | 52 | 48 | 51 |
| **23** | 30 | 30 | 15 | 68 | 40 | 26 | N/A | 78 | 39 | 112 | 91 | 39 | 56 | 25 |
| **22** | 21 | N/A | 11 | 38 | 47 | 56 | N/A | 81 | 80 | 90 | 21 | 58 | 43 | 34 |
| **21** | 48 | 58 | 8 | 69 | 11 | 38 | N/A | 35 | 12 | 64 | 104 | 39 | 43 | 37 |
| **20** | 26 | 54 | 18 | 92 | 36 | 74 | N/A | 53 | 3 | 16 | 59 | 16 | 63 | 42 |
| **19** | 19 | 37 | 14 | 45 | 80 | 38 | N/A | 82 | 21 | 72 | 80 | 28 | 97 | 78 |
| **18** | 16 | 15 | 8 | 56 | 101 | 5 | N/A | 45 | 4 | 15 | 41 | 38 | 66 | 70 |
| **17** | 6 | 5 | 3 | 27 | 50 | 6 | 76 | 39 | 13 | 39 | 47 | 18 | 33 | 18 |
| **16** | 17 | 11 | 8 | 83 | 33 | 6 | 71 | 97 | 22 | 58 | 37 | 25 | 47 | 30 |
| **15** | 19 | 16 | 1 | 46 | 57 | 1 | 137 | 30 | 22 | 36 | 73 | 16 | 51 | 51 |
| **14** | 10 | 47 | 3 | 49 | 63 | 4 | 146 | 48 | 40 | 41 | 66 | 17 | 74 | 51 |
| **13** | 18 | 77 | 17 | 67 | 36 | 7 | 64 | 22 | 24 | 43 | 48 | 45 | 44 | 33 |
| **12** | 13 | 30 | 23 | 57 | 68 | 9 | 57 | 34 | 25 | 38 | 41 | 23 | 108 | 33 |
| **11** | 11 | 87 | 3 | 9 | 31 | 11 | 110 | 61 | 31 | 69 | 64 | 44 | 31 | 40 |
| **10** | 29 | 76 | 7 | 42 | 21 | 4 | 152 | 62 | 21 | 38 | 13 | 5 | 48 | 36 |
| **9** | 34 | 49 | 7 | 54 | 15 | 19 | 58 | 42 | 13 | 36 | 36 | 6 | 73 | 34 |
| **8** | 29 | 63 | 2 | 63 | 28 | 45 | 28 | 23 | 47 | 25 | 36 | 8 | 87 | 46 |
| **7** | 42 | 47 | 12 | 42 | 10 | 18 | 41 | 53 | 51 | 12 | 38 | 16 | 47 | 39 |
| **6** | 30 | 38 | 6 | 5 | 23 | 16 | 26 | 46 | 21 | 18 | 58 | 9 | 64 | 46 |
| **5** | 38 | 83 | 6 | 18 | 30 | 22 | 15 | 49 | 43 | 13 | 49 | 9 | 39 | 40 |
| **4** | 11 | 60 | 11 | 10 | 22 | 15 | 30 | 48 | 43 | 14 | 26 | 6 | 35 | 28 |
| **3** | 31 | 8 | 5 | 20 | 19 | 5 | 33 | 73 | 4 | 60 | 48 | 1 | 46 | 56 |
| **2** | 21 | 15 | N/A | 17 | 19 | 1 | 25 | 70 | N/A | 23 | 53 | 7 | 43 | 21 |
| **1** | 11 | 40 | 6 | 9 | 10 | 1 | 29 | 56 | 8 | 70 | 57 | N/A | 60 | 28 |

### Table S14. Tree-level blossom cluster volume (m³) measured in the apple orchard on 16^th^ April 2024.

| **Col**  **Row** | **1** | **2** | **3** | **4** | **5** | **6** | **7** | **8** | **9** | **10** | **11** | **12** | **13** | **14** |
| --- | --- | --- | --- | --- | --- | --- | --- | --- | --- | --- | --- | --- | --- | --- |
| **80** | 0.122 | 0.120 | 0.073 | 0.289 | 0.290 | 0.103 | 0.244 | 0.159 | 0.012 | 0.277 | 0.223 | 0.308 | 0.121 | 0.131 |
| **79** | 0.104 | 0.101 | 0.041 | 0.278 | 0.252 | 0.021 | 0.158 | 0.273 | 0.172 | 0.292 | 0.189 | 0.154 | 0.201 | 0.102 |
| **78** | 0.111 | 0.312 | 0.183 | 0.375 | 0.146 | 0.329 | 0.270 | 0.270 | 0.366 | 0.304 | 0.209 | 0.203 | 0.124 | 0.163 |
| **77** | 0.247 | 0.245 | 0.196 | 0.194 | 0.164 | 0.357 | 0.214 | 0.403 | 0.487 | 0.318 | 0.340 | 0.439 | 0.113 | 0.130 |
| **76** | 0.319 | 0.418 | 0.085 | 0.255 | 0.120 | 0.349 | 0.450 | 0.276 | 0.487 | 0.506 | 0.336 | 0.319 | 0.224 | 0.162 |
| **75** | 0.210 | 0.150 | 0.475 | 0.373 | 0.334 | 0.574 | 0.376 | 0.352 | 0.456 | 0.308 | 0.370 | 0.373 | 0.275 | 0.113 |
| **74** | 0.283 | 0.277 | 0.160 | 0.393 | 0.169 | 0.448 | 0.220 | 0.316 | 0.421 | 0.186 | 0.398 | 0.400 | 0.245 | 0.262 |
| **73** | 0.329 | 0.154 | 0.063 | 0.292 | 0.307 | 0.522 | N/A | 0.340 | 0.509 | 0.268 | 0.205 | 0.383 | 0.336 | 0.201 |
| **72** | 0.409 | 0.219 | 0.176 | 0.223 | 0.173 | 0.467 | 0.103 | 0.218 | 0.384 | 0.263 | 0.215 | 0.318 | 0.247 | 0.196 |
| **71** | 0.175 | 0.437 | 0.187 | 0.316 | 0.211 | 0.570 | 0.168 | 0.298 | 0.522 | 0.225 | 0.357 | 0.410 | 0.246 | 0.205 |
| **70** | 0.360 | 0.219 | 0.404 | 0.272 | N/A | 0.468 | 0.195 | 0.442 | 0.437 | 0.423 | 0.143 | 0.341 | 0.192 | 0.185 |
| **69** | 0.293 | 0.223 | 0.322 | N/A | 0.128 | 0.417 | 0.290 | 0.221 | 0.308 | 0.269 | 0.293 | 0.249 | 0.244 | 0.277 |
| **68** | 0.405 | 0.240 | 0.153 | 0.224 | 0.420 | 0.390 | 0.429 | 0.421 | 0.328 | 0.278 | 0.194 | 0.306 | 0.223 | 0.219 |
| **67** | 0.239 | 0.229 | 0.261 | 0.412 | 0.310 | 0.203 | 0.317 | 0.141 | 0.390 | 0.385 | 0.379 | 0.396 | 0.154 | 0.140 |
| **66** | 0.319 | 0.419 | 0.167 | 0.392 | 0.441 | 0.331 | 0.362 | 0.231 | 0.531 | 0.442 | 0.287 | 0.314 | 0.221 | 0.216 |
| **65** | 0.491 | 0.326 | 0.231 | 0.362 | 0.415 | 0.420 | 0.357 | 0.257 | 0.546 | 0.296 | 0.322 | 0.332 | 0.182 | 0.189 |
| **64** | 0.386 | 0.354 | 0.129 | 0.311 | 0.265 | 0.435 | 0.410 | 0.230 | 0.508 | 0.336 | 0.302 | 0.293 | 0.314 | 0.200 |
| **63** | 0.330 | 0.322 | 0.184 | 0.351 | 0.345 | 0.375 | 0.489 | 0.170 | 0.517 | 0.212 | 0.317 | 0.366 | 0.282 | 0.290 |
| **62** | 0.446 | 0.395 | 0.211 | 0.720 | 0.383 | 0.405 | 0.097 | 0.508 | 0.449 | 0.304 | 0.234 | 0.353 | 0.297 | 0.187 |
| **61** | 0.606 | 0.697 | 0.119 | 0.639 | N/A | 0.417 | 0.257 | 0.248 | 0.473 | 0.309 | 0.259 | 0.269 | 0.239 | 0.253 |
| **60** | 0.080 | 0.291 | 0.120 | 0.702 | 0.139 | 0.518 | N/A | 0.257 | 0.454 | 0.425 | 0.311 | 0.434 | 0.250 | 0.148 |
| **59** | 0.455 | 0.482 | 0.041 | 0.562 | 0.305 | 0.451 | 0.338 | 0.370 | 0.370 | 0.330 | 0.293 | 0.321 | 0.218 | 0.195 |
| **58** | 0.350 | 0.359 | 0.217 | 0.447 | 0.438 | 0.089 | 0.328 | 0.265 | 0.444 | 0.385 | 0.242 | 0.165 | 0.357 | 0.175 |
| **57** | 0.311 | 0.215 | 0.288 | 0.263 | 0.294 | 0.168 | 0.254 | 0.402 | 0.453 | 0.342 | 0.275 | 0.181 | 0.416 | 0.122 |
| **56** | 0.559 | 0.249 | 0.163 | 0.316 | 0.221 | 0.102 | 0.516 | 0.398 | 0.296 | 0.147 | 0.224 | 0.049 | 0.321 | 0.227 |
| **55** | 0.394 | 0.258 | 0.145 | 0.269 | 0.462 | 0.177 | 0.295 | 0.239 | 0.377 | 0.286 | 0.311 | 0.124 | 0.204 | 0.216 |
| **54** | 0.191 | 0.348 | 0.205 | 0.270 | 0.409 | 0.090 | 0.272 | 0.125 | 0.448 | 0.332 | 0.221 | 0.078 | 0.294 | 0.247 |
| **53** | 0.194 | 0.157 | 0.155 | 0.233 | 0.320 | 0.191 | 0.348 | 0.298 | 0.405 | 0.403 | 0.238 | 0.212 | 0.256 | 0.116 |
| **52** | 0.790 | 0.160 | 0.121 | 0.084 | 0.220 | 0.206 | 0.132 | 0.244 | 0.506 | 0.253 | 0.292 | 0.112 | 0.322 | 0.164 |
| **51** | 0.339 | 0.251 | 0.304 | N/A | 0.232 | 0.335 | 0.341 | 0.219 | 0.488 | 0.447 | 0.292 | 0.242 | 0.223 | 0.217 |
| **50** | 0.296 | 0.240 | 0.165 | 0.330 | 0.307 | 0.515 | 0.285 | 0.193 | 0.311 | 0.386 | 0.428 | 0.201 | 0.083 | 0.115 |
| **49** | 0.452 | 0.245 | 0.105 | 0.280 | 0.256 | 0.340 | 0.284 | 0.108 | 0.403 | 0.232 | 0.277 | 0.362 | 0.385 | 0.184 |
| **48** | 0.157 | 0.216 | 0.063 | 0.268 | 0.138 | 0.319 | 0.179 | 0.169 | 0.242 | 0.335 | 0.202 | 0.222 | 0.181 | 0.157 |
| **47** | 0.316 | 0.291 | 0.166 | 0.201 | 0.389 | 0.302 | 0.242 | 0.235 | 0.353 | 0.188 | 0.228 | 0.151 | 0.252 | 0.192 |
| **46** | 0.239 | 0.390 | 0.092 | 0.233 | 0.169 | 0.347 | 0.371 | 0.182 | 0.313 | 0.291 | 0.284 | 0.140 | 0.218 | 0.109 |
| **45** | 0.363 | 0.266 | 0.024 | 0.200 | 0.100 | 0.440 | 0.296 | 0.163 | 0.452 | 0.215 | 0.244 | 0.349 | 0.263 | 0.165 |
| **44** | 0.284 | 0.147 | 0.098 | 0.362 | 0.150 | 0.312 | 0.181 | 0.222 | 0.178 | 0.241 | 0.157 | 0.272 | 0.234 | 0.285 |
| **43** | 0.284 | 0.169 | 0.123 | 0.448 | 0.173 | 0.377 | 0.185 | 0.032 | 0.289 | 0.440 | 0.212 | 0.347 | 0.211 | 0.205 |
| **42** | 0.392 | 0.213 | 0.194 | 0.200 | 0.123 | 0.274 | 0.233 | 0.224 | 0.226 | 0.318 | 0.293 | 0.189 | 0.247 | 0.143 |
| **41** | 0.335 | 0.264 | 0.159 | 0.424 | 0.166 | 0.177 | 0.412 | 0.284 | 0.332 | 0.380 | 0.159 | 0.207 | 0.180 | 0.081 |
| **40** | 0.357 | 0.355 | 0.243 | 0.475 | 0.270 | 0.230 | 0.210 | 0.181 | 0.321 | 0.376 | N/A | 0.165 | 0.207 | 0.176 |
| **39** | 0.253 | 0.223 | 0.129 | 0.411 | 0.191 | 0.139 | 0.186 | 0.397 | 0.270 | 0.152 | 0.282 | 0.251 | 0.103 | 0.161 |
| **38** | 0.367 | 0.164 | 0.190 | 0.268 | 0.269 | 0.225 | 0.331 | 0.291 | 0.275 | 0.235 | N/A | 0.315 | 0.282 | 0.181 |
| **37** | 0.258 | 0.186 | 0.123 | 0.258 | 0.311 | 0.263 | 0.538 | 0.145 | 0.048 | 0.150 | 0.146 | 0.243 | 0.191 | 0.139 |
| **36** | 0.200 | 0.167 | 0.079 | 0.409 | 0.324 | 0.368 | 0.242 | 0.214 | 0.392 | 0.291 | 0.360 | 0.189 | 0.169 | 0.196 |
| **35** | 0.299 | 0.301 | 0.160 | 0.417 | 0.259 | 0.430 | 0.400 | 0.224 | 0.567 | 0.054 | 0.200 | 0.222 | 0.364 | 0.175 |
| **34** | 0.437 | 0.159 | 0.064 | 0.217 | 0.340 | 0.181 | 0.177 | 0.221 | 0.475 | 0.214 | 0.163 | 0.187 | 0.257 | 0.201 |
| **33** | 0.459 | 0.266 | 0.093 | 0.125 | 0.169 | 0.364 | 0.446 | 0.165 | 0.392 | 0.163 | 0.221 | 0.156 | 0.082 | 0.211 |
| **32** | 0.259 | 0.289 | 0.132 | 0.304 | 0.154 | 0.267 | 0.225 | 0.197 | 0.377 | 0.180 | 0.255 | 0.126 | 0.243 | 0.245 |
| **31** | 0.301 | 0.288 | 0.131 | 0.457 | 0.277 | 0.316 | 0.308 | 0.292 | 0.268 | 0.214 | 0.272 | 0.212 | 0.121 | 0.242 |
| **30** | 0.156 | 0.188 | 0.246 | 0.230 | 0.250 | 0.318 | 0.336 | N/A | 0.324 | 0.171 | 0.204 | 0.084 | 0.087 | 0.083 |
| **29** | 0.216 | 0.170 | 0.144 | 0.198 | 0.165 | 0.420 | 0.411 | 0.188 | 0.274 | 0.086 | 0.337 | 0.255 | 0.170 | 0.148 |
| **28** | 0.244 | 0.177 | 0.168 | 0.196 | 0.255 | 0.572 | 0.251 | 0.266 | 0.262 | 0.374 | 0.303 | 0.226 | 0.127 | 0.166 |
| **27** | 0.178 | 0.158 | 0.193 | 0.345 | 0.241 | 0.380 | 0.205 | 0.229 | 0.235 | 0.269 | 0.237 | 0.136 | 0.213 | 0.169 |
| **26** | 0.187 | 0.282 | 0.246 | 0.270 | 0.257 | 0.550 | 0.568 | 0.363 | 0.302 | 0.202 | 0.218 | 0.071 | 0.256 | 0.244 |
| **25** | 0.148 | 0.294 | 0.197 | 0.172 | 0.173 | 0.508 | 0.107 | 0.453 | 0.123 | 0.184 | 0.279 | 0.153 | 0.285 | 0.092 |
| **24** | 0.168 | 0.311 | 0.142 | 0.478 | 0.406 | 0.387 | 0.263 | 0.454 | 0.379 | 0.408 | 0.331 | 0.326 | 0.161 | 0.207 |
| **23** | 0.170 | 0.249 | 0.199 | 0.356 | 0.159 | 0.390 | N/A | 0.291 | 0.309 | 0.292 | 0.256 | 0.276 | 0.202 | 0.097 |
| **22** | 0.228 | N/A | 0.095 | 0.293 | 0.293 | 0.572 | N/A | 0.420 | 0.484 | 0.293 | 0.170 | 0.246 | 0.105 | 0.146 |
| **21** | 0.337 | 0.264 | 0.113 | 0.324 | 0.228 | 0.410 | N/A | 0.123 | 0.091 | 0.118 | 0.457 | 0.222 | 0.097 | 0.136 |
| **20** | 0.222 | 0.278 | 0.240 | 0.413 | 0.351 | 0.663 | N/A | 0.187 | 0.020 | 0.043 | 0.281 | 0.099 | 0.204 | 0.128 |
| **19** | 0.240 | 0.327 | 0.242 | 0.258 | 0.488 | 0.492 | N/A | 0.242 | 0.083 | 0.324 | 0.336 | 0.210 | 0.223 | 0.217 |
| **18** | 0.259 | 0.152 | 0.108 | 0.169 | 0.445 | 0.268 | N/A | 0.292 | 0.047 | 0.078 | 0.168 | 0.157 | 0.156 | 0.124 |
| **17** | 0.166 | 0.110 | 0.147 | 0.198 | 0.228 | 0.191 | 0.353 | 0.174 | 0.266 | 0.179 | 0.272 | 0.145 | 0.153 | 0.138 |
| **16** | 0.219 | 0.240 | 0.099 | 0.359 | 0.269 | 0.267 | 0.351 | 0.300 | 0.396 | 0.173 | 0.209 | 0.229 | 0.099 | 0.140 |
| **15** | 0.200 | 0.161 | 0.042 | 0.310 | 0.310 | 0.195 | 0.590 | 0.124 | 0.370 | 0.178 | 0.300 | 0.155 | 0.227 | 0.156 |
| **14** | 0.120 | 0.282 | 0.126 | 0.182 | 0.499 | 0.205 | 0.651 | 0.167 | 0.465 | 0.220 | 0.183 | 0.205 | 0.236 | 0.188 |
| **13** | 0.175 | 0.281 | 0.161 | 0.272 | 0.148 | 0.183 | 0.340 | 0.169 | 0.422 | 0.272 | 0.280 | 0.312 | 0.242 | 0.179 |
| **12** | 0.104 | 0.307 | 0.255 | 0.206 | 0.390 | 0.212 | 0.256 | 0.177 | 0.487 | 0.229 | 0.241 | 0.276 | 0.365 | 0.132 |
| **11** | 0.179 | 0.351 | 0.115 | 0.082 | 0.246 | 0.248 | 0.525 | 0.231 | 0.380 | 0.450 | 0.336 | 0.326 | 0.188 | 0.153 |
| **10** | 0.191 | 0.279 | 0.126 | 0.305 | 0.202 | 0.205 | 0.596 | 0.311 | 0.353 | 0.263 | 0.083 | 0.184 | 0.283 | 0.085 |
| **9** | 0.298 | 0.080 | 0.185 | 0.381 | 0.271 | 0.272 | 0.190 | 0.135 | 0.301 | 0.266 | 0.202 | 0.108 | 0.293 | 0.073 |
| **8** | 0.233 | 0.374 | 0.096 | 0.372 | 0.218 | 0.600 | 0.276 | 0.240 | 0.505 | 0.121 | 0.216 | 0.180 | 0.306 | 0.143 |
| **7** | 0.249 | 0.294 | 0.265 | 0.250 | 0.075 | 0.454 | 0.114 | 0.217 | 0.474 | 0.077 | 0.216 | 0.187 | 0.220 | 0.166 |
| **6** | 0.290 | 0.232 | 0.217 | 0.077 | 0.189 | 0.268 | 0.139 | 0.264 | 0.340 | 0.103 | 0.139 | 0.144 | 0.220 | 0.181 |
| **5** | 0.240 | 0.463 | 0.366 | 0.168 | 0.271 | 0.495 | 0.180 | 0.072 | 0.582 | 0.140 | 0.245 | 0.154 | 0.140 | 0.152 |
| **4** | 0.151 | 0.276 | 0.239 | 0.172 | 0.210 | 0.410 | 0.201 | 0.133 | 0.437 | 0.125 | 0.222 | 0.075 | 0.143 | 0.112 |
| **3** | 0.183 | 0.165 | 0.036 | 0.250 | 0.247 | 0.125 | 0.139 | 0.264 | 0.122 | 0.174 | 0.225 | 0.058 | 0.175 | 0.241 |
| **2** | 0.149 | 0.129 | 0.040 | 0.222 | 0.203 | 0.097 | 0.135 | 0.323 | 0.030 | 0.074 | 0.232 | 0.080 | 0.113 | 0.129 |
| **1** | 0.151 | 0.149 | 0.136 | 0.121 | 0.281 | 0.161 | 0.189 | 0.266 | 0.166 | 0.276 | 0.242 | 0.001 | 0.246 | 0.133 |

### Table S15. Tree-level blossom cluster volume (m³) measured in the apple orchard on 24^th^ April 2024.

| **Col**  **Row** | **1** | **2** | **3** | **4** | **5** | **6** | **7** | **8** | **9** | **10** | **11** | **12** | **13** | **14** |
| --- | --- | --- | --- | --- | --- | --- | --- | --- | --- | --- | --- | --- | --- | --- |
| **80** | 0.073 | 0.072 | 0.005 | 0.110 | 0.147 | 0.009 | 0.184 | 0.048 | 0.001 | 0.159 | 0.072 | 0.140 | 0.096 | 0.085 |
| **79** | 0.065 | 0.080 | 0.003 | 0.192 | 0.053 | 0.001 | 0.138 | 0.146 | 0.021 | 0.110 | 0.131 | 0.031 | 0.102 | 0.121 |
| **78** | 0.059 | 0.143 | 0.051 | 0.273 | 0.073 | 0.154 | 0.110 | 0.115 | 0.139 | 0.123 | 0.145 | 0.099 | 0.044 | 0.101 |
| **77** | 0.093 | 0.143 | 0.044 | 0.069 | 0.091 | 0.163 | 0.157 | 0.296 | 0.161 | 0.136 | 0.188 | 0.219 | 0.035 | 0.094 |
| **76** | 0.085 | 0.236 | 0.021 | 0.087 | 0.073 | 0.166 | 0.256 | 0.182 | 0.198 | 0.268 | 0.221 | 0.184 | 0.123 | 0.133 |
| **75** | 0.081 | 0.077 | 0.155 | 0.168 | 0.185 | 0.278 | 0.264 | 0.223 | 0.199 | 0.128 | 0.177 | 0.237 | 0.101 | 0.077 |
| **74** | 0.154 | 0.148 | 0.054 | 0.137 | 0.093 | 0.206 | 0.151 | 0.133 | 0.197 | 0.126 | 0.194 | 0.196 | 0.187 | 0.238 |
| **73** | 0.151 | 0.063 | 0.005 | 0.111 | 0.198 | 0.229 | N/A | 0.281 | 0.206 | 0.165 | 0.079 | 0.207 | 0.242 | 0.110 |
| **72** | 0.150 | 0.143 | 0.036 | 0.119 | 0.162 | 0.185 | 0.078 | 0.203 | 0.189 | 0.135 | 0.108 | 0.202 | 0.132 | 0.124 |
| **71** | 0.072 | 0.235 | 0.022 | 0.116 | 0.172 | 0.172 | 0.106 | 0.210 | 0.182 | 0.078 | 0.230 | 0.296 | 0.193 | 0.155 |
| **70** | 0.179 | 0.090 | 0.065 | 0.135 | N/A | 0.241 | 0.174 | 0.331 | 0.193 | 0.163 | 0.170 | 0.235 | 0.161 | 0.116 |
| **69** | 0.162 | 0.108 | 0.065 | N/A | 0.118 | 0.152 | 0.214 | 0.170 | 0.071 | 0.153 | 0.187 | 0.179 | 0.108 | 0.349 |
| **68** | 0.238 | 0.117 | 0.053 | 0.125 | 0.128 | 0.117 | 0.248 | 0.289 | 0.061 | 0.182 | 0.143 | 0.160 | 0.092 | 0.216 |
| **67** | 0.104 | 0.103 | 0.117 | 0.199 | 0.190 | 0.031 | 0.157 | 0.112 | 0.146 | 0.343 | 0.137 | 0.227 | 0.062 | 0.111 |
| **66** | 0.142 | 0.111 | 0.057 | 0.162 | 0.138 | 0.130 | 0.214 | 0.124 | 0.191 | 0.209 | 0.162 | 0.164 | 0.144 | 0.129 |
| **65** | 0.176 | 0.074 | 0.057 | 0.174 | 0.086 | 0.161 | 0.128 | 0.170 | 0.189 | 0.171 | 0.218 | 0.132 | 0.085 | 0.174 |
| **64** | 0.205 | 0.151 | 0.013 | 0.102 | 0.128 | 0.082 | 0.202 | 0.130 | 0.159 | 0.158 | 0.153 | 0.145 | 0.212 | 0.260 |
| **63** | 0.149 | 0.092 | 0.018 | 0.069 | 0.075 | 0.046 | 0.347 | 0.126 | 0.178 | 0.153 | 0.135 | 0.185 | 0.164 | 0.202 |
| **62** | 0.121 | 0.132 | 0.031 | 0.421 | 0.135 | 0.089 | 0.062 | 0.243 | 0.174 | 0.203 | 0.157 | 0.172 | 0.265 | 0.180 |
| **61** | 0.292 | 0.192 | 0.004 | 0.238 | N/A | 0.093 | 0.219 | 0.165 | 0.207 | 0.115 | 0.148 | 0.123 | 0.164 | 0.249 |
| **60** | 0.063 | 0.067 | 0.022 | 0.223 | 0.201 | 0.106 | N/A | 0.152 | 0.135 | 0.154 | 0.147 | 0.228 | 0.199 | 0.101 |
| **59** | 0.203 | 0.163 | 0.001 | 0.141 | 0.147 | 0.016 | 0.118 | 0.208 | 0.136 | 0.214 | 0.107 | 0.126 | 0.135 | 0.136 |
| **58** | 0.165 | 0.149 | 0.044 | 0.129 | 0.087 | 0.012 | 0.088 | 0.136 | 0.151 | 0.232 | 0.140 | 0.038 | 0.357 | 0.139 |
| **57** | 0.115 | 0.071 | 0.037 | 0.142 | 0.115 | 0.006 | 0.093 | 0.206 | 0.172 | 0.142 | 0.100 | 0.065 | 0.310 | 0.151 |
| **56** | 0.273 | 0.093 | 0.019 | 0.133 | 0.079 | 0.011 | 0.344 | 0.221 | 0.112 | 0.119 | 0.118 | 0.036 | 0.285 | 0.193 |
| **55** | 0.235 | 0.134 | 0.020 | 0.129 | 0.152 | 0.036 | 0.176 | 0.083 | 0.087 | 0.176 | 0.079 | 0.055 | 0.200 | 0.177 |
| **54** | 0.106 | 0.093 | 0.060 | 0.167 | 0.241 | 0.025 | 0.158 | 0.065 | 0.143 | 0.211 | 0.098 | 0.037 | 0.182 | 0.170 |
| **53** | 0.188 | 0.075 | 0.045 | 0.160 | 0.200 | 0.047 | 0.285 | 0.119 | 0.088 | 0.277 | 0.163 | 0.133 | 0.185 | 0.155 |
| **52** | 0.402 | 0.084 | 0.047 | 0.029 | 0.147 | 0.063 | 0.065 | 0.183 | 0.188 | 0.151 | 0.170 | 0.090 | 0.168 | 0.129 |
| **51** | 0.179 | 0.084 | 0.089 | N/A | 0.164 | 0.090 | 0.211 | 0.179 | 0.179 | 0.301 | 0.205 | 0.086 | 0.212 | 0.243 |
| **50** | 0.181 | 0.129 | 0.042 | 0.177 | 0.235 | 0.195 | 0.179 | 0.174 | 0.090 | 0.278 | 0.319 | 0.135 | 0.090 | 0.178 |
| **49** | 0.186 | 0.141 | 0.042 | 0.059 | 0.124 | 0.062 | 0.199 | 0.073 | 0.095 | 0.153 | 0.158 | 0.215 | 0.262 | 0.210 |
| **48** | 0.101 | 0.122 | 0.051 | 0.161 | 0.166 | 0.073 | 0.150 | 0.163 | 0.070 | 0.228 | 0.265 | 0.094 | 0.108 | 0.183 |
| **47** | 0.154 | 0.165 | 0.033 | 0.093 | 0.225 | 0.061 | 0.132 | 0.156 | 0.136 | 0.228 | 0.171 | 0.083 | 0.207 | 0.227 |
| **46** | 0.114 | 0.172 | 0.017 | 0.161 | 0.088 | 0.102 | 0.206 | 0.218 | 0.154 | 0.293 | 0.169 | 0.076 | 0.213 | 0.116 |
| **45** | 0.128 | 0.096 | 0.015 | 0.081 | 0.093 | 0.211 | 0.261 | 0.121 | 0.191 | 0.115 | 0.196 | 0.235 | 0.105 | 0.123 |
| **44** | 0.158 | 0.074 | 0.027 | 0.212 | 0.137 | 0.155 | 0.102 | 0.170 | 0.073 | 0.227 | 0.151 | 0.132 | 0.214 | 0.263 |
| **43** | 0.106 | 0.116 | 0.044 | 0.228 | 0.161 | 0.155 | 0.139 | 0.029 | 0.134 | 0.362 | 0.173 | 0.215 | 0.149 | 0.268 |
| **42** | 0.127 | 0.107 | 0.089 | 0.097 | 0.155 | 0.121 | 0.177 | 0.190 | 0.079 | 0.206 | 0.206 | 0.058 | 0.161 | 0.152 |
| **41** | 0.118 | 0.144 | 0.058 | 0.244 | 0.121 | 0.072 | 0.277 | 0.247 | 0.117 | 0.303 | 0.198 | 0.066 | 0.111 | 0.078 |
| **40** | 0.111 | 0.154 | 0.078 | 0.288 | 0.183 | 0.066 | 0.184 | 0.147 | 0.076 | 0.269 | N/A | 0.055 | 0.139 | 0.124 |
| **39** | 0.090 | 0.099 | 0.034 | 0.167 | 0.184 | 0.059 | 0.171 | 0.258 | 0.090 | 0.170 | 0.233 | 0.131 | 0.096 | 0.144 |
| **38** | 0.077 | 0.078 | 0.085 | 0.194 | 0.167 | 0.072 | 0.251 | 0.188 | 0.097 | 0.172 | N/A | 0.162 | 0.252 | 0.071 |
| **37** | 0.093 | 0.075 | 0.056 | 0.126 | 0.165 | 0.092 | 0.381 | 0.123 | 0.015 | 0.147 | 0.146 | 0.171 | 0.141 | 0.068 |
| **36** | 0.068 | 0.077 | 0.028 | 0.227 | 0.128 | 0.175 | 0.188 | 0.209 | 0.140 | 0.201 | 0.280 | 0.110 | 0.134 | 0.155 |
| **35** | 0.079 | 0.126 | 0.055 | 0.201 | 0.157 | 0.170 | 0.369 | 0.198 | 0.291 | 0.061 | 0.181 | 0.121 | 0.229 | 0.232 |
| **34** | 0.143 | 0.055 | 0.030 | 0.075 | 0.175 | 0.033 | 0.190 | 0.152 | 0.215 | 0.216 | 0.214 | 0.105 | 0.325 | 0.276 |
| **33** | 0.091 | 0.138 | 0.017 | 0.099 | 0.124 | 0.192 | 0.304 | 0.122 | 0.136 | 0.157 | 0.136 | 0.083 | 0.090 | 0.237 |
| **32** | 0.095 | 0.071 | 0.031 | 0.216 | 0.103 | 0.101 | 0.220 | 0.185 | 0.150 | 0.193 | 0.133 | 0.046 | 0.244 | 0.256 |
| **31** | 0.070 | 0.097 | 0.041 | 0.203 | 0.188 | 0.086 | 0.168 | 0.261 | 0.097 | 0.248 | 0.241 | 0.116 | 0.119 | 0.264 |
| **30** | 0.058 | 0.061 | 0.039 | 0.124 | 0.191 | 0.076 | 0.257 | N/A | 0.129 | 0.155 | 0.107 | 0.058 | 0.143 | 0.110 |
| **29** | 0.056 | 0.042 | 0.012 | 0.119 | 0.106 | 0.182 | 0.244 | 0.159 | 0.104 | 0.158 | 0.265 | 0.018 | 0.103 | 0.076 |
| **28** | 0.034 | 0.046 | 0.042 | 0.081 | 0.208 | 0.245 | 0.142 | 0.258 | 0.058 | 0.232 | 0.254 | 0.126 | 0.105 | 0.144 |
| **27** | 0.062 | 0.026 | 0.055 | 0.116 | 0.221 | 0.113 | 0.210 | 0.238 | 0.097 | 0.204 | 0.307 | 0.043 | 0.223 | 0.109 |
| **26** | 0.050 | 0.074 | 0.091 | 0.095 | 0.191 | 0.177 | 0.655 | 0.260 | 0.066 | 0.142 | 0.218 | 0.040 | 0.368 | 0.217 |
| **25** | 0.025 | 0.137 | 0.032 | 0.100 | 0.128 | 0.131 | 0.168 | 0.464 | 0.026 | 0.199 | 0.313 | 0.054 | 0.384 | 0.082 |
| **24** | 0.055 | 0.092 | 0.019 | 0.192 | 0.192 | 0.072 | 0.210 | 0.462 | 0.175 | 0.326 | 0.329 | 0.139 | 0.169 | 0.099 |
| **23** | 0.060 | 0.073 | 0.028 | 0.201 | 0.101 | 0.069 | N/A | 0.213 | 0.094 | 0.326 | 0.373 | 0.110 | 0.164 | 0.068 |
| **22** | 0.056 | N/A | 0.024 | 0.108 | 0.098 | 0.129 | N/A | 0.277 | 0.208 | 0.262 | 0.043 | 0.134 | 0.101 | 0.098 |
| **21** | 0.109 | 0.133 | 0.025 | 0.199 | 0.028 | 0.097 | N/A | 0.117 | 0.026 | 0.187 | 0.325 | 0.117 | 0.143 | 0.096 |
| **20** | 0.048 | 0.123 | 0.033 | 0.255 | 0.104 | 0.217 | N/A | 0.177 | 0.004 | 0.040 | 0.203 | 0.029 | 0.183 | 0.141 |
| **19** | 0.045 | 0.077 | 0.030 | 0.139 | 0.248 | 0.098 | N/A | 0.204 | 0.048 | 0.228 | 0.219 | 0.083 | 0.232 | 0.256 |
| **18** | 0.035 | 0.022 | 0.015 | 0.100 | 0.226 | 0.007 | N/A | 0.154 | 0.006 | 0.045 | 0.083 | 0.092 | 0.203 | 0.214 |
| **17** | 0.008 | 0.006 | 0.006 | 0.055 | 0.118 | 0.009 | 0.170 | 0.098 | 0.035 | 0.192 | 0.145 | 0.041 | 0.063 | 0.052 |
| **16** | 0.047 | 0.016 | 0.015 | 0.229 | 0.093 | 0.017 | 0.190 | 0.265 | 0.055 | 0.156 | 0.074 | 0.049 | 0.134 | 0.103 |
| **15** | 0.046 | 0.041 | 0.001 | 0.168 | 0.161 | 0.003 | 0.409 | 0.087 | 0.051 | 0.094 | 0.222 | 0.046 | 0.144 | 0.143 |
| **14** | 0.027 | 0.108 | 0.010 | 0.095 | 0.250 | 0.007 | 0.480 | 0.101 | 0.157 | 0.117 | 0.159 | 0.048 | 0.186 | 0.106 |
| **13** | 0.032 | 0.137 | 0.036 | 0.167 | 0.097 | 0.015 | 0.209 | 0.057 | 0.063 | 0.155 | 0.101 | 0.136 | 0.152 | 0.085 |
| **12** | 0.025 | 0.068 | 0.059 | 0.151 | 0.221 | 0.021 | 0.158 | 0.089 | 0.082 | 0.078 | 0.142 | 0.052 | 0.385 | 0.100 |
| **11** | 0.023 | 0.276 | 0.007 | 0.015 | 0.102 | 0.031 | 0.289 | 0.146 | 0.058 | 0.215 | 0.171 | 0.114 | 0.129 | 0.109 |
| **10** | 0.042 | 0.190 | 0.012 | 0.073 | 0.065 | 0.010 | 0.420 | 0.219 | 0.055 | 0.104 | 0.033 | 0.006 | 0.149 | 0.091 |
| **9** | 0.081 | 0.089 | 0.016 | 0.122 | 0.027 | 0.033 | 0.101 | 0.125 | 0.042 | 0.101 | 0.072 | 0.025 | 0.195 | 0.091 |
| **8** | 0.061 | 0.191 | 0.006 | 0.176 | 0.055 | 0.094 | 0.048 | 0.059 | 0.119 | 0.043 | 0.083 | 0.015 | 0.193 | 0.113 |
| **7** | 0.086 | 0.117 | 0.028 | 0.079 | 0.015 | 0.049 | 0.102 | 0.137 | 0.140 | 0.030 | 0.070 | 0.040 | 0.133 | 0.101 |
| **6** | 0.072 | 0.106 | 0.015 | 0.009 | 0.053 | 0.030 | 0.056 | 0.157 | 0.063 | 0.036 | 0.125 | 0.021 | 0.145 | 0.122 |
| **5** | 0.087 | 0.232 | 0.009 | 0.035 | 0.054 | 0.053 | 0.028 | 0.191 | 0.097 | 0.032 | 0.113 | 0.019 | 0.093 | 0.086 |
| **4** | 0.027 | 0.101 | 0.021 | 0.021 | 0.040 | 0.034 | 0.072 | 0.141 | 0.093 | 0.027 | 0.053 | 0.011 | 0.072 | 0.051 |
| **3** | 0.082 | 0.022 | 0.005 | 0.044 | 0.037 | 0.009 | 0.072 | 0.175 | 0.011 | 0.117 | 0.138 | 0.001 | 0.107 | 0.174 |
| **2** | 0.044 | 0.034 | N/A | 0.040 | 0.041 | 0.002 | 0.053 | 0.191 | N/A | 0.050 | 0.121 | 0.009 | 0.128 | 0.064 |
| **1** | 0.016 | 0.075 | 0.007 | 0.014 | 0.031 | 0.001 | 0.069 | 0.143 | 0.017 | 0.169 | 0.138 | N/A | 0.124 | 0.064 |

### Table S16. Tree-level apple number in the orchard on 27^th^ August 2024.

| **Col**  **Row** | **1** | **2** | **3** | **4** | **5** | **6** | **7** | **8** | **9** | **10** | **11** | **12** | **13** | **14** |
| --- | --- | --- | --- | --- | --- | --- | --- | --- | --- | --- | --- | --- | --- | --- |
| **80** | 51 | 14 | 15 | 68 | 91 | 25 | 82 | 39 | 4 | 101 | 83 | 59 | 33 | 18 |
| **79** | 21 | 18 | 6 | 79 | 50 | 9 | 54 | 94 | 21 | 66 | 92 | 40 | 47 | 38 |
| **78** | 33 | 50 | 51 | 99 | 56 | 45 | 53 | 60 | 62 | 70 | 99 | 70 | 26 | 17 |
| **77** | 29 | 45 | 70 | 64 | 66 | 63 | 98 | 132 | 89 | 83 | 105 | 50 | 66 | 33 |
| **76** | 43 | 104 | 33 | 93 | 41 | 52 | 164 | 117 | 56 | 175 | 137 | 52 | 71 | 33 |
| **75** | 42 | 88 | 36 | 122 | 82 | 76 | 91 | 139 | 92 | 84 | 133 | 56 | 83 | 28 |
| **74** | 61 | 51 | 35 | 87 | 55 | 72 | 50 | 120 | 78 | 83 | 146 | 51 | 106 | 90 |
| **73** | 103 | 50 | 32 | 57 | 95 | 65 | N/A | 87 | 57 | 103 | 77 | 105 | 127 | 80 |
| **72** | 59 | 71 | 38 | 72 | 63 | 82 | 54 | 126 | 60 | 111 | 108 | 60 | 143 | 48 |
| **71** | 20 | 105 | 52 | 75 | 99 | 68 | 74 | 110 | 82 | 121 | 121 | 84 | 125 | 76 |
| **70** | 66 | 51 | 40 | 112 | N/A | 50 | 108 | 123 | 59 | 159 | 112 | 85 | 142 | 62 |
| **69** | 46 | 43 | 57 | N/A | 143 | 65 | 165 | 102 | 94 | 144 | 149 | 98 | 118 | 98 |
| **68** | 72 | 35 | 51 | 50 | 109 | 96 | 152 | 174 | 96 | 154 | 150 | 57 | 91 | 80 |
| **67** | 40 | 61 | 41 | 58 | 122 | 65 | 136 | 102 | 82 | 163 | 124 | 92 | 78 | 49 |
| **66** | 93 | 73 | 51 | 73 | 114 | 68 | 123 | 51 | 105 | 188 | 198 | 80 | 80 | 59 |
| **65** | 71 | 65 | 22 | 82 | 88 | 63 | 152 | 95 | 80 | 99 | 129 | 57 | 64 | 75 |
| **64** | 68 | 73 | 27 | 55 | 80 | 76 | 142 | 83 | 83 | 164 | 155 | 67 | 90 | 61 |
| **63** | 44 | 40 | 46 | 48 | 101 | 65 | 137 | 95 | 83 | 108 | 145 | 75 | 122 | 89 |
| **62** | 75 | 71 | 48 | 84 | 72 | 50 | 28 | 154 | 74 | 123 | 101 | 61 | 147 | 48 |
| **61** | 76 | 71 | 21 | 66 | N/A | 76 | 93 | 138 | 73 | 122 | 85 | 34 | 78 | 127 |
| **60** | 52 | 37 | 29 | 92 | 64 | 68 | N/A | 75 | 78 | 150 | 151 | 51 | 92 | 74 |
| **59** | 61 | 71 | N/A | 126 | 112 | 56 | 72 | 123 | 91 | 95 | 111 | 47 | 111 | 80 |
| **58** | 84 | 53 | 53 | 59 | 117 | 26 | 76 | 81 | 69 | 144 | 99 | 59 | 120 | 62 |
| **57** | 42 | 44 | 46 | 32 | 90 | 35 | 82 | 98 | 64 | 124 | 149 | 54 | 115 | 116 |
| **56** | 56 | 67 | 36 | 64 | 97 | 40 | 148 | 117 | 46 | 77 | 81 | 54 | 116 | 68 |
| **55** | 72 | 57 | 18 | 41 | 115 | 35 | 89 | 66 | 82 | 88 | 137 | 31 | 85 | 112 |
| **54** | 49 | 61 | 52 | 56 | 101 | 46 | 74 | 73 | 71 | 115 | 104 | 43 | 132 | 76 |
| **53** | 25 | 70 | 46 | 44 | 73 | 33 | 86 | 104 | 48 | 164 | 83 | 50 | 140 | 97 |
| **52** | 66 | 77 | 33 | 60 | 46 | 48 | 44 | 53 | 76 | 154 | 124 | 70 | 86 | 76 |
| **51** | 28 | 63 | 36 | N/A | 34 | 47 | 73 | 75 | 52 | 151 | 148 | 55 | 112 | 83 |
| **50** | 81 | 31 | 40 | 66 | 80 | 89 | 43 | 69 | 54 | 117 | 126 | 77 | 72 | 79 |
| **49** | 59 | 55 | 17 | 71 | 51 | 46 | 66 | 45 | 62 | 116 | 96 | 50 | 73 | 45 |
| **48** | 20 | 39 | 9 | 66 | 46 | 50 | 52 | 77 | 63 | 107 | 121 | 51 | 55 | 50 |
| **47** | 46 | 64 | 21 | 36 | 125 | 44 | 53 | 92 | 58 | 88 | 86 | 67 | 85 | 72 |
| **46** | 63 | 81 | 18 | 91 | 60 | 60 | 86 | 82 | 50 | 121 | 135 | 44 | 56 | 100 |
| **45** | 59 | 59 | 7 | 59 | 10 | 65 | 124 | 67 | 74 | 109 | 95 | 68 | 57 | 75 |
| **44** | 58 | 64 | 38 | 99 | 57 | 40 | 60 | 152 | 80 | 94 | 144 | 49 | 103 | 74 |
| **43** | 43 | 65 | 37 | 104 | 112 | 96 | 78 | 18 | 86 | 159 | 118 | 57 | 72 | 84 |
| **42** | 52 | 49 | 50 | 70 | 71 | 88 | 102 | 62 | 64 | 124 | 105 | 66 | 85 | 60 |
| **41** | 58 | 82 | 57 | 59 | 113 | 69 | 143 | 183 | 59 | 171 | 113 | 56 | 80 | 69 |
| **40** | 44 | 66 | 43 | 131 | 121 | 54 | 99 | 81 | 93 | 119 | N/A | 38 | 82 | 51 |
| **39** | 42 | 67 | 43 | 151 | 111 | 33 | 105 | 156 | 83 | 151 | 80 | 57 | 56 | 89 |
| **38** | 56 | 42 | 63 | 111 | 84 | 70 | 126 | 115 | 56 | 72 | N/A | 51 | 102 | 66 |
| **37** | 97 | 75 | 37 | 86 | 82 | 43 | 117 | 142 | 5 | 54 | 39 | 86 | 60 | 67 |
| **36** | 50 | 42 | 30 | 77 | 92 | 60 | 151 | 81 | 89 | 129 | 139 | 43 | 90 | 67 |
| **35** | 42 | 96 | 71 | 99 | 108 | 86 | 163 | 118 | 96 | 83 | 120 | 60 | 88 | 54 |
| **34** | 62 | 25 | 40 | 50 | 102 | 40 | 141 | 72 | 89 | 44 | 180 | 61 | 134 | 75 |
| **33** | 67 | 44 | 37 | 42 | 90 | 64 | 167 | 99 | 88 | 150 | 69 | 66 | 73 | 66 |
| **32** | 77 | 62 | 29 | 67 | 89 | 80 | 95 | 113 | 48 | 126 | 89 | 85 | 69 | 109 |
| **31** | 72 | 34 | 47 | 138 | 96 | 74 | 116 | 76 | 82 | 88 | 135 | 62 | 98 | 142 |
| **30** | 37 | 35 | 61 | 54 | 89 | 69 | 153 | N/A | 63 | 94 | 104 | 57 | 40 | 90 |
| **29** | 24 | 67 | 24 | 64 | 61 | 77 | 113 | 74 | 99 | 63 | 166 | 22 | 43 | 47 |
| **28** | 35 | 70 | 41 | 39 | 82 | 73 | 68 | 120 | 73 | 52 | 177 | 78 | 109 | 60 |
| **27** | 41 | 34 | 35 | 78 | 123 | 76 | 87 | 91 | 101 | 142 | 102 | 33 | 92 | 46 |
| **26** | 39 | 38 | 45 | 80 | 120 | 66 | 158 | 144 | 95 | 92 | 62 | 39 | 115 | 104 |
| **25** | 25 | 54 | 31 | 59 | 69 | 71 | 46 | 132 | 35 | 49 | 155 | 43 | 138 | 42 |
| **24** | 41 | 58 | 38 | 100 | 120 | 78 | 81 | 150 | 71 | 80 | 125 | 68 | 91 | 51 |
| **23** | 52 | 65 | 19 | 95 | 87 | 80 | N/A | 117 | 69 | 183 | 150 | 44 | 75 | 46 |
| **22** | 46 | N/A | 30 | 73 | 72 | 72 | N/A | 143 | 68 | 108 | 35 | 61 | 66 | 69 |
| **21** | 54 | 73 | 37 | 82 | 68 | 90 | N/A | 81 | 23 | 85 | 154 | 55 | 78 | 87 |
| **20** | 62 | 47 | 30 | 100 | 93 | 105 | N/A | 77 | 10 | 7 | 100 | 38 | 48 | 53 |
| **19** | 55 | 63 | 58 | 75 | 123 | 73 | N/A | 100 | 65 | 83 | 136 | 46 | 90 | 73 |
| **18** | 82 | 55 | 44 | 59 | 178 | 60 | N/A | 99 | 6 | 46 | 84 | 38 | 90 | 48 |
| **17** | 50 | 31 | 50 | 57 | 81 | 44 | 155 | 77 | 53 | 110 | 102 | 55 | 40 | 41 |
| **16** | 35 | 68 | 23 | 81 | 80 | 41 | 79 | 111 | 72 | 87 | 101 | 64 | 66 | 39 |
| **15** | 41 | 46 | 5 | 64 | 96 | 51 | 140 | 81 | 48 | 43 | 138 | 60 | 94 | 31 |
| **14** | 32 | 62 | 28 | 38 | 153 | 45 | 198 | 78 | 58 | 105 | 91 | 31 | 103 | 47 |
| **13** | 42 | 67 | 8 | 129 | 36 | 49 | 94 | 43 | 56 | 91 | 101 | 63 | 55 | 32 |
| **12** | 19 | 79 | 37 | 109 | 86 | 82 | 96 | 49 | 71 | 70 | 121 | 62 | 104 | 38 |
| **11** | 38 | 55 | 43 | 43 | 73 | 79 | 107 | 98 | 65 | 119 | 92 | 105 | 69 | 36 |
| **10** | 46 | 56 | 30 | 69 | 83 | 64 | 139 | 89 | 89 | 111 | 70 | 26 | 88 | 38 |
| **9** | 60 | 23 | 47 | 87 | 107 | 49 | 89 | 86 | 83 | 103 | 68 | 72 | 98 | 20 |
| **8** | 45 | 107 | 38 | 71 | 62 | 54 | 108 | 91 | 77 | 41 | 89 | 61 | 135 | 74 |
| **7** | 56 | 92 | 68 | 68 | 47 | 83 | 90 | 75 | 45 | 16 | 72 | 41 | 52 | 63 |
| **6** | 51 | 42 | 22 | 29 | 77 | 55 | 100 | 63 | 45 | 36 | 62 | 40 | 48 | 44 |
| **5** | 54 | 74 | 50 | 76 | 81 | 54 | 48 | 44 | 77 | 59 | 81 | 63 | 36 | 26 |
| **4** | 24 | 74 | 38 | 32 | 47 | 53 | 52 | 62 | 46 | 57 | 45 | 6 | 38 | 18 |
| **3** | 26 | 30 | 8 | 74 | 60 | 34 | 46 | 114 | 8 | 47 | 57 | 25 | 44 | 32 |
| **2** | 31 | 22 | N/A | 38 | 67 | 13 | 57 | 107 | 8 | 14 | 63 | 21 | 17 | 30 |
| **1** | 30 | 100 | 6 | 57 | 45 | 26 | 25 | 66 | 37 | 76 | 76 | 11 | 50 | 23 |

### Table S17. Tree-level apple volume (m³) in the orchard on 27^th^ August 2024.

| **Col**  **Row** | **1** | **2** | **3** | **4** | **5** | **6** | **7** | **8** | **9** | **10** | **11** | **12** | **13** | **14** |
| --- | --- | --- | --- | --- | --- | --- | --- | --- | --- | --- | --- | --- | --- | --- |
| **80** | 0.123 | 0.024 | 0.031 | 0.177 | 0.303 | 0.057 | 0.23 | 0.093 | 0.018 | 0.271 | 0.177 | 0.159 | 0.096 | 0.038 |
| **79** | 0.046 | 0.059 | 0.007 | 0.211 | 0.14 | 0.026 | 0.141 | 0.189 | 0.048 | 0.206 | 0.246 | 0.105 | 0.178 | 0.092 |
| **78** | 0.057 | 0.119 | 0.091 | 0.297 | 0.103 | 0.095 | 0.214 | 0.211 | 0.117 | 0.191 | 0.264 | 0.146 | 0.099 | 0.039 |
| **77** | 0.057 | 0.108 | 0.139 | 0.148 | 0.24 | 0.126 | 0.24 | 0.346 | 0.263 | 0.282 | 0.317 | 0.103 | 0.168 | 0.079 |
| **76** | 0.092 | 0.21 | 0.099 | 0.166 | 0.113 | 0.085 | 0.414 | 0.292 | 0.156 | 0.416 | 0.316 | 0.144 | 0.159 | 0.1 |
| **75** | 0.077 | 0.183 | 0.066 | 0.342 | 0.222 | 0.188 | 0.397 | 0.292 | 0.19 | 0.229 | 0.414 | 0.134 | 0.251 | 0.091 |
| **74** | 0.123 | 0.102 | 0.063 | 0.233 | 0.157 | 0.144 | 0.228 | 0.365 | 0.182 | 0.214 | 0.413 | 0.125 | 0.317 | 0.277 |
| **73** | 0.216 | 0.163 | 0.053 | 0.21 | 0.324 | 0.176 | N/A | 0.278 | 0.103 | 0.322 | 0.174 | 0.213 | 0.315 | 0.239 |
| **72** | 0.151 | 0.169 | 0.07 | 0.198 | 0.17 | 0.181 | 0.135 | 0.334 | 0.17 | 0.319 | 0.21 | 0.168 | 0.293 | 0.13 |
| **71** | 0.062 | 0.237 | 0.108 | 0.237 | 0.247 | 0.159 | 0.212 | 0.284 | 0.195 | 0.273 | 0.364 | 0.154 | 0.313 | 0.193 |
| **70** | 0.166 | 0.121 | 0.086 | 0.295 | N/A | 0.103 | 0.289 | 0.306 | 0.132 | 0.415 | 0.29 | 0.191 | 0.289 | 0.154 |
| **69** | 0.137 | 0.091 | 0.127 | N/A | 0.301 | 0.117 | 0.446 | 0.241 | 0.174 | 0.331 | 0.315 | 0.21 | 0.265 | 0.266 |
| **68** | 0.174 | 0.08 | 0.098 | 0.152 | 0.294 | 0.187 | 0.365 | 0.432 | 0.205 | 0.34 | 0.331 | 0.096 | 0.266 | 0.195 |
| **67** | 0.103 | 0.102 | 0.068 | 0.163 | 0.281 | 0.132 | 0.311 | 0.279 | 0.18 | 0.348 | 0.264 | 0.176 | 0.173 | 0.123 |
| **66** | 0.167 | 0.164 | 0.134 | 0.135 | 0.302 | 0.148 | 0.364 | 0.105 | 0.224 | 0.436 | 0.36 | 0.141 | 0.227 | 0.125 |
| **65** | 0.145 | 0.175 | 0.039 | 0.233 | 0.275 | 0.146 | 0.32 | 0.195 | 0.154 | 0.278 | 0.301 | 0.111 | 0.138 | 0.164 |
| **64** | 0.131 | 0.166 | 0.051 | 0.139 | 0.211 | 0.176 | 0.36 | 0.181 | 0.138 | 0.327 | 0.322 | 0.121 | 0.231 | 0.141 |
| **63** | 0.084 | 0.095 | 0.084 | 0.126 | 0.292 | 0.135 | 0.37 | 0.217 | 0.162 | 0.278 | 0.313 | 0.133 | 0.232 | 0.25 |
| **62** | 0.151 | 0.157 | 0.105 | 0.244 | 0.267 | 0.094 | 0.108 | 0.252 | 0.14 | 0.243 | 0.226 | 0.122 | 0.34 | 0.146 |
| **61** | 0.182 | 0.222 | 0.04 | 0.215 | N/A | 0.131 | 0.218 | 0.29 | 0.163 | 0.247 | 0.218 | 0.077 | 0.173 | 0.265 |
| **60** | 0.126 | 0.089 | 0.11 | 0.271 | 0.16 | 0.125 | N/A | 0.174 | 0.14 | 0.401 | 0.346 | 0.092 | 0.183 | 0.159 |
| **59** | 0.115 | 0.152 | N/A | 0.255 | 0.237 | 0.109 | 0.254 | 0.27 | 0.157 | 0.237 | 0.203 | 0.121 | 0.219 | 0.174 |
| **58** | 0.207 | 0.138 | 0.107 | 0.147 | 0.254 | 0.057 | 0.265 | 0.206 | 0.134 | 0.363 | 0.254 | 0.146 | 0.318 | 0.159 |
| **57** | 0.088 | 0.082 | 0.102 | 0.124 | 0.276 | 0.103 | 0.223 | 0.25 | 0.109 | 0.297 | 0.32 | 0.1 | 0.226 | 0.272 |
| **56** | 0.14 | 0.169 | 0.07 | 0.213 | 0.217 | 0.074 | 0.409 | 0.324 | 0.097 | 0.201 | 0.178 | 0.117 | 0.235 | 0.18 |
| **55** | 0.184 | 0.14 | 0.059 | 0.08 | 0.285 | 0.07 | 0.206 | 0.174 | 0.143 | 0.233 | 0.303 | 0.059 | 0.206 | 0.213 |
| **54** | 0.103 | 0.16 | 0.09 | 0.169 | 0.246 | 0.11 | 0.218 | 0.198 | 0.143 | 0.253 | 0.248 | 0.098 | 0.297 | 0.142 |
| **53** | 0.113 | 0.162 | 0.088 | 0.096 | 0.242 | 0.064 | 0.219 | 0.233 | 0.106 | 0.357 | 0.193 | 0.079 | 0.243 | 0.22 |
| **52** | 0.234 | 0.155 | 0.075 | 0.131 | 0.128 | 0.089 | 0.121 | 0.154 | 0.139 | 0.351 | 0.281 | 0.135 | 0.155 | 0.139 |
| **51** | 0.096 | 0.148 | 0.067 | N/A | 0.134 | 0.11 | 0.247 | 0.162 | 0.091 | 0.31 | 0.302 | 0.111 | 0.238 | 0.17 |
| **50** | 0.164 | 0.093 | 0.067 | 0.173 | 0.241 | 0.185 | 0.248 | 0.181 | 0.087 | 0.278 | 0.297 | 0.144 | 0.165 | 0.159 |
| **49** | 0.14 | 0.151 | 0.034 | 0.187 | 0.233 | 0.069 | 0.249 | 0.129 | 0.127 | 0.25 | 0.258 | 0.097 | 0.175 | 0.111 |
| **48** | 0.033 | 0.084 | 0.013 | 0.136 | 0.122 | 0.111 | 0.146 | 0.138 | 0.122 | 0.248 | 0.26 | 0.104 | 0.109 | 0.161 |
| **47** | 0.146 | 0.154 | 0.049 | 0.059 | 0.349 | 0.109 | 0.122 | 0.202 | 0.149 | 0.236 | 0.236 | 0.121 | 0.25 | 0.212 |
| **46** | 0.122 | 0.193 | 0.046 | 0.22 | 0.196 | 0.124 | 0.254 | 0.167 | 0.1 | 0.364 | 0.275 | 0.131 | 0.154 | 0.185 |
| **45** | 0.13 | 0.155 | 0.006 | 0.156 | 0.051 | 0.143 | 0.289 | 0.15 | 0.165 | 0.244 | 0.278 | 0.157 | 0.104 | 0.147 |
| **44** | 0.095 | 0.126 | 0.111 | 0.259 | 0.184 | 0.084 | 0.111 | 0.409 | 0.175 | 0.206 | 0.306 | 0.096 | 0.241 | 0.233 |
| **43** | 0.099 | 0.181 | 0.078 | 0.289 | 0.269 | 0.179 | 0.249 | 0.042 | 0.142 | 0.325 | 0.279 | 0.126 | 0.184 | 0.174 |
| **42** | 0.099 | 0.071 | 0.11 | 0.172 | 0.224 | 0.165 | 0.276 | 0.141 | 0.167 | 0.317 | 0.273 | 0.132 | 0.198 | 0.13 |
| **41** | 0.117 | 0.167 | 0.102 | 0.195 | 0.245 | 0.137 | 0.353 | 0.402 | 0.125 | 0.32 | 0.269 | 0.108 | 0.194 | 0.17 |
| **40** | 0.154 | 0.184 | 0.1 | 0.393 | 0.259 | 0.105 | 0.259 | 0.16 | 0.184 | 0.296 | N/A | 0.083 | 0.192 | 0.108 |
| **39** | 0.103 | 0.157 | 0.076 | 0.353 | 0.204 | 0.067 | 0.234 | 0.342 | 0.165 | 0.291 | 0.181 | 0.129 | 0.116 | 0.192 |
| **38** | 0.146 | 0.101 | 0.12 | 0.233 | 0.239 | 0.122 | 0.306 | 0.274 | 0.117 | 0.159 | N/A | 0.107 | 0.214 | 0.146 |
| **37** | 0.204 | 0.174 | 0.068 | 0.197 | 0.235 | 0.086 | 0.265 | 0.266 | 0.009 | 0.161 | 0.073 | 0.2 | 0.173 | 0.205 |
| **36** | 0.136 | 0.094 | 0.058 | 0.152 | 0.189 | 0.109 | 0.306 | 0.205 | 0.159 | 0.256 | 0.35 | 0.118 | 0.239 | 0.19 |
| **35** | 0.09 | 0.277 | 0.137 | 0.329 | 0.227 | 0.163 | 0.336 | 0.261 | 0.189 | 0.162 | 0.351 | 0.124 | 0.249 | 0.159 |
| **34** | 0.181 | 0.079 | 0.11 | 0.103 | 0.265 | 0.088 | 0.29 | 0.168 | 0.144 | 0.101 | 0.447 | 0.135 | 0.324 | 0.164 |
| **33** | 0.162 | 0.102 | 0.074 | 0.09 | 0.213 | 0.116 | 0.358 | 0.239 | 0.178 | 0.278 | 0.145 | 0.105 | 0.166 | 0.173 |
| **32** | 0.138 | 0.187 | 0.069 | 0.19 | 0.173 | 0.133 | 0.24 | 0.305 | 0.099 | 0.304 | 0.204 | 0.148 | 0.147 | 0.228 |
| **31** | 0.168 | 0.095 | 0.09 | 0.349 | 0.288 | 0.144 | 0.289 | 0.243 | 0.15 | 0.195 | 0.356 | 0.109 | 0.291 | 0.264 |
| **30** | 0.093 | 0.115 | 0.126 | 0.084 | 0.269 | 0.177 | 0.451 | N/A | 0.131 | 0.199 | 0.206 | 0.144 | 0.118 | 0.2 |
| **29** | 0.067 | 0.138 | 0.074 | 0.139 | 0.192 | 0.134 | 0.346 | 0.174 | 0.209 | 0.172 | 0.308 | 0.059 | 0.108 | 0.096 |
| **28** | 0.076 | 0.149 | 0.097 | 0.142 | 0.271 | 0.186 | 0.205 | 0.406 | 0.154 | 0.127 | 0.459 | 0.179 | 0.284 | 0.136 |
| **27** | 0.109 | 0.085 | 0.077 | 0.277 | 0.315 | 0.164 | 0.302 | 0.29 | 0.21 | 0.329 | 0.426 | 0.084 | 0.254 | 0.115 |
| **26** | 0.085 | 0.149 | 0.084 | 0.176 | 0.326 | 0.141 | 0.422 | 0.405 | 0.198 | 0.245 | 0.155 | 0.071 | 0.237 | 0.23 |
| **25** | 0.062 | 0.129 | 0.08 | 0.158 | 0.132 | 0.14 | 0.197 | 0.327 | 0.089 | 0.126 | 0.437 | 0.105 | 0.319 | 0.094 |
| **24** | 0.13 | 0.166 | 0.069 | 0.278 | 0.362 | 0.16 | 0.237 | 0.483 | 0.15 | 0.242 | 0.258 | 0.141 | 0.182 | 0.135 |
| **23** | 0.147 | 0.157 | 0.031 | 0.219 | 0.186 | 0.187 | N/A | 0.346 | 0.125 | 0.405 | 0.363 | 0.119 | 0.228 | 0.088 |
| **22** | 0.171 | N/A | 0.053 | 0.195 | 0.19 | 0.189 | N/A | 0.458 | 0.154 | 0.31 | 0.096 | 0.124 | 0.167 | 0.116 |
| **21** | 0.179 | 0.297 | 0.085 | 0.225 | 0.171 | 0.179 | N/A | 0.222 | 0.047 | 0.186 | 0.429 | 0.094 | 0.149 | 0.193 |
| **20** | 0.141 | 0.115 | 0.081 | 0.352 | 0.184 | 0.236 | N/A | 0.213 | 0.015 | 0.016 | 0.234 | 0.105 | 0.143 | 0.098 |
| **19** | 0.111 | 0.139 | 0.143 | 0.211 | 0.37 | 0.153 | N/A | 0.278 | 0.111 | 0.301 | 0.371 | 0.126 | 0.219 | 0.19 |
| **18** | 0.172 | 0.149 | 0.102 | 0.123 | 0.353 | 0.091 | N/A | 0.248 | 0.011 | 0.133 | 0.173 | 0.073 | 0.196 | 0.098 |
| **17** | 0.119 | 0.087 | 0.084 | 0.13 | 0.241 | 0.108 | 0.33 | 0.183 | 0.118 | 0.233 | 0.302 | 0.144 | 0.084 | 0.065 |
| **16** | 0.121 | 0.222 | 0.062 | 0.229 | 0.242 | 0.09 | 0.222 | 0.285 | 0.126 | 0.229 | 0.161 | 0.102 | 0.128 | 0.092 |
| **15** | 0.126 | 0.087 | 0.007 | 0.152 | 0.206 | 0.093 | 0.406 | 0.14 | 0.114 | 0.136 | 0.316 | 0.131 | 0.199 | 0.058 |
| **14** | 0.094 | 0.194 | 0.062 | 0.132 | 0.408 | 0.096 | 0.487 | 0.17 | 0.121 | 0.289 | 0.217 | 0.085 | 0.222 | 0.118 |
| **13** | 0.101 | 0.175 | 0.019 | 0.312 | 0.098 | 0.114 | 0.265 | 0.109 | 0.096 | 0.271 | 0.238 | 0.143 | 0.096 | 0.078 |
| **12** | 0.043 | 0.231 | 0.065 | 0.276 | 0.317 | 0.198 | 0.296 | 0.097 | 0.146 | 0.232 | 0.258 | 0.129 | 0.223 | 0.062 |
| **11** | 0.098 | 0.241 | 0.077 | 0.121 | 0.19 | 0.177 | 0.272 | 0.227 | 0.145 | 0.259 | 0.272 | 0.199 | 0.163 | 0.072 |
| **10** | 0.101 | 0.194 | 0.09 | 0.226 | 0.153 | 0.183 | 0.337 | 0.262 | 0.168 | 0.266 | 0.143 | 0.046 | 0.213 | 0.084 |
| **9** | 0.135 | 0.063 | 0.112 | 0.231 | 0.271 | 0.077 | 0.224 | 0.191 | 0.188 | 0.254 | 0.162 | 0.136 | 0.235 | 0.042 |
| **8** | 0.126 | 0.217 | 0.083 | 0.267 | 0.196 | 0.135 | 0.243 | 0.222 | 0.149 | 0.083 | 0.212 | 0.104 | 0.273 | 0.157 |
| **7** | 0.155 | 0.204 | 0.123 | 0.151 | 0.082 | 0.18 | 0.165 | 0.237 | 0.106 | 0.027 | 0.168 | 0.076 | 0.105 | 0.12 |
| **6** | 0.135 | 0.091 | 0.045 | 0.095 | 0.156 | 0.104 | 0.241 | 0.18 | 0.096 | 0.056 | 0.137 | 0.073 | 0.108 | 0.093 |
| **5** | 0.12 | 0.149 | 0.097 | 0.176 | 0.2 | 0.11 | 0.1 | 0.1 | 0.155 | 0.125 | 0.21 | 0.109 | 0.071 | 0.075 |
| **4** | 0.061 | 0.133 | 0.072 | 0.085 | 0.152 | 0.117 | 0.151 | 0.096 | 0.102 | 0.102 | 0.107 | 0.009 | 0.088 | 0.036 |
| **3** | 0.081 | 0.099 | 0.01 | 0.169 | 0.195 | 0.067 | 0.129 | 0.261 | 0.011 | 0.159 | 0.154 | 0.055 | 0.103 | 0.056 |
| **2** | 0.076 | 0.066 | N/A | 0.109 | 0.118 | 0.014 | 0.115 | 0.256 | 0.015 | 0.03 | 0.17 | 0.041 | 0.045 | 0.057 |
| **1** | 0.11 | 0.179 | 0.023 | 0.145 | 0.151 | 0.047 | 0.093 | 0.191 | 0.109 | 0.207 | 0.216 | 0.024 | 0.114 | 0.059 |

# References:

Du, S., Lindenbergh, R., Ledoux, H., Stoter, J., and Nan, L. (2019) *AdTree: Accurate, detailed, and automatic modelling of laser-scanned trees*. *Remote Sens.*, **11**, 1–19.

Jones, M.W., Bærentzen, J.A., and Sramek, M. (2006) *3D distance fields: A survey of techniques and applications*. *IEEE Trans. Vis. Comput. Graph.*, **12**, 518–599.

Karger, D.R. and Ruhl, M. (2002) *Finding nearest neighbors in growth-restricted metrics*. In: *Conference Proceedings of the Annual ACM Symposium on Theory of Computing* , pp. 741–750.

Korzeniowska, K. and Łącka, M. (2011) *Generating DEM from LiDAR data–comparison of available software tools*. *Arch. Fotogram. Kartogr. i Teledetekcji*, **22**, 271–284.

Meyer, L., Gilson, A., Scholz, O., and Stamminger, M. (2023) *CherryPicker: Semantic Skeletonization and Topological Reconstruction of Cherry Trees*. *IEEE Comput. Soc. Conf. Comput. Vis. Pattern Recognit. Work.*, **2023**-**June**, 6244–6253.

Novotni, M. and Klein, R. (2001) *A geometric approach to 3D object comparison*. In: *Proceedings - International Conference on Shape Modeling and Applications, SMI 2001* , pp. 167–175.

Qiu, T., Wang, T., Han, T., Kuehn, K., Cheng, L., Meng, C., et al. (2024) *AppleQSM: Geometry-Based 3D Characterization of Apple Tree Architecture in Orchards*. *Plant Phenomics*, **6**, 1–18.

Raumonen, P., Kaasalainen, M., Markku, Å., Kaasalainen, S., Kaartinen, H., Vastaranta, M., et al. (2013) *Fast automatic precision tree models from terrestrial laser scanner data*. *Remote Sens.*, **5**, 491–520.

Shekhar, S. and Xiong, H. (2008) *Dijktra’s Shortest Path Algorithm*. In: *Encyclopedia of GIS* , pp. 240–240.

Shipman, J.W. (2013) *Tkinter 8.5 reference: a GUI for Python*. New Mexico: New Mexico Tech Computer Center.

Sun, G., Lu, H., Zhao, Y., Zhou, Jie, Jackson, R., Wang, Y., et al. (2022) *AirMeasurer: open-source software to quantify static and dynamic traits derived from multiseason aerial phenotyping to empower genetic mapping studies in rice*. *New Phytol.*, **236**, 1584–1604.

Zhu, Y., Sun, G., Ding, G., Zhou, J., Wen, M., Jin, S., et al. (2021) *Large-scale field phenotyping using backpack LiDAR and CropQuant-3D to measure structural variation in wheat*. *Plant Physiol.*, **187**, 716–738.
